# Chemoprophylaxis, diagnosis, treatments, and discharge management of COVID-19: An evidence-based clinical practice guideline (updated version)

**DOI:** 10.1186/s40779-020-00270-8

**Published:** 2020-09-04

**Authors:** Ying-Hui Jin, Qing-Yuan Zhan, Zhi-Yong Peng, Xue-Qun Ren, Xun-Tao Yin, Lin Cai, Yu-Feng Yuan, Ji-Rong Yue, Xiao-Chun Zhang, Qi-Wen Yang, Jianguang Ji, Jian Xia, Yi-Rong Li, Fu-Xiang Zhou, Ya-Dong Gao, Zhui Yu, Feng Xu, Ming-Li Tu, Li-Ming Tan, Min Yang, Fang Chen, Xiao-Ju Zhang, Mei Zeng, Yu Zhu, Xin-Can Liu, Jian Yang, Dong-Chi Zhao, Yu-Feng Ding, Ning Hou, Fu-Bing Wang, Hao Chen, Yong-Gang Zhang, Wei Li, Wen Chen, Yue-Xian Shi, Xiu-Zhi Yang, Xue-Jun Wang, Yan-Jun Zhong, Ming-Juan Zhao, Bing-Hui Li, Lin-Lu Ma, Hao Zi, Na Wang, Yun-Yun Wang, Shao-Fu Yu, Lu-Yao Li, Qiao Huang, Hong Weng, Xiang-Ying Ren, Li-Sha Luo, Man-Ru Fan, Di Huang, Hong-Yang Xue, Lin-Xin Yu, Jin-Ping Gao, Tong Deng, Xian-Tao Zeng, Hong-Jun Li, Zhen-Shun Cheng, Xiaomei Yao, Xing-Huan Wang, Evidence-Based Medicine Chapter of China International Exchange and Promotive Association for Medical and Health Care (CPAM), Chinese Research Hospital Association (CRHA)

**Affiliations:** 1 Center for Evidence-Based and Translational Medicine, Zhongnan Hospital of Wuhan University, Wuhan, 430071 China; 2 National Clinical Research Center for Respiratory Diseases, China-Japan Friendship Hospital, Beijing, 100029 China; 3 Department of Pulmonary and Critical Care Medicine, China-Japan Friendship Hospital, Beijing, 10029 China; 4 Leishenshan Hospital in Wuhan, Wuhan, 430200 China; 5 Department of Critical Care Medicine, Zhongnan Hospital of Wuhan University, Wuhan, 430071 China; 6 Institutes of Evidence-based Medicine and Knowledge Translation, Henan University, Kaifeng, 475000 Henan China; 7 Department of Medical Imaging, Guizhou Provincial People’s Hospital, Guiyang, 550002 China; 8 Departments of Orthopedics, Zhongnan Hospital of Wuhan University, Wuhan, 430071 China; 9 Department of Hepatobiliary Surgery, Zhongnan Hospital of Wuhan University, Wuhan, 430071 China; 10 National Clinical Research Center for Geriatrics, West China Hospital of Sichuan University, Chengdu, 610041 China; 11 Department of Geriatrics, West China Hospital of Sichuan University, Chengdu, 610041 China; 12 Department of Radiology, Zhongnan Hospital of Wuhan University, Wuhan, 430071 China; 13 Department of Clinical Laboratory, Peking Union Medical College Hospital (PUMCH), Peking Union Medical College, Chinese Academy of Medical Sciences (CAMS), Beijing, 100730 China; 14 Center for Primary Health Care Research, Lund University and Region Skåne, 25002 Malmö, Sweden; 15 Emergency Center, Zhongnan Hospital of Wuhan University, Wuhan, 403371 China; 16 Department of Allergology, Zhongnan Hospital of Wuhan University, Wuhan, 430071 China; 17 Department of Radiation and Medical Oncology, Zhongnan Hospital of Wuhan University, Wuhan, 43071 China; 18 Department of Critical Care Medicine, Renmin Hospital of Wuhan University, Wuhan, 430060 China; 19 Department of Emergency Medicine and Chest Pain Center, Qilu Hospital of Shandong University, Jinan, 250002 China; 20 Department of Respiratory and Critical Care Medicine, Suizhou Central Hospital, Hubei University of Medicine, Suizhou, 441300 Hubei China; 21 Department of Clinic Pharmacy, Second People’s Hospital of Huaihua City, Huaihua, 418000 Hunan China; 22 Department of Intensive Care Unit, The Second Affiliated Hospital of Anhui Medical University, Hefei, 230601 China; 23 Department of Internal Medicine, Zhengzhou University Hospital, Zhengzhou, 450001 China; 24 Department of Respiratory and Critical Care Medicine, Henan Provincial People’s Hospital, Zhengzhou, 450003 China; 25 Department of Infectious Diseases, Children’s Hospital of Fudan University, Shanghai, 201102 China; 26 Department of Infectious Disease, West China Second Hospital, Sichuan University, Chengdu, 610041 China; 27 Department of Cardiology, The First Affiliated Hospital of Henan University of Chinese Medicine, Zhengzhou, 450000 China; 28 Department of Cardiology, Yichang NO.1 Hospital, Renmin Hospital of China Three Gorges University, Yichang, 443000 Hubei China; 29 Department of Pediatrics, Zhongnan Hospital of Wuhan University, Wuhan, 430071 China; 30 Department of Pharmacy, Tongji Hospital, Tongji Medical College, Huazhong University of Science and Technology, Wuhan, 430030 China; 31 Department of Pharmacy, Shandong Provincial Hospital, Shandong First Medical University & Shandong Academy of Medical Sciences, Jinan, 250021 China; 32 Laboratory of Integrated Acupuncture and Drugs, Nanjing University of Chinese Medicine, Nanjing, 210023 China; 33 Department of Periodical Press, West China Hospital, Sichuan University, Chengdu, 610041 China; 34 Department of Clinical Laboratory, Yantai Yuhuangding Hospital, Qingdao University, Yantai, 264000 Shandong China; 35 Department of Radiology, Taihe Hospital, Hubei University of Medicine, Shiyan, 442000 Hubei China; 36 School of Nursing, Peking University, Beijing, 100191 China; 37 Department of Respiratory and Critical Care Medicine, Kaifeng Central Hospital, Kaifeng, 475000 Henan China; 38 Department of Emergency, Beijing Electric Power Hospital, Beijing, 100073 China; 39 ICU Center, The Second Xiangya Hospital, Central South University, Changsha, 410008 China; 40 Department of Urology, Zhongnan Hospital of Wuhan University, Wuhan, 430071 China; 41 Department of Haematology, Zhongnan Hospital, Wuhan University, Wuhan, 430071 China; 42 College of Nursing and Health, Henan Medical School, Henan University, Kaifeng, 475000 Henan China; 43 School of Nursing, Shanxi Medical University, Taiyuan, 030001 China; 44 Department of Diagnostic Radiology, Beijing You’an Hospital, Capital Medical University, Beijing, 100069 China; 45 Department of Respiratory Medicine, Zhongnan Hospital of Wuhan University, Wuhan, 430071 China; 46 Department of Health Research Methods, Evidence, and Impact, McMaster University, Hamilton, Ontario L8S 4L8 Canada

**Keywords:** COVID-19, SARS-CoV-2, Recommendation, Chemoprophylaxis, Diagnosis, Treatment, Discharge management, Traditional Chinese medicine; guideline

## Abstract

The novel severe acute respiratory syndrome coronavirus 2 (SARS-CoV-2) is the cause of a rapidly spreading illness, coronavirus disease 2019 (COVID-19), affecting more than seventeen million people around the world. Diagnosis and treatment guidelines for clinicians caring for patients are needed. In the early stage, we have issued “A rapid advice guideline for the diagnosis and treatment of 2019 novel coronavirus (2019-nCoV) infected pneumonia (standard version)”; now there are many direct evidences emerged and may change some of previous recommendations and it is ripe for develop an evidence-based guideline. We formed a working group of clinical experts and methodologists. The steering group members proposed 29 questions that are relevant to the management of COVID-19 covering the following areas: chemoprophylaxis, diagnosis, treatments, and discharge management. We searched the literature for direct evidence on the management of COVID-19, and assessed its certainty generated recommendations using the Grading of Recommendations, Assessment, Development and Evaluation (GRADE) approach. Recommendations were either strong or weak, or in the form of ungraded consensus-based statement. Finally, we issued 34 statements. Among them, 6 were strong recommendations for, 14 were weak recommendations for, 3 were weak recommendations against and 11 were ungraded consensus-based statement. They covered topics of chemoprophylaxis (including agents and Traditional Chinese Medicine (TCM) agents), diagnosis (including clinical manifestations, reverse transcription-polymerase chain reaction (RT-PCR), respiratory tract specimens, IgM and IgG antibody tests, chest computed tomography, chest x-ray, and CT features of asymptomatic infections), treatments (including lopinavir-ritonavir, umifenovir, favipiravir, interferon, remdesivir, combination of antiviral drugs, hydroxychloroquine/chloroquine, interleukin-6 inhibitors, interleukin-1 inhibitors, glucocorticoid, qingfei paidu decoction, lianhua qingwen granules/capsules, convalescent plasma, lung transplantation, invasive or noninvasive ventilation, and extracorporeal membrane oxygenation (ECMO)), and discharge management (including discharge criteria and management plan in patients whose RT-PCR retesting shows SARS-CoV-2 positive after discharge). We also created two figures of these recommendations for the implementation purpose. We hope these recommendations can help support healthcare workers caring for COVID-19 patients.

## Background

On March 11th 2020, the World Health Organization (WHO) declared Corona Virus Disease 2019 (COVID-19) a pandemic. There has been 17,106,007confirmed cases of COVID-19 globally, including 668,910 deaths, reported to WHO as of 4:39 pm CEST, 31 July 2020 [1]. Given the current global public health threat and economic impact, chemoprophylaxis, fast diagnosis, therapeutic measures, and discharge management are all-important. Early in the COVID-19 outbreak, we published a rapid advice guideline [2] for the diagnosis and treatment of COVID-19 following the WHO Rapid Advice Guideline Handbook [3]. In the absence of direct published evidence, our recommendations were primarily based on clinical expert evidence and indirect evidence (such as Severe Acute Respiratory Syndrome [SARS] or Middle East Respiratory Syndrome [MERS]) up to the end of January 2020. Recently, a number of research papers are being published both in China and abroad providing research evidence for managing COVID-19 that can change some of our previous recommendations and motivate us to update our guideline.

This updated guideline includes four sections: Chemoprophylaxis, Diagnosis, Treatments, and Discharge Management.

## Methods

### Target users

Frontline clinicians and policymakers involve in the care of patients with COVID-19. The guideline applies to all income settings.

### Target population

Adult patients (≥18 years) with any clinical types of COVID-19 (pregnant women were not included).

### Composition of the guideline development group

The guideline panel was composed of a steering group, working group, and an evidence synthesis group, which included 27 clinical experts (expertise in respiratory medicine, infectious disease, critical care medicine, cardiology, emergency medicine, pediatrics, oncology, gerontology, laboratory medicine, medical imaging, clinical immunology, and clinical pharmacy), six methodologists, and 18 clinical research assistants with evidence searching and assessment. The external review group included 9 clinical experts and one methodologist. (See the Authors’ Contributions).

### Conflict of interest policy

All guideline panel members signed a confidentiality agreement and disclosed all potential conflicts of interest (Survey form see Additional file 1).

### Question generation

The initial “Structural Overview and Research Questions for Diagnosis and Treatment of COVID-19” were developed by the steering group members and were discussed in detail by the working group members. Eventually, 29 research questions were finalized after an online discussion, and guideline protocol has been published in *New Medicine* (Chinese name: Yixue Xinzhi Zazhi; http://www.jnewmed.com/) in China [4].

### Evidence review and development of clinical recommendations

We searched the bibliographic databases: PubMed, Embase, Cochrane library, CNKI (China National Knowledge Infrastructure) and Wanfang Databases. In addition, we searched recently up-to-date medical journals, preprint platforms, and platforms of clinical trial registry (search resources and websites see Additional file 2). The methodologists designed search strategies (Additional file 3) using medical subject heading keywords and text words in Chinese and English for all direct evidence defined as systematic review or meta-analysis, original studies with no language limitation. For questions of chemoprophylaxis and treatments, we excluded single-arm study and case reports. The first search was from December 1, 2019 to June6, 2020. Search for systematic reviews and primary articles were updated daily until July 8, 2020.

The risk of bias or quality assessment was based on the international evaluation standards of the corresponding literature, ROB 2.0 for randomized controlled trial (RCT); QUADAS-2 for diagnostic accuracy study; ROBINS-I for non-randomized comparative intervention studies [5]. Before the literature search, outcomes of treatment were ranked by the guideline panel classifying their importance as critical, important, and less important according to the GRADE (Grading of Recommendations Assessment, Development and Evaluation) approach [6]. For treatment questions, the critical outcomes prioritized for this guideline were mortality, critical conversion rate, incidence rate or time of intensive care unit (ICU) admission, and sequential organ failure assessment (SOFA). The important outcomes were oxygenation index/oxyhemoglobin saturation, time/rate positive-to-negative conversion of RT-PCR test for SARS coronavirus 2 (SARS-CoV-2), chest or lung imaging improvement or lesion absorption time or ratio, time to clinical improvement, clinical cure time or rate, pneumonia severity index (PSI), body temperature/time for body temperature to return to normal, duration of hospital stay, incidence rate or time of mechanical ventilation, and viral load. For diagnostic questions, the diagnostic accuracy outcomes (such as sensitivity, specificity, and AUC [area under curve]) were regarded as important outcomes.

Following the GRADE principles, the guideline panel rated the certainty of evidence for each outcome as “high,” “moderate,” “low,” or “very low”. Recommendations were graded based on the GRADE approach (Table 1) [6].

**Table 1:** Classification of evidence and recommendation

Grade of Recommendation	Strength of the recommendation: Benefits vs. risks, harms, and burdens (Grade 1 or 2)	State of the scientific evidence: Methodologic strength of supporting evidence (Grade A, B, or C)^a^	Implications
1A:Strong recommendation, high-quality evidence	Benefits clearly outweigh risk and burden, or vice versa	Consistent evidence from RCTs without important limitations or exceptionally strong evidence from observational studies	Recommendation can apply to most patients in most circumstances. Further research is very unlikely to change our confidence in the estimate of effect
1B: Strong recommendation, moderate-quality evidence	Benefits clearly outweigh risk and burden, or vice versa	Evidence from RCTs with important limitations (inconsistent results, methodologic flaws, indirect or imprecise), or very strong evidence from observational studies	Recommendation can apply to most patients in most circumstances. Higher quality research may well have an important impact on our confidence in the estimate of effect and may change the estimate
1C: Strong recommendation, low or very low-quality evidence	Benefits clearly outweigh risk and burden, or vice versa	Evidence for at least one critical outcome from observational studies, case series, or from RCTs with serious flaws or indirect evidence	Recommendation can apply to most patients in many circumstances. Higher quality research is likely to have an important impact on our confidence in the estimate of effect and may well change the estimate
2A: Weak recommendation, high-quality evidence	Benefits closely balanced with risks and burden	Consistent evidence from RCTs without important limitations or exceptionally strong evidence from observational studies	The best action may differ depending on circumstances or patients’ or societal values. Further research is very unlikely to change our confidence in the estimate of effect
2B: Weak recommendation, moderate-quality evidence	Benefits closely balanced with risks and burden	Evidence from RCTs with important limitations (inconsistent results, methodologic flaws, indirect or imprecise), or very strong evidence from observational studies	Best action may differ depending on circumstances or patients’ or societal values. Higher quality research may well have an important impact on our confidence in the estimate of effect and may change the estimate.
2C: Weak recommendation, low or very low-quality evidence	Uncertainty in the estimates of benefits, risks, and burden; benefits, risk, and burden may be closely balanced	Evidence for at least one critical outcome from observational studies, case series, or from RCTs with serious flaws or indirect evidence	Other alternatives may be equally reasonable. Higher quality research is likely to have an important impact on our confidence in the estimate of effect and may well change the estimate
Ungraded consensus-based statement	Uncertainty due to lack of evidence but expert opinion that benefits outweigh risk and burdens or vice versa	Insufficient evidence for a graded recommendation	Future research may well have an important impact on our confidence in the estimate of effect and may change the estimate

^a^Guideline panels determine the overall quality of evidence across all the critical outcomes essential based on: 1) If the quality of evidence is the same for all critical outcomes, then this becomes the overall quality of the evidence supporting the answer to the question; 2) If the quality of evidence differs and there is inconsistent results across critical outcomes, the interval quality of evidence is supplied; 3) If the quality of evidence differs and there is consistent results across critical outcomes, the lowest quality of evidence for any of the critical outcomes determines the overall quality of evidence

The simplified Evidence to Decision framework was considered in recommendation development: quality of the evidence, balance of desirable and undesirable consequences, acceptability of intervention to stakeholders, and feasibility of implementation [7, 8].

Based on these rules, the guideline panel members formulated the clinical recommendations establishing their strength by online discussion, reaching consensus where required by voting. For a recommendation to be graded as strong or weak, at least 70% of participants were required to endorse it. The guideline was reported using the AGREE Reporting Checklist [9] and Reporting Items for practice Guidelines in Healthcare (RIGHT) Reporting Checklist [10].

## Results

We finally used evidence of 75 original articles (included 12 RCTs), 33 systematic reviews or meta-analyses (See flow chart in literature searching in Additional files 4 and 5). We issued 34 statements. Among them, 6 were strong recommendations for, 14 were weak recommendations for, 3 were weak recommendations against and 11 were ungraded consensus-based statement.

Patients with COVID-19 were classified into four categories according to their clinical presentations: 1) Mild Type, the clinical symptoms are mild with no pneumonia manifestations found in imaging. 2) Moderate Type, patients have symptoms such as fever and respiratory tract symptoms with pneumonia manifestations seen on imaging. 3) Severe Type, adults who meet any of the following criteria: respiratory rate ≥ 30 breaths/min; oxygen saturations ≤93% in resting state; arterial partial pressure of oxygen (PaO_2_)/oxygen concentration (FiO_2_) ≤300 mmHg. Patients with > 50% lesions progression within 24 to 48 h in lung imaging should be treated as severe cases. 4) Critical Type, meeting any of the following criteria: occurrence of respiratory failure requiring mechanical ventilation; presence of shock; other organ failure that requires monitoring and treatment in the ICU.

The traditional GRADE summary tables for each outcome only presented in evidence body with pooled effect because of different disease types, interventions, doses, medication courses, and reported time of outcome as stated in some questions. We excluded single-arm study and case reports except for question of lung transplantation. Quality of evidence assessed by GRADE for pooled effect of outcomes of interest see Additional file 6. Recommendations list see Additional file 7. Results of methodological quality assessment of included studies and report of the external review panel will provide when for request.

### Chemoprophylaxis

#### Question 1: Which kind of agents can prevent COVID-19 in pre-exposure population to reduce SARS-CoV-2 infection?

##### Recommendation

There is insufficient evidence to for or against any agents to pre-exposure population (Grade2C).

##### Evidence summary

A retrospective cohort study including 106 healthcare workers indicated that as for 54 health care personnel before being exposed to their first COVID-19 patients, taking pre-exposure hydroxychloroquine prophylaxis was associated with an 80.7% reduction in the risk of acquiring a SARS-CoV-2 infection (RR = 0.193; 95% CI = 0.071–0.526; *P =* 0.001) compared with those who were not on it. Adverse effects, mostly mild, were recorded in 29.8% of those on hydroxychloroquine prophylaxis (gastrointestinal upset: 19.1%; skin rash: 6.4%; headache: 4.3%) [11]. The quality of evidence was very low due to lack of adequate information relevant to study design, for example, co-interventions balanced across intervention groups, start time of follow-up and start of intervention for most participants and imprecision of evidence quality.

##### Justification

Based on very low quality evidence, the panel did not suggest for or against hydroxychloroquine to prevent COVID-19 in pre-exposure population to reduce SARS-CoV-2 infection.

#### Question 2: Which kind of Traditional Chinese Medicine (TCM) agents can prevent COVID-19 in pre-exposure populations to reduce SARS-CoV-2 infection?

##### Recommendation

There is no evidence to for or against using any TCM agents for preventing COVID-19 in pre-exposure populations (Ungraded Consensus-Based Statement).

#### Question 3: Which kind of agents can prevent COVID-19 in post-exposure population (who contacted or took care of patients with COVID-19) to reduce SARS-CoV-2 infection?

##### Recommendation

There is insufficient evidence to for or against any agents to post-exposure population (Grade2C).

##### Evidence summary

A randomized trial included 821 participants who had household or occupational exposure to a person with confirmed COVID-19 at a distance of less than 6 ft. for more than 10 min while wearing neither a face mask nor an eye shield (high-risk exposure) or while wearing a face mask but no eye shield (moderate-risk exposure). The incidence of COVID-19 did not differ significantly between participants receiving hydroxychloroquine within 4 days after exposure (49 of 414 [11.8%]) and those receiving placebo (58 of 407 [14.3%]); the absolute difference was − 2.4 percentage points (95% CI, − 7.0 to 2.2; *P =* 0.35). Side effects (such as nausea, upset stomach, diarrhea, abdominal discomfort, or vomiting) were more common with hydroxychloroquine than with placebo (40.1% vs. 16.8%), but no serious adverse reactions were reported [12].

In addition, a retrospective cohort study including a total of 66 members in 27 families and 124 health care workers had evidence of close exposure to patients with confirmed COVID-19 revealed that compared with health care workers in Wuhan Union Hospital initially exposed to a cluster of COVID-19 infected colleagues without standard respiratory protection, as for the participants, Arbidol was a protective factor against the development of COVID-19 (HR 0.025, 95% CI 0.003–0.209, *P =* 0.0006 for family members and HR 0.056, 95% CI 0.005–0.662, *P =* 0.0221 for health care workers) [13]. However, the quality of evidence was low due to lack of adequate information relevant to study design, for example, co-interventions balanced across intervention groups, start time of follow-up and start of intervention for most participants and limited sample size, although large magnitude of an effect of decreasing incidence rate was observed.

##### Justification

We downgraded quality of evidence based on risk of bias and imprecision and did not upgrade evidence quality. Based on low quality evidence, the panel did not draw any recommendations for or against Arbidol or hydroxychloroquine to prevent COVID-19 in post-exposure population to reduce SARS-CoV-2 infection.

#### Question 4: Which kind of TCM agents can prevent COVID-19 in post-exposure populations (who contacted or took care of patients with COVID-19) to reduce SARS-CoV-2 infection?

##### Recommendation

There is no evidence to for or against using any TCM agents for preventing COVID-19 in in post-exposure populations (Ungraded Consensus-Based Statement).

### Diagnosis

#### Question 5: What are the typical clinical manifestations that can assist clinicians to differentiate SARS-CoV-2 infection from other viral infection in people with suspicious COVID-19?

##### Recommendations

The initial symptoms of COVID-19 in ordinary adult patients are most commonly fever and cough (mainly dry cough), often accompanied by fatigue, muscle soreness, dyspnea, expectoration and chest distress. In addition, some patients may present with ocular symptoms, cutaneous symptoms and gastrointestinal symptoms such as diarrhea, nausea, vomiting, olfactory and gustatory dysfunctions. From the perspective of Traditional Chinese Medicine clinical characteristics, the most common tongue body, tongue coating and pulse patterns were red tongue, greasy coating and deep pulse, respectively. If clinicians find that the patient has above-mentioned symptoms during the initial diagnosis, further examination (e.g. CT examination, nucleic acid test etc.) is required to confirm the diagnosis (Grade1A).

Asymptomatic patients generally remain asymptomatic or develop mild symptoms after admission, and clinicians should be cautious about the aggravation of symptoms in these patients. Critical-type patients have severe clinical manifestations and are more prone to fever, dyspnea and abdominal pain, and clinicians should identify the specific manifestations of critical patients as early as possible. (Grade2C).

##### Evidence summary

Common clinical manifestations: Ten systematic reviews/meta-analyses (134,222 patients from China, Australia, Italy, Japan, Korea, Netherlands, Singapore, UK, USA, Nepal, South Korea and Vietnam) showed that the most common symptoms of COVID-19 patients were fever (78.0–91.3%) [14–23], cough (52.0–72.2%) [14–23], myalgia or fatigue (16.7–51.0%) [14–23], dyspnea (10.4–45.6%) [17, 18, 20, 22, 23], expectoration (21.3–41.8%) [14, 18, 22] and chest distress (31.2%) [20].

Gastrointestinal symptoms: Four systematic reviews/meta-analyses (19,007 patients from China, USA, South Korea, Singapore, UK, Australia, Belgium, Cambodia, France, Germany, Italy, Japan, Malaysia, Nepal, Philippines, Russia, Thailand and Vietnam) showed that the pooled prevalence of digestive symptoms was 9.8–17.6% [24–26], with diarrhea (7.8–10.4%) [24, 25, 27], nausea or vomiting (5.5–7.7%) [24, 25, 27], abdominal discomfort/pain (3.0–6.9%) [24, 25] and loss of appetite (11%) [25] being the most common symptoms.

Severe-type patients: Two systematic review and meta-analysis (7827 patients from China) showed that the severe group had a higher risk of fever (*OR* = 1.67, 95% CI 1.15–2.42, *P =* 0.007, *I*^*2*^ = 38.8%) [28], dyspnea (*OR* = 4.17, 95% CI 2.04–8.53, *P <* 0.001, *I*^*2*^ = 71.3% / *OR* = 5.50, 95% CI 2.45–12.33, *P <* 0.001, *I*^*2*^ = 61%) [28, 29] and gastrointestinal symptoms (*OR* = 1.86, 95% CI 1.19–2.89, *P =* 0.006, *I*^*2*^ = 0%) [29] than non-severe group, while another systematic review and meta-analysis (2477 patients from China, Singapore, and Australia) found that there was no significant difference in the incidence of diarrhea (*OR* = 1.32, 95% CI 0.8–2.18, *Z* = 1.07, *P =* 0.28, *I*^*2*^ = 17%) or nausea and/or vomiting (*OR* = 0.96, 95% CI 0.42–2.19, *Z* = 0.10, *P =* 0.92, *I*^*2*^ = 55%) between either group. However, there was seven times higher odds of having abdominal pain in patients with severe illness when compared with non-severe patients (*OR* = 7.17, 95% CI 1.95–26.34, *Z* = 2.97, *P =* 0.003, *I*^*2*^ = 0%) [27].

TCM clinical symptoms: A systematic review and meta-analysis (484 patients from China) showed that the most common symptoms of COVID-19 patients were fever (74.0%), poor appetite (61.3%), fatigue (53.5%) and cough (50.4%). The most common tongue body, tongue coating and pulse patterns were red tongue (39.1%), greasy coating (65.3%) and deep pulse (44.4%) respectively [30].

Asymptomatic patients: A systematic review and meta-analysis (506 patients from China, Japan and USA) showed that the majority of asymptomatic patients (92.6%) remained asymptomatic during follow-up. Five patients developed symptoms, with mild fever (< 38 °C) recorded in all of them. Other symptoms such as cough, fatigue, arthralgia, dizziness, and nasal congestion were noted only in single cases [31].

Olfactory and gustatory dysfunctions: Two systematic review and meta-analysis (26,602 patients from 18 different countries) found that the overall prevalence of alteration of the sense of smell or taste was 47–52% [32, 33]. The loss of smell and taste preceded other symptoms in 20% (95% CI 13–29%) of cases and it was concomitant in 28% (95% CI 22–36%) [32]. A total of 21,515 patients were assessed in a systematic review and meta-analysis. The OR of olfactory and/or gustatory dysfunctions in COVID-19 patients were 11.26 (95% CI 5.41–23.4) when compared with acute respiratory infection without detectable virus and 6.46 (95% CI 2.79–14.97) in patients with other respiratory viruses. The OR of olfactory dysfunction in COVID-19 patients were 11.67 (95% CI 6.43–21.17) when compared with the acute respiratory infection patients without detectable virus and 4.17 (95% CI 1.34–12.98) with other respiratory viruses. The OR of gustatory dysfunction in COVID-19 patients were 12.70 (95% CI 7.9–20.44) when compared with the acute respiratory infection patients without detectable virus and 4.94 (95% CI 1.59–15.31) with other respiratory viruses. Fifty percent (95% CI 36.7–63.3%) of COVID-19 patients had olfactory and/or gustatory dysfunctions [34].

Ocular symptoms: A cross-sectional study (535 patients from China) showed that conjunctival congestion (5.0%) was one of the COVID-19-related ocular symptoms, which could occur as the initial symptoms. The other ocular symptoms, including increased conjunctival secretion (29.6%), ocular pain (18.5%), photophobia (11.1%), dry eye (37.0%) and tearing (22.2), were also found in patients with conjunctival congestion [35]. A cross-sectional study of 121 patients demonstrated that ocular symptoms including itching, redness, tearing, discharge, and foreign body sensation were among the symptoms of covid-19(5.0%) [36]. A cross-sectional study (56 patients) showed that ocular symptoms (27%) are relatively common in COVID-19 disease and may appear just before the onset of respiratory symptoms [37]. Another cross-sectional study (38 patients) found that one-third (31.6%) of patients with COVID-19 had ocular abnormalities, which frequently occurred in patients with more severe COVID-19 (66.7%) [38].

Cutaneous symptoms: A systematic review including 507 patients from China, Spain, Italy, France, USA, Canada, Belgium, Thailand, Indonesia and Japan found that the skin symptoms of COVID-19 patients were multiformity. The most common skin lesion was erythema, which was observed in 224 patients and distributed on patients’ trunk, extremities, flexural regions, face, and mucous membranes. Moreover, the erythema lesions were also confined to specific sites, such as the heels without other triggers such as exposure. Chilblain-like lesions were described in 100 (19.7%) patients. Urticaria-like lesions were presented in 83 patients (16.4%) and distributed on patients’ trunks or dispersed widely on their bodies. Two hundred twenty-seven patients (44.8%) complained of significant pruritus at the skin lesions. In addition, other manifestations such as vesicular (66, 13.0%), livedo/necrosis (31, 6.1%) and petechiae (8, 1.6%) were described. and it was noteworthy that 13 patients (14.8%) had skin lesions as the first symptom [39].

##### Justification

The evidence quality for each outcome ranged from very low to high. All this evidence focusing on clinical manifestations is crucial for the initial diagnosis of patients with COVID-19. After considering the certainty of evidence, patient preference, health equity, acceptability, feasibility, and generalizability, the guideline panel gave a strong recommendation for general clinical manifestations and weak recommendation for clinical manifestations of asymptomatic patients mostly based on low certainty of evidence. High-quality contemporaneous case-control studies are barrier to confirm that if some typical symptoms can assist clinicians to differentiate SARS-CoV-2 infection from other viral infection in people with suspicious COVID-19. However, the current included literatures were mainly systematic reviews/meta-analyses of included cross-sectional studies.

#### Question 6: Comparing with the upper respiratory tract specimens, do lower respiratory tract specimens result in better diagnostic outcomes (such as sensitivity, specificity, positive predictive value [PPV], negative predictive value [NPV], or detection rate) in people with suspicious COVID-19 when performing nucleic acid RT-PCR test?

##### Recommendations

If the patient’s condition allows (expectorating sputum spontaneously or receiving mechanical ventilation), lower respiratory tract specimens (sputum or broncho- alveolar lavage fluid) can be preferred for testing (Grade2C).

Sampling specimens from lower respiratory tract may result in a higher positive detection rate than those from upper respiratory tract specimens (Ungraded Consensus-Based Statement).

##### Implementation consideration

1. When collecting lower respiratory tract specimens, special attention should be paid to the infection protection of patients and collectors, and airborne precautions should be taken.
2. Nasal or pharyngeal swabs are preferred for patients without sputum.

##### Evidence summary

A systemic review and meta-analysis (included 757 confirmed COVID-19 patients with 3442 samples) compared different respiratory tract specimens for the detection of SARS-CoV-2 [40]. Pooled results showed that the percentage of positive samples was 43%(95% CI 34–52%; *I*^2^ = 87.04%) for oropharyngeal swabs,54% (95% CI 41–67%; *I*^2^ = 94.30%) for nasopharyngeal swabs and 71% (95% CI 61–80%; *I*^2^ = 85.12%) for sputum. According to the time of onset (0–7 days, 8–14 days and more than 14 days), sputum had the highest percentage of positive results (98, 69 and 46%) while oropharyngeal swabs had the lowest (75, 35 and 12%). The results supported sputum sampling as a primary method of COVID-19 diagnosis and monitoring, and highlight the importance of early testing after symptom onset to increase the rates of COVID-19 diagnosis. However, different target genes were used for RT-PCR detection and asymptomatic infection or mild symptom patients were not included in the systematic review and meta-analysis may reduce the credibility of the pooled results.

In addition, there were two cross-sectional studies that evaluated the positive rate of RT-PCR detection of SARS-CoV-2 in respiratory samples [41, 42]. One study including 4880 respiratory samples from suspected patients showed the positive rate of RT-PCR test were 80% (4/5) in alveolar lavage fluid, 49.12% (28/57) in sputum, and 38.25% (1843/4818) in nasal and pharyngeal swabs [42]. The positive rate of lower respiratory tract specimens (51.6%, 32/62) was higher than upper respiratory tract specimens (38.25%, 1843/4818). Another study including 8274 respiratory samples from suspected patients found the positive rate of RT-PCR test were 60% (3/5) in alveolar lavage fluid, 24.51% (25/102) in sputum, 47.92% (23/48) in oropharynx, 41.01% (2047/4992) in nasopharynx, and 20.69% (647/3127) in oropharynx combined with nasopharynx [41]. The positive rate of upper respiratory tract specimens (33.3%, 2717/8167) was higher than lower respiratory tract specimens (26.2%, 28/107). Moreover, due to the small sample size of the lower respiratory tract from suspected patients in these two cross-sectional studies, the results should be interpreted with caution.

##### Justification

Considering the controversy and uncertainty between the evidence, and because the lower respiratory tract specimen collection may bring the risk of occupational exposure, but expert opinion believed sampling specimens from lower respiratory tract may result in a higher positive detection rate, the guideline panel finally gave a weak recommendation and an ungraded consensus-based statement.

#### Question 7: Should IgM and IgG antibody tests be added on to nucleic acid RT-PCR test to have better diagnostic outcomes (i.e., sensitivity, specificity, PPV, NPV) than nucleic acid RT-PCR test alone in people with suspicious COVID-19?

##### Recommendation

Clinically diagnosed patients should be tested for SARS-CoV-2 specific IgM and IgG antibodies at 10–14 days after onset of symptoms. IgM and IgG antibodies combined test is better than using IgM or IgG antibody alone (Grade1C).

##### Implementation consideration

Clinically diagnosed patients are those with epidemiological history, typical clinical symptoms and imaging characteristics of COVID-19, but having negative RT-PCR test. It can confirm the diagnosis of COVID-19 if the SARS-CoV-2 specific IgG antibody changes from negative to positive or the IgG level in the recovery phase is more than 4 times higher than in the acute phase.

##### Evidence summary

A systemic review (included 54 studies with 15,976 samples) evaluated the diagnostic accuracy of antibody tests to detect current or past COVID-19 infection [43]. Reference standards included the RT-PCR and clinical diagnostic criteria (guidelines or combinations of clinical features). Pooled results showed that the sensitivity of IgG, IgM, and combination of IgG/IgM were 29.7% (95% CI 22.1–38.6%), 23.2% (95% CI 14.9–34.2%), and 30.1% (95% CI 21.4–40.7%) during the first week since onset of symptoms. For 8 to 14 days, the sensitivity of IgG was 66.5% (95% CI 57.9–74.2%), the sensitivity of IgM was 58.4% (95% CI 45.5–70.3%), and the sensitivity of IgG/IgM was 72.2% (95% CI 63.5–79.5%). For 15 to 21 days, the sensitivity of IgG was 88.2% (95% CI 83.5–91.8%), the sensitivity of IgM was 75.4% (95% CI 64.3–83.8%), and the sensitivity of IgG/IgM was 91.4% (95% CI 87.0–94.4%). For 22 to 35 days, the sensitivity of IgG was 80.3% (95% CI 72.4–86.4%), the sensitivity of IgM was 68.1% (95% CI 55.0–78.9%), and the sensitivity of IgG/IgM was 96.0% (95% CI 90.6–98.3%).For more than 35 days, the sensitivity of IgG was 86.7% (95% CI 79.6–91.7%), the sensitivity of IgM was 53.9% (95% CI 38.4–68.6%), and the sensitivity of IgG/IgM was 77.7% (95% CI 66.0–86.2%). Pooled specificity for all time points showed that the specificity of IgG was 99.1% (95% CI 98.3–99.6%), the specificity of IgM was 98.7% (95% CI 97.4–99.3%), and the specificity of IgG/IgM was 98.7% (95% CI 97.2–99.4%).Antibody tests may help to confirm COVID-19 infection in people who have had symptoms for more than 2 weeks and do not have a RT-PCR test, or have negative RT-PCR test results.

##### Justification

It’s important to confirm the clinically diagnosed patients. Nearly all expert evidence showed that patients should be tested for SARS-CoV-2 specific IgM and IgG antibodies no matter the results of RT-PCR. Generally speaking, for the diagnosis of infectious diseases, it is ideal if the pathogen can be directly detected from the specimen. However, due to the high conditions required for the growth of some pathogens, the long growth time and the low positive rate of detection, it is usually difficult. The detection of specific antibodies can make up for the above shortcomings to a certain extent. Based on evidence, considering health equity, acceptability, feasibility, and generalizability, the guideline panel gave a strong recommendation. Although the quality of evidence is very low, considering the rapid spread, high contagion of the virus, and urgent need for diagnosis confirmation the guideline panel gave a strong recommendation.

#### Question 8: Can chest computed tomography (CT) or x-ray be useful for diagnosing COVID-19 in suspicious people when their nucleic acid RT-PCR tests are negative? If so, which one is more useful?

##### Recommendation

Chest CT or x-ray is important alternative tests for RT-PCR test. Suspected COVID-19 patients with typical chest CT and x-ray presentation should be isolated and treated as clinically diagnosed patients (Grade1C).

##### Implementation consideration

In a low prevalence region, chest CT or x-ray should not be the primary screening or diagnosis method.

##### Evidence summary

A meta-analysis (*n =* 6218) evaluated diagnostic performance measures of chest CT [44]. For chest CT, the results of initial or repeated RT-PCR as the reference standard. The pooled sensitivity and specificity of chest CT were 94% (95% CI 91–96%; *I*^2^ = 95%) and 37% (95% CI 26–50%; *I*^2^ = 83%), respectively. In sensitivity analysis, the pooled sensitivity of chest CT for the studies with repeated RT-PCR as the reference standard was 93% (95% CI 88–96%; *I*^2^ = 87%). The pooled specificity was 35% (95% CI 23–48%; *I*^2^ = 86%). The pooled prevalence in China was 39% (95% CI 23–59%; *I*^2^ = 92%). The estimated PPV and NPV of chest CT were 1.5 and 99.8% at a disease prevalence of 1, 14.2 and 98.2% at a prevalence of 10, and 48.8% and 90.6% at a prevalence of 39%, respectively. The prevalence of COVID-19 outside China ranged from 1.0 to 22.9%. For chest CT scans, the PPV ranged from 1.5 to 30.7%, and the NPV ranged from 95.4 to 99.8%. In short, chest CT scans for the primary screening or diagnosis of COVID-19 would not be beneficial in a low prevalence region due to the substantial rate of false-positives. We downgraded this meta-analysis to very low quality for high risk of bias and inconsistency.

Besides, two diagnostic accuracy studies (*n =* 1122) met our study selection criteria [45, 46]. For chest CT and x-ray, RT-PCR was as the reference standard. The sensitivity and specificity of CT were 97.7 and 53.9% [46], the sensitivity and specificity of chest x-ray were 89.0% (95% CI 85.5–91.8%) and 60.6% (95% CI 51.6–69.2%) [45], respectively. The PPV and NPV of CT were 85.6 and 89.2% respectively [46]. The PPV and NPV of x-ray were 87.9% (95% CI 84.4–90.9%) and 63.1% (95% CI 53.9–71.7%) [45], respectively. The positive likelihood ratio (LR) and negative LR of CT were 2.12 and 0.04, respectively [46]. There was not enough available information to assess the interval time between chest CT, x-ray and RT-PCR in the two studies and thus may reduce the reliability of the evidence.

##### Justification

The evidence indicated that chest CT has high sensitivity for detecting patients with SARS-CoV-2 pneumonia but low specificity, which may lead to high false positive rate. We downgraded quality of evidence based on high risk of included studies. But some of the reasons leading to high risk of evidence was from that some of included studies were not for diagnostic performance of CT and RT-PCR, but for other research purpose, so relevant information about the diagnostic test was unclear. Facing the epidemic outbreak, suspected COVID-19 patients with typical chest CT and x-ray presentation should be diagnosed, cared and isolated as soon as possible. Although the quality of evidence is very low, considering the rapid spread, high contagion of the virus, and urgent need for early diagnosis the guideline panel gave a strong recommendation.

#### Question 9: What are the CT imaging manifestations that can assist clinicians to differentiate SARS-CoV-2 pneumonia patients from other viral pneumonia patients?

##### Recommendation

The lesions in patients with COVID-19 are mainly distributed either unilaterally or bilaterally in the lower lobes, mostly in peripheral areas. The common imaging findings for COVID-19 are as follows: ground-glass opacities (GGO), interlobular septal thickening, vascular enlargement, crazy paving pattern, subpleural bands, consolidation, and air bronchogram sign. Predominantly GGO pattern is more common than other viral pneumonias, while a mixed pattern of GGO and consolidation is less frequent than other viral pneumonias. COVID-19 pneumonia presented a higher prevalence of peripheral distribution, and involvement of upper and middle lobes than non-COVID pneumonia. Compared to moderate patients, some CT manifestations were more frequent in severe and critical type patients, such as traction bronchiectasis, interlobular septal thickening, consolidation, crazy-paving pattern, reticulation, pleural effusion, and lymphadenopathy (Grade1A).

##### Evidence summary

A systemic review and meta-analysis (included 2451 COVID-19 patients from China) regarded the chest CT manifestations of COVID-19 pneumonia in common and severe patients. In the research, the common group included moderate type patients. Severe group included severe and critical type patients. In moderate patients, pooled results indicated that the CT features of vascular enlargement were 79% (95% CI 0.74–0.84), GGOs were 78% (95% CI 0.64–0.89), subpleural bands were 58% (95% CI 0.12–0.97), and interlobular septal thickening were 51% (95% CI 0.26–0.76). Among severe patients, CT features of vascular enlargement were 93% (95% CI 0.75–1.00), GGOs were 82% (95% CI 0.68–0.92), interlobular septal thickening were 80% (95% CI 0.64–0.93), air bronchogram were 67% (95% CI 0.57–0.78), consolidation were 61% (95% CI 0.42–0.78), subpleural bands were 61% (95% CI 0.10–1.00), crazy-paving pattern were 59% (95% CI 0.42–0.79), and traction bronchiectasis were 52% (95% CI 0.30–0.73). The pooled incidences of 1 lobe affected, 2 lobes affected and over 2 lobes affected in moderate patients were 26% (95% CI 0.07–0.52), 21% (95% CI 0.01–0.54), and 57% (95% CI 0.23–0.87). The pooled incidences in severe group were 1% (95% CI 0.00–0.05), 4% (95% CI 0.00–0.10), and 94% (95% CI 0.88–0.99). The pooled incidences of unilateral pneumonia, right upper lobe involved, right middle lobe involved, right lower lobe involved, left upper lobe, left lower lobe, peripheral distribution and central distribution in moderate patients were 22% (95% CI 0.12–0.33), 49% (95% CI 0.16–0.83), 47% (95% CI 0.23–0.72), 80% (95% CI 0.74–0.86), 61% (95% CI 0.22–0.93), 81% (95% CI 0.53–0.98), 91% (95% CI 0.87–0.94), 5% (95% CI 0.00–0.24). The pooled incidences in severe patients were 5% (95% CI 0.02–0.10), 89% (95% CI 0.79–0.96), 86% (95% CI 0.76–0.94), 98% (95% CI 0.93–1.00), 92% (95% CI 0.83–0.98), 99% (95% CI 0.95–1.00), 88% (95% CI 0.62–1.00), 17% (95% CI 0.00–0.63). Compared to severe patients, moderate patients were less frequent to show the following features: traction bronchiectasis (*OR* = 0.40, 95% CI 0.24–0.67, *P =* 0.002), consolidation (*OR* = 0.31, 95% CI 0.15–0.64, *P =* 0.001), interlobular septal thickening (*OR* = 0.27, 95% CI 0.14–0.51, *P =* 0.000), crazy-paving pattern (*OR* = 0.22, 95% CI 0.11–0.44, *P =* 0.000), reticulation (*OR* = 0.20, 95% CI 0.05–0.80, *P =* 0.023), pleural effusion (*OR* = 0.19, 95% CI 0.07–0.49, *P =* 0.001), lymphadenopathy (*OR* = 0.17, 95% CI 0.07–0.41, *P =* 0.008), over 2 lobes involved (*OR* = 0.07, 95% CI 0.03–0.17, *P =* 0.000), but moderate patients were more likely to have radiographic abnormalities with 1 lobe involved (*OR* = 13.84, 95% CI 4.17–45.94, *P =* 0.000), 2 lobes involved (*OR* = 6.95, 95% CI 2.41–20.02, *P =* 0.004). For the location and distribution of lesions, moderate patients were less frequent to show abnormalities at the following locations: right upper lobe (*OR* = 0.09, 95% CI 0.04–0.21, *P =* 0.000), right middle lobe (*OR* = 0.14, 95% CI 0.06–0.29, *P =* 0.001), right lower lobe (*OR* = 0.17, 95% CI 0.05–0.56, *P =* 0.005), left upper lobe (*OR* = 0.10, 95% CI 0.04–0.25, *P =* 0.000), left lower lobe (*OR* = 0.09, 95% CI 0.02–0.38, *P =* 0.002), central distribution (*OR* = 0.18, 95% CI 0.08–0.40, *P =* 0.000), but moderate patients were more frequent to have unilateral pneumonia: (*OR* = 4.65, 95% CI 1.28–16.91, *P =* 0.020). The remaining features did not exhibit apparent association with the severity of disease: nodule (*OR* = 1.75, 95% CI 0.47–6.56, *P =* 0.093), subpleural bands (*OR* = 0.99, 95% CI 0.52–1.89, *P =* 0.983), GGOs (*OR* = 0.75, 95% CI 0.58–0.97,*P =* 0.404), vascular enlargement (*OR* = 0.51, 95% CI 0.24–1.10, *P =* 0.207), air bronchogram (*OR* = 0.16, 95% CI 0.02–1.16, *P =* 0.070), bronchial wall thickening (*OR* = 0.15, 95% CI 0.02–1.12, *P =* 0.064), peripheral distribution (*OR* = 1.17, 95% CI 0.56–2.44, *P =* 0.668) [47].

A systemic review and meta-analysis (included 52,251 COVID-19 confirmed patients from China) showed that 84% (95% CI 0.78–0.85) of COVID-19 patients had abnormal radiological findings on chest X-ray and CT scans. The radiological abnormalities of bilateral involvement were 76.8% (95% CI 0.63–0.87), consolidation were 75.5% (95% CI 0.51–0.91), GGOs were 71% (95% CI 0.4–0.9), unilateral involvement were 16.5% (95% CI 0.85–0.30) [48].

A systemic review and meta-analysis (included 934 COVID-19 patients from China, Japan and Italy, and 977 non-COVID patients from China, Japan, Australia, Italy, Brazil, South Korea, Germany, Turkey, Korea and USA) compared the chest CT findings of COVID-19 to other non-COVID viral pneumonia. Frequent CT features for both COVID-19 and non-COVID viral pneumonia were a mixed pattern of GGOs and consolidation (COVID-19, 37, 95% CI 0.17–0.56; non-COVID, 46%, 95% CI 0.35–0.58) or predominantly GGOs pattern (COVID-19, 42, 95% CI 0.28–0.55; non-COVID, 25, 95% CI 0.17–0.32), bilateral distribution (COVID-19, 81, 95% CI 0.77–0.85; non-COVID, 69, 95% CI 0.54–0.84), and involvement of lower lobes (COVID-19, 88, 95% CI 0.80–0.95; non-COVID, 61, 95% CI 0.50–0.82). COVID-19 pneumonia presented a higher prevalence of peripheral distribution (COVID-19 77, 95% CI 0.67–0.87; non-COVID, 34, 95% CI 0.18–0.49), and involvement of upper (COVID-19, 77, 95% CI 0.65–0.88; non-COVID, 18, 95% CI 0.10–0.27) and middle lobes (COVID-19, 61, 95% CI 0.47–0.76; non-COVID, 24, 95% CI 0.11–0.38) [49].

**Fig. 1:**
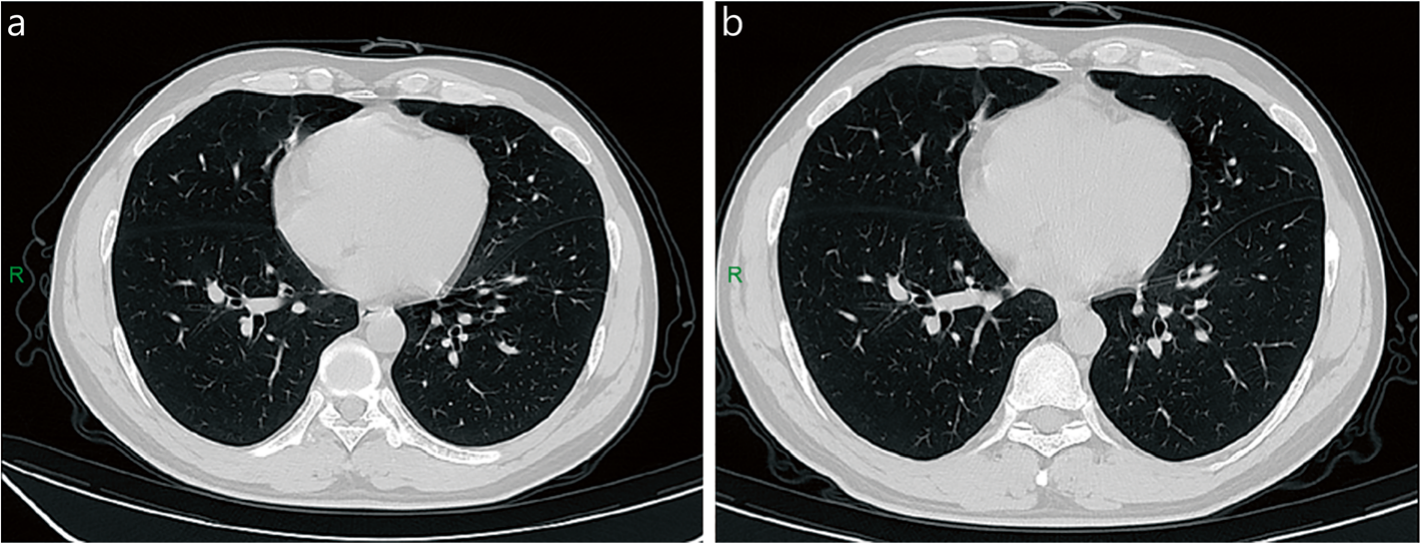
Chest CT of mild patient. A 27-year-old male patient was positive for SARS-CoV-2 after contact with COVID-19 and occasionally had a dry cough. A few old fibroses was seen in the middle lobe of the right lung, and there were no obvious changes in CT images on admission (**a**) and discharge (**b**). R: right

**Fig. 2:**
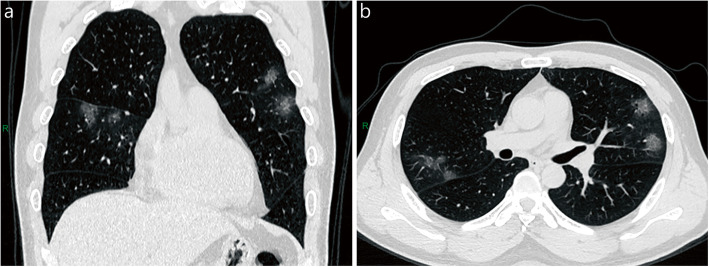
Chest CT of moderate patient. A 48-year-old male patient coughed for 1 week. Patchy ground-glass opacities were seen in the upper lobes of both lungs and the middle lobe of the right lung. Coronal (**a**) and axial (**b**) sections in lung window. R: right

**Fig. 3:**
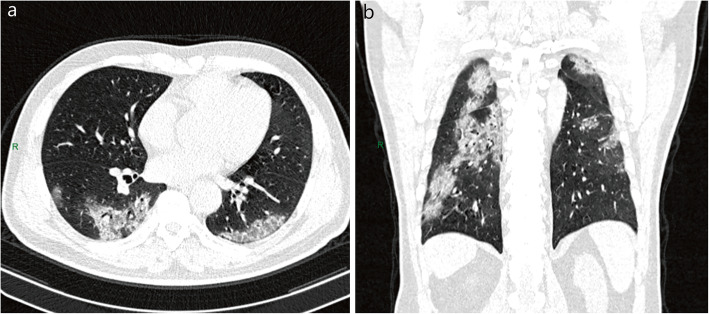
Chest CT of severe patient. A 53-year-old male patient with cough and fever for 6 days. Patchy ground-glass opacities were seen in both lungs, the central density increased, and the lesions were mainly distributed under the pleura. Axial (**a**) and coronal (**b**) sections in lung window. R: right

**Fig. 4:**
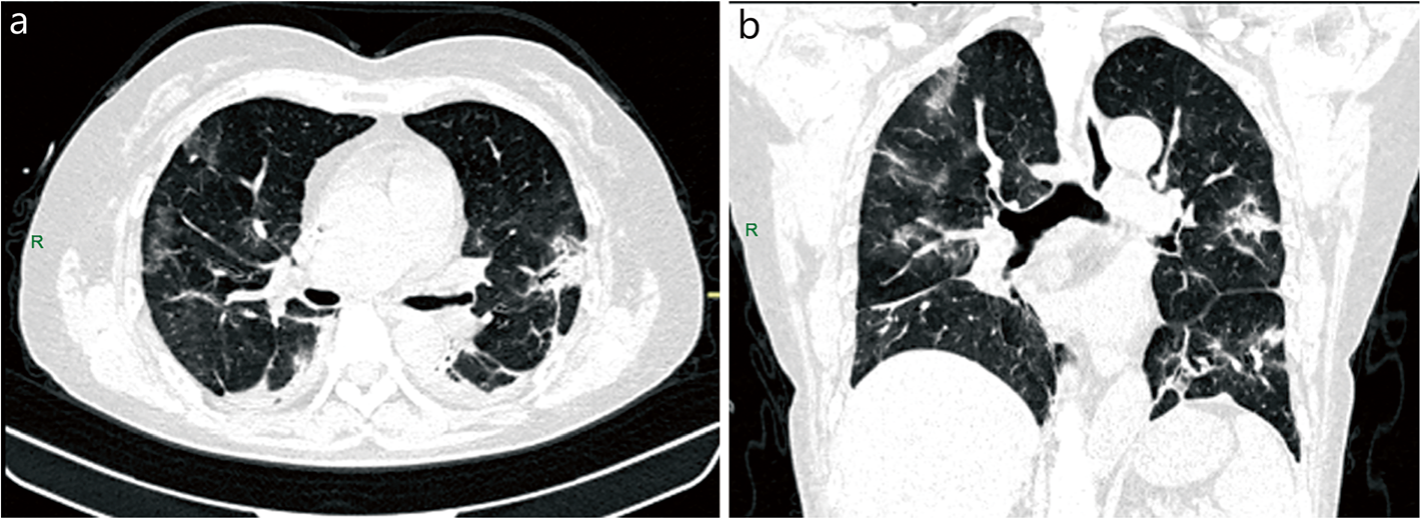
Chest CT of critical patient. A 58-year-old female patient with intermittent fever, cough and sputum for more than 1 week. Multiple patchy ground-glass opacities were seen in both lungs, and air bronchogram in the left upper lobe. Axial (**a**) and coronal (**b**) sections in lung windows. R: right

**Fig. 5:**
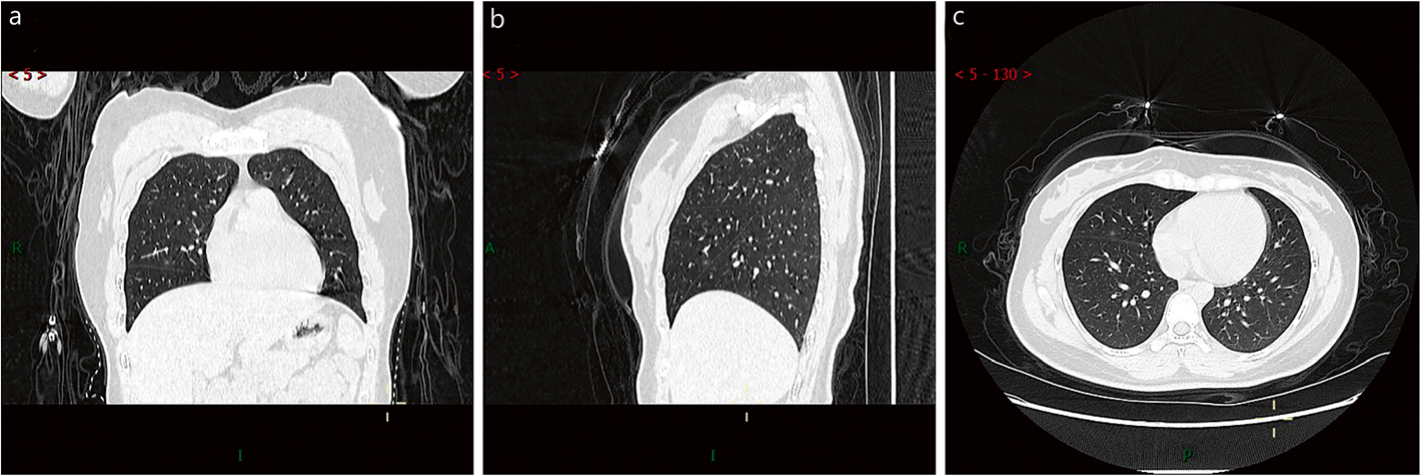
Chest CT of asymptomatic patient. A 27-year-old female with no clinical symptoms who had been in contact with COVID-19 patients was found to be positive for SARS-CoV-2 during screening. Patchy ground-glass opacities were seen in the lateral segment of the right middle lobe. Coronal (**a**), sagittal (**b**), and axial (**c**) sections in lung window. A: anterior; R: right

Figures 1, 2, 3, 4, 5*,* and 6 showed chest CT images of mild patient, moderate, severe and critical types, and asymptomatic infections with COVID-19 respectively from clinical data from Zhongnan Hospital of Wuhan University (also approved by the Committee for Ethical Affairs of this hospital).

**Fig. 6:**
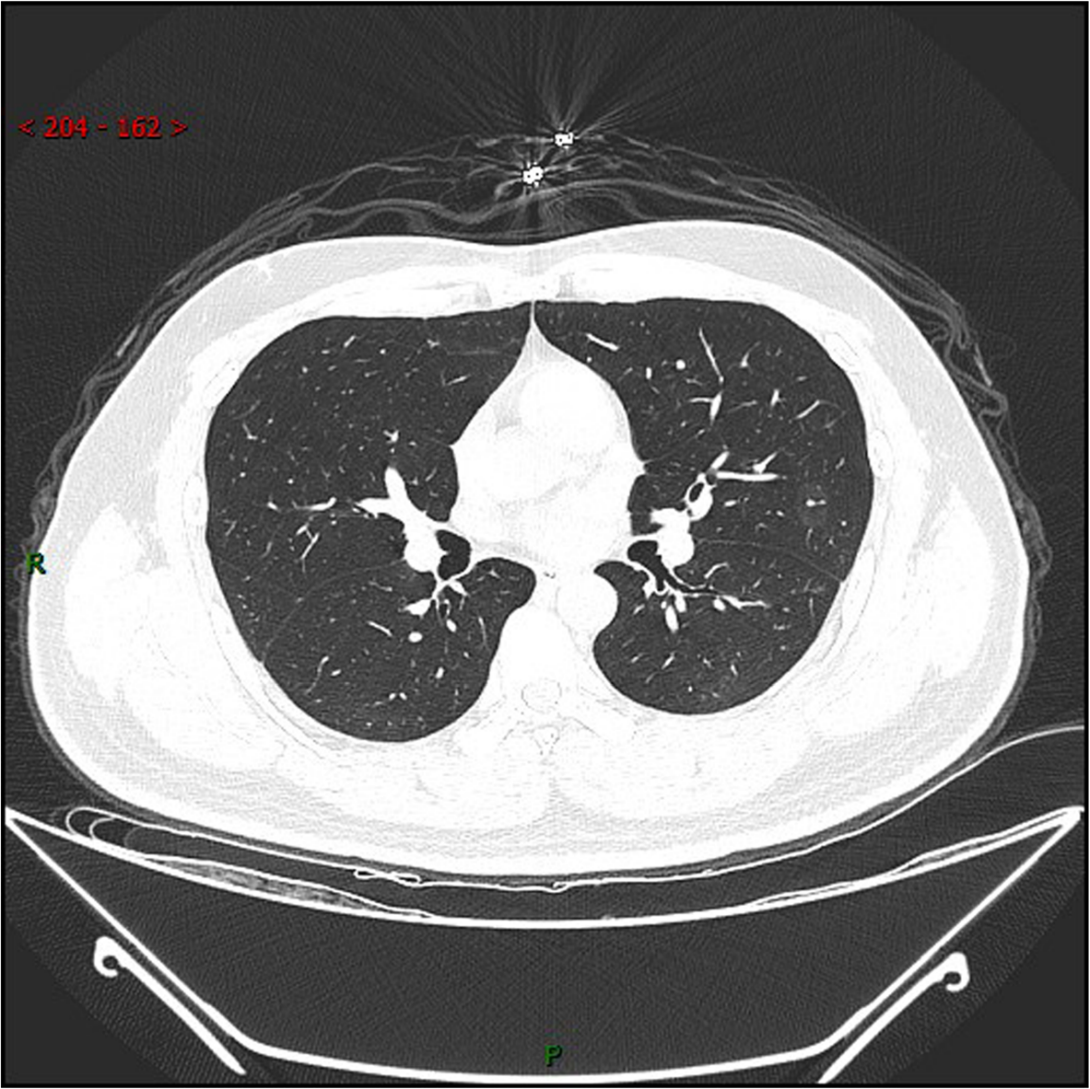
Chest CT of SARS-CoV-2 reactivation patient. A 30-year-old male patient was negative for PCR at 2 weeks’ follow-up but reverted to positive for RT-PCR at 4 weeks. The range of ground-glass opacities in the left upper lobe narrowed and the density increased slightly, while the density of ground-glass opacities in the lower right lobe decreased. R: right

##### Justification

Based on the above evidence and expert evidence, the guideline panel gave strong recommendations.

### Treatments

#### Question 10: Should lopinavir-ritonavir be used to treat patients with COVID-19 to improve clinical outcomes?

##### Recommendation

We do not suggest offering lopinavir-ritonavir to treat any type patients with COVID-19 (Grade2 (C-B)).

##### Evidence summary

One RCT [50] (*n =* 199) showed that there was no difference in the time to clinical improvement between the lopinavir-ritonavir group and standard-care group (*HR* = 1.31, 95% CI 0.95–1.85, *P =* 0.09) in patients with severe COVID-19. In terms of clinical deterioration, no difference was observed (*HR* = 1.01, 95% CI 0.76–1.34). In addition, gastrointestinal adverse events were more common in the lopinavir-ritonavir group.

The other RCT [51] randomly assigned 21 patients with mild or moderate COVID-19 to receive lopinavir-ritonavir, 16 to umifenovir, and 7 to no antiviral medication as control. The median time of positive-to-negative conversion of RT-PCR test was 8.5 (interquartile range (IQR), 3–13) days in the lopinavir-ritonavir group, 7 (IQR 3–10.5) days in the umifenovir group and 4 (IQR, 3–10.5) days in the control group, with no statistical differences (*P =* 0.75). Five (23.8%) patients in the lopinavir-ritonavir group experienced adverse events including diarrhea (14.3%), loss of appetite (9.5%) and elevation of Alanine aminotransferase (ALT) (4.8%), but no apparent adverse events occurred in the umifenovir or control group.

One non-RCT [52] reported 80 patients with COVID-19 who received lopinavir-ritonavir or favipiravir (all received interferon α2b atomized inhalation). The time of positive-to-negative conversion of RT-PCR test in lopinavir-ritonavir group (*n =* 45) was longer than that in favipiravir group (*n =* 35) (median, IQR, 11 [8–13] days vs. 4 [2.5–9] days, *P <* 0.001), but the rate of chest imaging improvement was faster in favipiravir group (91.4% vs. 62.2%, *P =* 0.004). The incidence of adverse reactions in the lopinavir-ritonavir group was higher than that in favipiravir group (55.6% vs. 11.4%, *P <* 0.001). The main adverse reactions were nausea, vomiting, diarrhea, rash, hepatic and renal injury.

A retrospective cohort study [53] investigated 108 patients given lopinavir-ritonavir and 114 given other antiviral drugs (included recombinant human interferon α1b, ribavirin injection, Lianhuaqingwen capsules). The time of positive-to-negative conversion of RT-PCR test (7.13 ± 3.36 days vs. 8.53 ± 3.85 days, *P =* 0.04) and lung imaging improvement (6 (4–8.75) days vs. 8 (5–11) days, *P =* 0.047) was shorter in lopinavir-ritonavir group than that in control group, but there was no difference in clinical symptom improvement between the two groups (*P >* 0.05). The incidence of adverse reactions in lopinavir-ritonavir group were higher than that in control group (27.8% vs. 13.2%, *P =* 0.007). The main adverse reactions included increase transaminase and bilirubin, nausea, vomiting, diarrhea, rash and so on.

One retrospective study [54] included 78 patients with COVID-19 infection with lopinavir-ritonavir and 42 without lopinavir-ritonavir (non-critical patients). The median time of positive-to-negative conversion of RT-PCR test in the lopinavir-ritonavir group was shorter than the control group (22 (IQR, 18–29) days vs. 28.5 (IQR, 19.5–38) days, *P =* 0.02) within 10 days, and did not show a significant difference > 10 days (median27.5 days vs. 28.5 days, *P =* 0.86). The study did not report adverse effects.

The other retrospective cohort study [55] recruited 42 patients with COVID-19 infection with lopinavir-ritonavir and 5 without lopinavir-ritonavir. All the patients received adjuvant drugs (included interferon aerosol inhalation and umifenovir). Although the two groups showed no significant difference (*P >* 0.05) in the body temperature of patients over 10 days, the patients in the lopinavir-ritonavir group returned to normal body temperature in a shorter time than control group (4.8 ± 1.94 days vs. 7.3 ± 1.53 days, *P =* 0.04). The time of positive-to-negative conversion of RT-PCR test in lopinavir-ritonavir group was shorter than control group (7.8 ± 3.09 days vs. 12.0 ± 0.82 days, *P =* 0.02). The study showed that compared to control group the abnormal percentage of ALT (9.5% vs. 25%) and AST (19% vs. 25%) in the lopinavir-ritonavir group was lower.

Another retrospective cohort study [56] involved 50 patients compared lopinavir-ritonavir group (*n =* 34) with umifenovir group (*n =* 16). Patients in the umifenovir group had a shorter duration of positive-to-negative conversion of RT-PCR test compared to those in lopinavir-ritonavir group (9.5(5.3–11.0) vs. 11.5(8.8–17.0), *P <* 0.01). Adverse effect: 3 patients in lopinavir-ritonavir group and 4 patients in umifenovir group showed an elevation of ALT.

The last retrospective cohort study [57] compared 52 patients with lopinavir-ritonavir, 34 with umifenovir, and 48 without antiviral medication. All the patients received interferon α2b atomized inhalation. The median time of temperature (*P* = 0.31) normalization and positive-to-negative conversion of RT-PCR test were not significantly different between the three groups (*P =* 0.79). Although the rate of adverse effect was no statistical difference between three groups, the common rate of gastrointestinal adverse reactions in lopinavir-ritonavir group, umifenovir group and control group (17.3% vs. 8.8% vs. 8.3%, respectively).

##### Justification

The two RCT studies did not find benefit from lopinavir-ritonavir group. Some cohort studies have shown benefit in lopinavir-ritonavir group, however the conventional treatment group included other antiviral drugs, which made difficult to ascertain lopinavir-ritonavir work. After balanced benefit and harms, more than 70% of working group members in the guideline panel gave a weak recommendation against using lopinavir-ritonavir. There are some ongoing trials.

#### Question 11: Should umifenovir be used to treat patients with COVID-19 to improve clinical outcomes?

##### Recommendation

Umifenovir may be considered in COVID-19 treatment (Ungraded Consensus-Based Statement).

##### Implementation considerations

1. Umifenovir 200 mg three times a day for no longer than 10 days.
2. It should be noted that some patients taking umifenovir had diarrhea and elevated serum transaminase, with occasional bradycardia.

##### Evidence summary

One RCT [51] enrolled 44 mild/moderate COVID-19 patients. The median time for positive-to-negative conversion of RT-PCR test was 8.5 (IQR, 3–13) days in lopinavir-ritonavir group (*n =* 21), 7 (IQR, 3–10.5) days in umifenovir group (*n =* 16), and 4 (IQR 3–10.5) days in control group (*n =* 7), no statistical differences (*P =* 0.75). No apparent adverse events occurred in the umifenovir or control group.

The cohort study [57] involved 134 COVID-19 patients (96% moderate cases), all received interferon α2b atomized inhalation, and 52 cases were allocated to receive lopinavir-ritonavir, 34 to umifenovir and 48 to no antiviral medication. This measured median time of temperature normalization (*P =* 0.31) and positive-to-negative conversion of RT-PCR test (*P =* 0.79) with no statistical differences between groups. Adverse effects: there were 3 cases (8.8%) with diarrhea and 2 cases with mild liver function injury in the umifenovir group, with no significant difference between groups. In addition, all the adverse reactions improved after withdrawal of drugs.

One cohort study [58] involved 49 in the umifenovir plus conventional therapy group and 62 in the conventional therapy group (defined as treatment based on clinician’s experiences and judgements). Results showed that umifenovir could accelerate and enhance the process of virus clearance (59.2% vs. 40.3%, *P =* 0.048), improve the local absorption of lung lesions (55.1% vs. 32.2%, *P =* 0.02), and reduce the demand of high flow nasal catheterization oxygen (*P =* 0.002). Adverse effects: this study showed bradycardia in one case which was alleviated after withdrawal of umifenovir.

The other retrospectively cohort study [56] involving 50 cases, compared lopinavir-ritonavir group (*n =* 34) with umifenovir group (*n =* 16). Patients in the umifenovir group had a shorter duration of positive-to-negative conversion of RT-PCR test compared to those in the lopinavir-ritonavir group (11.5(8.8–17.0) vs. 9.5(5.3–11.0), *P <* 0.01). Adverse effects: 3 patients in the lopinavir-ritonavir group and 4 patients in the umifenovir group showed an elevated ALT.

Another cohort study [59] included 62 patients with COVID-19, 42 received umifenovir combined with adjuvant therapy, and 20 received adjuvant therapy alone (included aerosol inhalation of interferon). The time of temperature normalization (4.98 ± 1.79 days vs. 6.01 ± 1.80 days, *P =* 0.02) and positive-to-negative conversion in the test group were shorter than that in the control group. While the hospitalization period in the test group was shorter, but there was no marked difference between the two groups in this aspect (16.5 ± 7.14 vs. 18.55 ± 7.52 days, *P* > 0.05). There were 7 cases (16.7%) with nausea and 2 cases (4.8%) with diarrhea and dizziness respectively in the umifenovir group, but with no significant difference between groups (*P* > 0.05).

The last retrospectively cohort study [60] included 81 moderate/severe patients with COVID-19, with 45 in the umifenovir group and 36 in the control group. Patients in the umifenovir group had a longer hospital stay than patients in the control group (13 days (IQR 9–17) vs. 11 days (IQR 9–14), *P =* 0.04). The median time of positive-to-negative conversion in the umifenovir group was longer than that in the control group (6 days (IQR 4–8) vs. 3 days (IQR 1–7) d, *P* < 0.05). As for security, 5/45 (11%) patients in the umifenovir group and 3/36 (8%) patients in the control group demonstrated digestive symptoms, including diarrhoea and nausea (*P =* 0.49), but with no significant difference between groups (*P* > 0.05).

##### Justification

The evidence was based on one RCT study and five cohort studies. The results from evidence were still inconsistent. A RCT study included three groups showed no-benefit in patients with COVID-19 used umifenovir. However, due to the imbalances in baseline characteristics of three groups and insufficient sample size, which would decrease the probability of detecting umifenovir effectiveness. In addition, most cohort studies still support its using. More than 70% of working group members in the guideline panel thought that umifenovir was a potentially effective drug based on their clinical experience although it needs confirmation from the ongoing trials.

#### Question 12: Should favipiravir be used to treat patients with COVID-19 to improve clinical outcomes?

##### Recommendation

We suggest that favipiravir can be used to treat patients with COVID-19 (Grade2B).

##### Implementation consideration

1. Favipiravir 1600 mg twice a day on day 1; then 600 mg twice a day. Treatment should generally not exceed 14 days.
2. It should be noted that the most common adverse reactions to favipiravir were digestive system reactions (nausea, acid regurgitation and flatulence), and elevated serum uric acid and ALT and/or AST.

##### Evidence summary

One RCT [61] that enrolled 236 moderate or severe COVID-19 patients with hypertension or diabetes. In moderate COVID-19 patients, favipiravir had a higher clinical recovery rate for 7 days than umifenovir (71.4% vs. 55.9%, *P =* 0.02), and led to shorter time of cough relief and fever reduction (*P <* 0.0001), but there was no statistical difference in severe patients (5.6% vs. 0%, *P =* 0.47). The most common adverse event was raised serum uric acid in the favipiravir group (13.8% vs. 2.5%, *P <* 0.01). There were also other adverse effects with no statistical differences: abnormal liver function test (elevated ALT and/or AST), psychiatric symptom reactions and digestive tract reactions (nausea, anti-acid, flatulence) between two groups.

A non-RCT [52] reported 80 patients with COVID-19, 35 with favipiravir, and 45 with lopinavir-ritonavir, all the patients also received interferon α2b atomized inhalation. The time of positive-to-negative conversion of RT-PCR test in favipiravir group was lower than that in lopinavir-ritonavir group (median, IQR, 4 (2.5–9) days vs. 11 (8–13) days, *P <* 0.001), and chest imaging improvement rate was significantly faster compared to lopinavir-ritonavir group (91.4% vs. 62.2%, *P =* 0.004). The incidence of adverse reactions in favipiravir group was lower than that in the control group (11.4% vs. 55.6%, *P <* 0.001). The main adverse reactions were nausea, vomiting, diarrhea, rash, hepatic and renal injury, and so on.

##### Justification

The evidence from a RCT and a non-RCT, the quality of the studies were medium risk because of the lack of allocation concealment, blind method and unadjusted confounding bias, which would affect uncertainty of evidence. In addition, the included research samples are all from China leading to uncertain whether they are suitable for other countries. We downgraded quality of evidence based on risk of bias, imprecision and indirectness. After balancing benefit and harms, more than 70% of working group members believed that favipiravir may have benefit for certain patients and voted a weak recommendation. There are still relevant trials in progress.

#### Question 13: Should interferon be used to treat patients with COVID-19 to improve clinical outcomes?

##### Recommendation

Interferon may be considered in COVID-19 treatment (Ungraded Consensus-Based Statement).

##### Implementation consideration

INF-α (5million U or equivalent), 2 ml sterile water for injection, twice a day, atomized inhalation. The treatment should generally not exceed 14 days. In addition, the use of interferon in different countries can be carried out according to the corresponding drug instructions.

##### Evidence summary

One open-label randomized clinical trial [62] enrolled 81 patients with COVID-19, 42 received interferon β-1a (12 million IU/ml of interferon β-1a was subcutaneously injected three times weekly for two consecutive weeks), 39 received only the standard of care (included other antiviral drugs). Compared with the control group, the IFN group had significantly increased discharge rate on day 14 (66.7% vs. 43.6%, *OR* = 2.5, 95% CI1.05–6.37) and decreased 28-day mortality (19% vs. 43.6%, *P =* 0.015). In addition, early administration significantly reduced mortality (OR = 13.5, 95% CI 1.5–118). There was no difference in the time of clinical improvement (9.7 ± 5.8 vs. 8.3 ± 4.9 days, *P =* 0.95) and duration of mechanical ventilation (10.86 ± 5.38 vs. 7.82 ± 7.84, *P =* 0.47) between two groups. The rate of adverse effects was not different between the groups. But injection-related side effects still happened in 8 (19%) in IFN group.

A cohort study [63] enrolled 256 patients with COVID-19. One hundred six patients in interferon β1b group (subcutaneous injection at a dose of 250 μg on alternate days, for moderate-severe pneumonia, with a duration between 3 and 5 doses) and 150 patients in control group. All patients received conventional treatment (included other antiviral drugs). The study showed that the interferon β1b group was not associated to decrease in -hospital mortality (20.8% vs. 27.3%, *P =* 0.229). The study did not report any of adverse effects.

One prospective cohort study [64] enrolled 814 patients with COVID-19 in Cuba. Seven hundred sixty-one were treated with the IFN-α2b (intramuscular injection, 3 million IU 3 times per week, for 2 weeks) combined with the approved protocol (included lopinavir-ritonavir and chloroquine), 53 received the protocol without IFN treatment. The rate of discharged patients was higher in the IFN-treated compared with non-IFN treated group (95.4% vs. 26.1%, *P <* 0.01). The IFN group had significantly decreased mortality (0.9% vs. 32.1%, *P <* 0.01). The study did not report any of adverse effects.

The other retrospective cohort study [65] involved 77 moderate patients with COVID-19, 7 were treated with nebulized IFN-α2b, 24 with umifenovir, 46 with combined treatment of IFN-α2b plus umifenovir. The study showed that the time of positive-to-negative conversion of RT-PCR test using IFN-α2b was shorter than that in umifenovir group (*P =* 0.003). The study did not report any of adverse effects [65].

##### Justification

Insufficient evidence for a graded recommendation. The current evidence came from one RCT study and three cohort studies. The results from evidence were inconsistent, only one cohort study showed no-benefit used interferonβ1b to patients with COVID-19. While the study selection and unmeasured confounding bias cannot be completely excluded. In addition, the conventional treatment group included other antiviral drugs, which would affect uncertainty about their effects. More than 70% of working group members believed that benefits outweigh risk of INF using, so we gave “Ungraded Consensus-Based Statement”.

#### Question 14: Should remdesivir be used to treat COVID-19 patients to improve clinical outcomes?

##### Recommendation

We suggest that remdesivir can be used to treat patients with COVID-19 (Grade2(C-B)).

##### Implementation considerations

1. Remdesivir 200 mg loading dose on day 1, followed by 100 mg daily for no longer than 10 days, intravenously.
2. The most common adverse reactions to remdesivir were anemia or decreased hemoglobin.

##### Evidence summary

A systematic review and meta-analysis [66] included 5 studies (3 RCTs and 2 case series) patients with COVID-19. 2 RCTs evaluated 10-day treatment of remdesivir efficacy versus placebo group and one RCT compared its 5-day regimen versus 10-day regimen. The meta-analysis revealed that10-day treatment regimen overpowered 5-day treatment and placebo in decreasing time to clinical improvement (MD = -3.02, 95% CI -4.98 ~ − 1.07, *P =* 0.002). Although there was no significantly difference between remdesivir group and placebo group in reducing the rate of mortality (OR = 0.72, 95% CI 0.39–1.36, *P =* 0.32), remdesivir group overpowered placebo in decreasing time to clinical improvement (MD = -3.02, 95% CI -4.98 ~ − 1.07, *P =* 0.002). All adverse event rates did not have significant difference; however, severe adverse event rate was lower remdesivir group compared to placebo group (OR = 0.71, 95% CI 0.55–0.92, *P =* 0.009), especially in 5-day (OR = 1.98, 95% CI1.27–3.11, *P =* 0.003). One case series included in this systematic review analyzed the available data of 53 patients, 34 needed invasive oxygen support and the other 19 needed non-invasive oxygen support. Mortality rate was higher in invasive group (18% vs. 5%). Participants in invasive group experienced more adverse events (65% vs. 53%). The other case series of 35 patients included in this systematic review, 9 of 18 ICU patients and 13 of 17 ward patients completed the 10-day course of remdesivir therapy. ICU patients had lower clinical improvement rate (38.9% vs. 88.2%) by day 28. The most common severe adverse events observed were elevation of liver enzymes (42.8%) and acute kidney injury (22.8%).

##### Justification

There are only 2 studies comparing the efficacy of remdesivir group and placebo group which included in the meta-analysis above, and the results of these two studies were controversial. The report of a RCT in China demonstrates no benefit in clinical outcomes in using remdesivir for treatment of severe patients with COVID-19. However, the inability to recruit the predetermined study population resulted in study power reduction from 80 to 58%. Low study power and higher severity of illness in remdesivir group both decreases the probability of detecting remdesivir effectiveness. The large RCT study included 1059 patients (88.7% were severe) with COVID-19 has shown benefit in the time to recovery, but it did not have a statistically significant effect on deaths. In addition, remdesivir is also in short supply and is complex to administer (it must be given by injection over the course of several days). Those evidence were low risk of bias, and we downgraded quality of evidence just because of inconsistency. This systematic review and mate analysis included case series which we thought they were no benefit for adding more evidence for remdesivir based on that we already had RCTs with low risk of bias and case series has a very low ability to demonstrate causality. Although the effect of remdesivir on survival remains unknown, more than 70% of working group members believed that remdesiviris potentially effective in some ways, and its benefits outweigh risk of using remdesivir. Results of some ongoing RCTs may provide strong evidence for this treatment option.

#### Question 15: Could a combination of antiviral drugs be used to treat patients with COVID-19 to improve clinical outcomes?

##### Recommendations

There is insufficient evidence to for or against using combination of antiviral drugs (Grade2C)**.**

Three or more antiviral drugs should not be used at the same time (Ungraded Consensus-Based Statement).

##### Evidence summary

One open-label, randomised, phase 2 trial study [67] recruited 127 patients with COVID-19. 86 were randomly assigned to the combination group (included lopinavir-ritonavir, ribavirin and interferon beta-1b) and 41 were assigned to the control group (given lopinavir-ritonavir). All patients received conventional treatment. The study showed that the combination group had a shorter time in negative conversion of SARS-CoV-2 within 7 days (6.5 d (IQR 4–8) vs. 12.5d (8–14.8), *P <* 0.0010), clinical improvement within 7 days (4 d (3–5) vs. 8 d (6.5–9), *P <* 0.0010) and duration of hospital stay within 7 days (8 d (6.0–12.5) vs. 15 d (9–16.0), *P =* 0.003) than the control group in mild/moderate/severe patients with COVID-19. There was no significant difference in the rate of adverse reactions (48% vs. 49%) between two groups. No serious adverse events were reported in the combination group. No patients died during the study.

A non-RCT study [68] included 237 patients with COVID-19. One hundred ninety-six patients were received oral umifenovir, lopinavir-ritonavir and interferon α2b in the combined group and 41 were received lopinavir-ritonavir and interferon α2b in control group. All patients received conventional treatment. The study show that the combined group had a shorter time in negative conversion of SARS-CoV-2 (12.2 ± 4.7 d vs. 15.0 ± 5.0 d, *P <* 0.01) and median length of hospital stay (12 d vs. 15 d, *P <* 0.05) than control group in patients with COVID-19. There was no difference in the rate of ARDS between two groups (11.7% vs. 19.5%, *P <* 0.05).

A cohort study (pre-print) [69] included 73 patients with COVID-19. Thirty-four patients were treated with lopinavir-ritonavir, 39 with lopinavir-ritonavir plus umifenovir. All patients received conventional treatment. The study showed that treatment with lopinavir-ritonavir alone was not difference from lopinavir-ritonavir combined with umifenovir in negative conversion rate of SARS-CoV-2 (92.3% vs. 97.1%, *P =* 0.618), in negative conversion time of SARS-CoV-2 (11.5 ± 9.0 d vs. 9.9 ± 7.5 d, *P =* 0.585), in the rate of severe disease progression (5.1% vs. 0%, *P =* 0.495), in the rate of chest CT imaging improvement (84.6% vs. 91.1%, *P =* 0.489), in the length of hospital stay (14.4 ± 7.9 d vs. 16.0 ± 9.0 d, *P =* 0.431) and in the rate of mortality (2.6% vs. 2.9%, *P >* 0.99) for moderate and severe patients with COVID-19. The study did not report adverse effects.

One cohort study [70] included 33 patients with COVID-19. Sixteen patients were received oral umifenovir and lopinavir-ritonavir in the combined group and 17 were received oral lopinavir-ritonavir only in the monotherapy group. All patients received conventional treatment. The study show that combined group had a higher rate in negative conversion of SARS-CoV-2 at 7 days (75% vs. 35%, *P <* 0.05) and rate of chest CT imaging improvement after 7 days (69% vs. 29%, *P <* 0.05) than the lopinavir-ritonavir group in patients with COVID-19. The study did not report adverse effects.

The other cohort study [71] involving 141 patients with COVID-19. Combined group patients were given Umifenovir and IFN-α2b (*n =* 71), monotherapy group patients inhaled IFN-α2b (*n =* 70). All patients received conventional treatment. The study show that the combined group had a faster time in chest CT imaging improvement (16.7 vs. 19.8 d, *P =* 0.037), but there were no difference in time of negative conversion of SARS-CoV-2 (27.4 d vs. 23.8 d, *P =* 0.057) and hospital stay (24.2 d vs. 27.1 d, *P =* 0.056) between two groups. There were no differences between the two groups in ALT, Aspartate aminotransterase (AST), or creatinine during or after treatment. But 13 patients (18.8%) in combined group demonstrated mild nausea, stomachache, and all patients could tolerate without giving up treatment.

The last cohort study [72] involving 109 non-critical patients with COVID-19, 58 received interferon α and 51 received interferon α combine lopinavir-ritonavir. All patients received conventional treatment. Patients in the combined group had a higher rate of clinical improvement than interferon α group at 7 days (70.6% vs. 48.3%, *P <* 0.05). Although the median time of positive-to-negative conversion in the combined group was shorter than that in the interferon α group, with no difference between two groups (16.43 vs. 21.79, *P >* 0.05). The combined group was higher than interferon α group in the rate of adverse effects (80.4% vs. 27.4%, *P <* 0.05). Although all the adverse reactions were treated with symptomatic treatment or the symptoms were improved after drug withdrawal.

##### Justification

The current evidence from one RCT study, one non-RCT study and 4 cohort studies. The evidence for most comparisons was moderate because of risk of confounding (lack of appropriate statistical analysis) and the limited number of participants. The results from evidence were inconsistent, one RCT study and two cohort studies still support early administration. In addition, due to the lack of no-treatment group, the studies can only show that the combined group was better than the monotherapy group, but can not be extrapolated to the combined group was better than the no-treatment group. Based on the risk of bias and inconsistency of evidence, and inconclusive result of any antiviral drug alone, we did not draw any recommendation for combination of antiviral drugs. All experts believed that three or more antiviral drugs should not be used at the same time.

#### Question 16: Should hydroxychloroquine (HCQ)/ chloroquine (CQ) be used to treat patients with COVID-19to improve clinical outcomes?

##### Recommendations

There is inconsistent evidence to for or against using HCQ/CQ in COVID-19 treatment (Grade2C).

We do not suggest using the combination of HCQ and azithromycin (AZ) (Grade2C).

##### Evidence summary

A systematic review and meta-analysis (*n =* 10,659) showed that HCQ cannot effectively reduce mortality (8 observational studies, RR = 0.98, 95% CI 0.66–1.46), or clinical deterioration of ARDS (6 observational studies, RR = 0.90, 95% CI 0.47–1.71). There was no statistically significant difference in virologic clearance (2 RCTs and 3 observational studies, RR = 1.03, 95% CI 0.83–1.28) and in time to fever remission (2 RCTs and 1 observational study, WMD = − 0.54 days, 95% CI -1.19-0.11) between HCQ and placebo. Compared with standard-of-care (SOC), HCQ increases the risk of ECG abnormalities/cardiac arrhythmias with or without azithromycin (2 observational studies, RR = 1.46, 95% CI 1.04–2.06). Two RCTs related to virologic clearance were all open labels. Most of the comparative studies were of poor methodologic quality and were subject to high risk of bias owing to the non-randomized study design and the lack of placebo control [73]. A living systematic review came to a conclusion that evidence on the benefits and harms of using HCQ or CQ is very weak and conflicting. Among the 4 RCTs included, 2 RCTs have a high risk of bias in selection of the reported result, and 2 RCTs have some concerns on the randomization process or selection of the reported result [74].

A multicenter, randomized, parallel, open-label, trial evaluated 150 (mild/moderate or severe) COVID-19 patients, 75 patients were assigned to HCQ (loading dose of 1200 mg daily for 3 days followed by a maintenance dose of 800 mg daily for the remaining days) plus SOC and 75 were assigned to SOC alone. Results showed that the positive-negative conversion rate of RT-PCR test at day 28 was similar for the two groups (85.4, 95% CI 73.8–93.8%) vs. (81.3, 95% CI 71.2–89.6%, *P =* 0.34). Significant efficacy of HCQ in alleviating symptoms was observed when the confounding effects of anti-viral agents were removed in the post-hoc analysis (HR = 8.83, 95% CI 1.09–71.3). Twenty-one adverse events were reported in HCQ patients, 1 with disease progression and 1 with upper respiratory tract infection, the others were non-serious adverse events, such as diarrhea and vomiting, which were significantly higher than those reported in the SOC group (*P =* 0.001) [75].

A RCT was performed in Brazil to assess safety and efficacy of two different chloroquine diphosphate (CQ) dosages (high dose CQ: 41 patients, 600 mg CQ twice daily for 10 days or total dose 12 g; low dose CQ: 40 patients, 450 mg, twice daily only on the first day then daily for 5 days, total dose 2.7 g). Of the 81 cases, 61 cases were confirmed by RT-PCR, and 19 cases were unconfirmed cases but had clinical and epidemiological presentation. All patients received AZ. One patient developed rhabdomyolysis, which was attributed to CQ, and the drug was withdrawn. QTc interval corrected by the Fridericia method (QTcF) ≥ 500 ms was more frequent in the high-dosage group than the low-dosage group (18.9% vs. 11.1%). Two of 37 patients (2.7%) in the high-dosage group experienced ventricular tachycardia before death, without torsade de pointes. Hemoglobin decrease was observed in both groups (high-dosage vs. low-dosage: 19.2% vs. 22.2% decrease respectively). Raised creatinine was observed in both groups (high-dosage vs. low-dosage: 39.1% vs. 46.7% increase respectively). No apparent differences in hematological or renal toxicity were seen between the groups. Mortality was 39.0% in the high-dosage group and 15.0% in the low-dosage group with no apparent differences despite more deaths in the high-dosage group [76].

A cohort study from the US evaluated 807 COVID-19 patients (HCQ, *n =* 198, the median age (IQR) was 71 (62–76.8) years; HCQ + AZ, *n =* 214, the median age (IQR) was 68 (59–74) years; no HCQ, *n =* 395, the median age (IQR) was 70 (59–77) years). Rates of ventilation in the HCQ, HCQ + AZ, and no HCQ groups were 19.0, 20.5, 19.9%, respectively, *P =* 0.94. Compared to the no HCQ group, the risk of death from any cause was higher in the HCQ group (adjusted HR = 1.83, 95% CI 1.16–2.89, *P =* 0.009) but not in the HCQ + AZ group (adjusted HR = 1.31, 95% CI 0.80–2.15, *P =* 0.28). The propensity-score-adjusted risk of mechanical ventilation was similar in the HCQ group (adjusted HR = 1.19, 95% CI 0.78–1.82, *P =* 0.42) and in the HCQ + AZ group (adjusted HR = 1.09; 95% CI 0.72–1.66, *P =* 0.69), compared to the no HCQ group [77].

##### Justification

More than 70% of working group members believed that there was inconsistent data to for or against using HCQ based on the above evidence and its quality, and clinicians’ own experience. However, in different contexts, different countries can make their own consensus statements. For example, China made the consensus recommendation on CQ on March 42,020. There is also insufficient evidence to support the combination of HCQ and AZ leading to better clinical outcomes than HCQ alone, but we also know both of these drugs may cause Q-T prolongation. Hence, we do not recommend this combination at present. However, antibiotics therapy should be prescribed for patients having concurrent bacterial infection.

To date, at least 71 clinical trials of HCQ/CQ for COVID-19 have been registered. When new evidence that may change the current recommendation is available, we will update the recommendation.

#### Question 17: Should interleukin-6 inhibitors be used to treat COVID-19 patients to improve clinical outcomes?

##### Recommendation

There is insufficient evidence to support or against using interleukin-6 inhibitors (Grade2C).

##### Evidence summary

A meta-analysis of 3641 patients including 16 studies (13 retrospective cohort studies and 3 prospective cohort studies) showed that adding tocilizumab (TCZ) to standard of care (SOC) may reduce the mortality of severe COVID-19 (Pooled *OR* = 0.57, 95% CI 0.36–0.92, *P =* 0.02), and it did not report any adverse effect. However, this evidence body was a low-quality evidence with degrading factors: more confounding factors (the difference in the age and comorbidities, variability in the follow-up period) and significant heterogeneity (*I*^*2*^ = 80%) among the included studies [78].

The following studies were not included in the above meta-analysis:

A non-randomized controlled study (29 vs. 24) showed that after adjusting for age and mechanical ventilation, use of TCZ (400 mg, iv., two doses) was not associated with mortality of COVID-19 patients in ICU (*OR* = 3.97, 95% CI 0.28–57.2, *P =* 0.3), and no adverse events were reported that could be directly related to TCZ [79]. A propensity-score matched cohort study (74 vs. 148 severe/critical patients) found TCZ use was associated with a better overall survival (*HR* = 0.499, 95% CI 0.262–0.952, *P =* 0.035), but the length of hospital stay with TCZ was longer (dose: 8 mg/kg, *HR =* 1.658, 95% CI 1.088–2.524, *P =* 0.019). Besides, infectious complications were observed in 32.4% of TCZ group, and 14.9% of TCZ patients were accompanied by severe events (sepsis cases, candidemia, lung abscess or epidural abscess) [80]. Another propensity-score matched cohort study (84 vs. 84 severe patients; 400 mg single-dose) came to similar conclusions in improving overall survival (adjusted *HR* = 0.26, 95% CI 0.135–0.51, *P =* 0.0001), and it did not report any adverse effect [81]. A cohort study found TCZ therapy (dose: 8 mg/kg) in hyperglycaemic (*n =* 31) failed to attenuate risk of severe outcomes as it did in normoglycaemic patients (*n =* 47) (*P <* 0.009), and it did not report any adverse effect [82].

A small sample of open-label cohort study (28 vs. 28) showed that overall clinical improvement, mortality, and the rate of adverse events (infections, neutropenia, increase in liver enzymes and thromboembolism) in severe COVID-19 patients were not significantly different between sarilumab and SOC at 28 days of follow-up (all *P* > 0.05). In addition, sarilumab (400 mg, iv.) was associated with faster recovery in a subset of patients showing minor lung consolidation at baseline (*P =* 0.002) [83]. Another propensity-score matched cohort study (30 vs. 30) showed that the 30-day mortality rate in patients with COVID-19 respiratory failure was significantly lower in the siltuximab (11 mg/kg, iv.) than in the control (*HR* = 0.462, 95% CI 0.221–0.965, *P =* 0.0399), and no adverse events were reported to be related to the study drug [84].

Since most of the evidence listed were retrospective cohort studies with fewer samples, they usually had more confounding factors, such as age, gender, disease severity, and comorbidities. Although most studies used methods/models to control measurable confounding, confounding factors still existed. The overall quality was medium or low, and no upgrade factors were found.

##### Justification

Although meta-analysis as high-quality evidence has shown that tocilizumab can reduce mortality, its methodological quality is not high, so its strength of evidence needs to be downgraded. Tocilizumab is a representative of Interleukin-6 inhibitors, increasing evidence has shown that tocilizumab could decrease the mortality of COVID-19 patients, but due to the limitations of study type (mainly observational research) and small samples, high-quality studies are still needed to verify the effectiveness of tocilizumab.

#### Question 18: Should interleukin-1 inhibitors be used to treat COVID-19 patients to improve clinical outcomes?

##### Recommendation

There is insufficient evidence to support or against using interleukin-1 inhibitors (Grade2C).

##### Evidence summary

A cohort study (52 vs. 44) showed that severe COVID-19 patients who were treated with anakinra, administered subcutaneously at a dose of 100 mg twice daily for 3 days, then 100 mg daily for 7 days had a significant reduction on the need for invasive mechanical ventilation or death in the multivariate analysis (*HR* = 0.22, 95% CI 0.10–0.49, *P =* 0.0002). Besides, the frequency of elevated liver enzymes, coagulopathy was similar between patients in anakinra and control, and it is unlikely that anakinra might be caused [85].

A cohort study (29 vs. 16) showed that moderate-severe COVID-19 patients who were treated with anakinra, administered subcutaneously at a high dose of5 mg/kg twice a day intravenously had a higher survival (90% vs. 56%, *P =* 0.009). Besides, the incidence of bacteremia, increased liver enzymes, and thromboembolism was similar in the two groups [86].

Since the evidence listed were retrospective cohort studies with fewer samples, they usually had more confounding factors. Although most studies used methods/models to control measurable confounding, confounding factors still existed. The overall quality was medium or low, and no upgrade factors were found.

##### Justification

To date, there is insufficient evidence to recommend for or against to use interleukin-1 inhibitors in COVID-19 patients. Additionally, working group members had no clinical experience of using Interleukin-1 inhibitors.

#### Question 19: Should glucocorticoid be used to treat COVID-19 patients to improve clinical outcomes?

##### Recommendations

We do not suggest to use glucocorticoid for patients with COVID-19 in general (Grade 2B).

When sever or critical COVID-19 patients’ condition deteriorates dramatically, low-dose glucocorticoid with a short course may be considered (Grade 2B).

##### Implementation considerations

1. Methylprednisolone (MP) can be considered to be used as a low dose of 1–2 mg/kg/day for a short course of about 3 days;
2. Dexamethasone can be considered to be added as a dose of 6 mg once daily (oral or intravenous) for up to 10 days.

##### Evidence summary

A systematic review (including 11 retrospective studies, *n =* 4168 patients; 1 RCT, *n =* 6425 patients) showed that a common pattern evolving from the retrospective trials suggested more benefit with low dose steroids compared to the high dose steroids. Moreover, judicious use of corticosteroids had been shown to improve several parameters of severe and critical COVID-19, including reduction of duration of hospital stay, prevention of worsening of the ventilator parameters, progression to ARDS, and death, quicker normalization of pyrexia and improvement in the status of oxygenation, reduced incidence of intubation and subsequent ventilation, but the results from these retrospective studies were heterogenous and difficult to infer of a definitive protective benefit with corticosteroids. RECOVERY trial (multicenter RCT conducted in 176 NHS hospitals, *n =* 6425 patients, 2104 for dexamethasone-6 mg once daily for up to 10 days and 4321 for usual care) found dexamethasone reduced 28-day mortality by 35% amongst the invasive mechanical ventilation patients (29.0% vs. 40.7%, *RR* = 0.65, 95% CI 0.51–0.82, *P <* 0.001) and by 20% amongst patients on supplemental oxygen therapy with or without noninvasive ventilation (21.5% vs. 25.0%, *RR* = 0.80, 95% CI 0.70–0.92, *P =* 0.002), although no benefit was observed in mild cases (17.0% vs. 13.2%, *RR* = 1.22, 95% CI 0.93–1.61, *P =* 0.14). It did not report any adverse effect [87]. In this SR, most of included studies had a small cohort size and had a high degree of heterogeneity regarding the choice of steroids, the dose and timing of the steroids, and had a co-prescription of broad-spectrum antibiotics and antivirals. However, this included multi-center, large-sample RCT clearly confirmed that the effectiveness of glucocorticoid therapy in reducing mortality, especially for severe patients.

The following studies were not included in the above systematic review:

A retrospective cohort (*n =* 115 patients, 73 for glucocorticoid group, 1-3 mg/kg per day for 3-10 days and 42 for control group) found that compared with conventional treatment, corticosteroid treatment was associated with a 2.155-fold increase in risk of either mortality or ICU admission in multivariate analysis (adjust for disease severity), although not statistically significant, and the corticosteroid group had more adverse outcomes (32.9% vs. 11.9%, *P =* 0.013) [88].

Another retrospective cohort (*n =* 72 patients, 51 for glucocorticoid group: 0.75–1.50 mg/kg/d and 21 for control group) found that there was no significant difference between two groups in the median time from the onset to the negative detection of nucleic acid in sputum (*P >* 0.05), and it would cause some adverse reactions, such as transient hyperglycemia, hypokalemia, acne like skin rash and high blood pressure [89].

A retrospective cohort study based on propensity score analysis (*n =* 132 non-severe COVID-19 patients, matching 35 for corticosteroid group-initial MP dosage 40 mg/d for 8–12 days, and 35 for control group) found that in corticosteroid group, the hospital stay and duration of viral shedding were prolonged, while fever time was shortened, however all these data had no statistically significant differences, and it did not report any adverse effect [90].

A multicentric, partially randomized, preference, open-label trial (*n =* 85 COVID-19 patients, 56 for MP and 29 for control) showed that a short course of MP had a beneficial effect on the clinical outcome of severe COVID-19, decreasing the risk of the composite end point of admission to ICU, NIV or death (*RR =* 0.55, 95% CI 0.33–0.91, *P =* 0.024). No major side effects were observed, but hyperglycemia was more frequent in the MP group [91].

A retrospective cohort (*n =* 202 non-ICU patients, 60 for corticosteroid group, and 145 for control group) found that patients who received corticosteroids were less likely to have had a primary outcome (composite of ICU transfer, intubation or death) than were patients who did not receive corticosteroids (adjusted *HR* = 0.15; 95% CI 0.07–0.33, *P <* 0.001), and it did not report any adverse effect [92].

A retrospective cohort study (*n =* 463 patients, 396 for steroids and 67 for control) showed that survival of COVID-19 patients was higher in glucocorticoids group than control (*HR =* 0.51, 95% CI 0.27–0.96, *P =* 0.044), especially among with moderate or severe ARDS (*OR* = 0.23, 95% CI 0.08–0.71, *P =* 0.014). In-hospital mortality was not different between initial regimens of 1 mg/kg/day of MP and steroids pulses (*OR* = 0.880, 95% CI 0.449–1.726, *P =* 0.710), and it did not report any adverse effect [93].

A multicenter, observational, longitudinal study (*n =* 173 severe COVID-19 patients, 83 for MP and 90 for control) showed that early administration of prolonged MP treatment was associated with a significantly lower hazard of death (adjusted *HR* = 0.29, 95% CI 0.12–0.73,*P =* 0.005) and decreased ventilator dependence (24.0 ± 9.0 days vs. 17.5 ± 12.8 days; *P =* 0.001). The complication rate was similar for the two groups (*P =* 0.84) [94].

A retrospective cohort (*n =* 72 patients, 56 for tocilizumab+ MP group, and 16 for tocilizumab group) found that MP administered in patients treated with tocilizumab reduces the risk of death (*RR* = 0.20, 95% CI 0.08–0.47, *P <* 0.01), and it did not report any adverse effect [95].

Since most of the evidence listed were retrospective cohort studies with fewer samples, they usually had more confounding factors, such as age, gender, disease severity, and comorbidities. Although most studies used methods/models to control measurable confounding, confounding factors still existed. The overall quality was medium or low, and no upgrade factors were found.

##### Justification

Although the results from retrospective studies are heterogeneous and difficult to infer a definitive protective benefit with corticosteroids, RECOVERY trial, as one of the world’s largest RCT for COVID-19, found a significantly better outcome with dexamethasone, mostly in severe cases. Besides, dexamethasone and methylprednisolone are easily available in pharmacies, cost less, and have better economic benefits. In addition, there were limited drug-related adverse reactions during short-term use. After considering the desirable and undesirable effects, balancing the benefits and harms and based on their clinical opinion, more than 70% of working group members thought low-dose glucocorticoid may be considered for severe or critical patients when their condition deteriorates dramatically.

#### Question 20: Should QingfeiPaidu decoction (TCM) be used to treat patients with COVID-19 to improve clinical outcomes?

##### Recommendation

*QingfeiPaidu Decoction (QPD)* may be considered to treat patients with mild or moderate COVID-19 (Ungraded Consensus-Based Statement).

##### Implementation considerations

1. Constituent parts: Ephedrae Herba 9 g, Glycyrrhizae Radix Et Rhizoma Praeparata Cum Melle 6 g, Armeniacae Semen Amarum 9 g, Gypsum Fibrosum 15-30 g (Decocted earlier), Cinnamomi Ramulus 9 g, Alismatis Rhizoma 9 g, Polyporus 9 g, Atractylodis Macrocephalae Rhizoma 9 g, Poria 15 g, Bupleuri Radix 16 g, Scutellariae Radix 6 g, Pinelliae Rhizoma Praeparatumcum Zingibere Et Alumine 9 g, Zingiberis RhizomaRecens 9 g, Asteris Radix Et Rhizoma 9 g, Farfarae Flos 9 g, Belamcandae Rhizoma 9 g, Asari Radix Et Rhizoma 6 g, Dioscoreae Rhizoma 12 g, Aurantii Fructus Immaturus 6 g, Citri Reticulatae Pericarpium 6 g, Pogostemonis Herba 9 g.
2. QPD, water decoction, 200 ml twice a day, 40 min after meal, warm-taken, 3 days a course, can be taken up to four courses based on patients’ clinical manifestations.

##### Evidence summary

A cohort study showed that compared with antiviral treatment (oseltamivir, abidor, lopinavir/ritonavir) (30 patients), the hospital stay duration was shortened (13.633 ± 0.398 vs.16.433 ± 0.295 days, *P* < 0.05) after being treated by QPD plus antiviral drugs (30 patients), the antipyretic time (2.346 ± 0.852 vs. 3.852 ± 0.774 days, *P* < 0.05) and the improvement time of lung CT images (6.571 ± 0.497 vs. 8.800 ± 0.395 days, *P* < 0.05) was both significantly shortened. There were no significant differences of the disease condition worsening (20.0% vs. 40.0%, *P* > 0.05) and cure rate (90.0% vs. 83.3%, *P* > 0.05). Fewer cases of adverse reactions appeared in the experimental group (1 cases [nausea] vs. 3 cases [2 cases of nausea and 1 case of diarrhea]) [96].

##### Justification

The available evidence is very weak, but after balancing benefit and harms, considering patient preference, acceptability, feasibility, and more than 70% of working group members thought QPD may be a treatment option for patients with COVID-19, based on their clinical opinion. The results of three ongoing trials will provide evidence for this treatment option.

But considering lacking of generalizability in some countries for TCM treatment and lacking of confident evidence, we finalized recommendation with “ungraded Consensus-Based Statement”.

#### Question 21: Should Lianhua Qingwen granules/capsules (TCM) be used to treat patients with COVID-19 to improve clinical outcomes?

##### Recommendation

We suggest that Lianhua Qingwen can be used to treat patients with mild or moderate COVID-19 with conventional therapy (defined as nutritional supportive therapy, symptomatic treatment, antiviral and antibacterial treatment if needed) (Grade2C).

##### Implementation considerations

Lianhua Qingwen Granules/Capsules: 6 g/1.4 g by mouth, three times per day for 14 days.

##### Evidence summary

One RCT of mild patients showed that, compared with arbidol treatment (148 patients), the TCM syndrome scores (based on the TCM syndrome rating scale) were significantly decreased (*P* < 0.05) after 7 days treatment with Lianhua Qingwen Granules (LQG) plus arbidol (147 patients),the total effective rate (excellent effective rate + effective rate) was increased (81.0% vs. 64.9%, *P* < 0.05), and lung CT images showed improvement (69.4% vs. 62.8%, *P* > 0.05) in the experimental group, no serious adverse reactions appeared in each group [97].

Another RCT showed that, compared with routine treatment (oxygen therapy, antiviral medications and symptomatic therapies) (142 patients), after 14 days treatment with LQG plus routine treatment(142 patients), the recovery rate was significantly higher (91.5% vs. 82.4%, *P* < 0.05),the median time to symptom recovery was markedly shorter (7 vs. 10 days, *P* < 0.001),time to recovery of fever was also significantly shorter (2 vs. 3 days, *P* < 0.001),the rate of improvement on lung CT images (83.8% vs. 64.1%, *P* < 0.001) and clinical cure (78.9% vs. 66.2%, *P* < 0.05) was higher in treatment group. However, the rate of conversion to severe cases or viral assay findings had no significant difference in both groups (*P* > 0.05). No serious adverse events appeared in each group [98].

One non-RCT reported that comparing with conventional therapy (nutritional supportive therapy, symptomatic therapy, antiviral therapy, and antibacterial therapy) (51 moderate patients), LQG plus conventional therapy (51 moderate patients) resulted in a higher rate of fever resolved (83.7% vs. 61.0%, *P* < 0.05) after 7 days treatment, less rate of change to severe types of COVID-19 (7.84% vs. 21.57%, *P* < 0.05), and higher rate of improvement on lung CT images (54.9% vs. 45.1%, *P* > 0.05) [99].

Another non-RCT showed that, compared with conventional therapy (nutritional supportive therapy, symptomatic therapy, antiviral therapy, and antibacterial therapy) (21 moderate patients), the fever better resolved (85.7% vs. 57.1%, *P* < 0.05) after being treated by LQG plus conventional therapy (21 moderate cases) and the fever duration shortened (4.6 ± 3.2 days vs. 6.1 ± 3.1 days, *P* > 0.05) [100].

The third non-RCT reported compared with conventional treatment (nutritional supportive therapy, symptomatic treatment, antiviral and antibacterial treatment) (38 suspected cases), the fever better resolved (86.7% vs. 67.7%, *P* < 0.05) and the disease condition less worsened (6.4% vs. 15.8%, *P* > 0.05) after being treated by LQG plus conventional therapy (63 suspected cases) for 10 days and showed no adverse reactions [101].

Among other four studies, important confounding information existed, the overall risk was judged as moderate or serious.

##### Justification

After balancing benefit and harms, and considering the quality of evidence, patient preference, acceptability, and feasibility, the guideline panel gave a weak recommendation for Lianhua Qingwen Granules/Capsules to treat COVID-19 with conventional therapy.

#### Question 22: Should convalescent plasma be used to treat COVID-19 patients to improve clinical outcomes?

##### Recommendation

There is insufficient evidence to for or against using convalescent plasma to treat severe and critical COVID-19 patients (Grade2B).

##### Evidence summary

A Cochrane’s systematic review [102], which retrieved until June 4, 2020, explored the effectiveness of convalescent plasma for COVID-19 patients. Control groups received SOC. Results from 1 non-randomized studies of interventions (NRSIs) with 21 participants (6 received convalescent plasma) showed that convalescent plasma has no effect on all-cause mortality at hospital discharge (RR = 0.89, 95% CI 0.61–1.31, *P =* 0.56). Results from 1 RCT (103 participants, of whom 52 received convalescent plasma) and 1 NRSI (195 participants, of whom 39 received convalescent plasma) showed that convalescent plasma may not prolongs time to death (RCT: HR = 0.74, 95% CI0.30–1.82; NRSI: HR = 0.46, 95% CI 0.22–0.96), and may has no effect on improvement of clinical symptoms at 7 days (RCT: RR = 0.98, 95% CI 0.30–3.19), 14 days (RCT: RR = 1.85, 95% CI, 0.91–3.77; NRSI: RR = 1.08, 95% CI 0.91–1.29), and 28 days (RCT: RR = 1.20, 95% CI 0.80–1.81). This systematic review included results from 1 RCT, 3 controlled NRSIs and 10 non-controlled NRSIs assessing safety of convalescent plasma. Thirteen studies (201 participants) reported on adverse events of possible grade 3 or 4 severity. The majority of these adverse events were allergic or respiratory events. A non-controlled NRSI (5000 participants), which reported only on serious adverse events limited to the first 4 h after convalescent plasma transfusion. This study reported 15 deaths, four of which they classified as potentially, probably or definitely related to transfusion. Almost all included studies revealed a significant risk of bias, due to study design, type of participants, and other previous or concurrent treatments. The included RCT were unblinded for participants and personnel, selection of the reported result, and have bias in incomplete outcome data.

An RCT [103] in the Netherlands was halted prematurely after 86 patients were enrolled. Patients were randomly assigned via a web-based system to the convalescent plasma group (*n =* 43) and SOC group (*n =* 43). Results showed that convalescent plasma has no effect on overall mortality (OR = 0.95, 95% CI 0.20–4.67, *P =* 0.95) and was not associated with a shorter time to discharge from the hospital (HR = 0.88, 95% CI 0.49–1.60, *P =* 0.68). No plasma related serious adverse events were observed.

Another RCT [104] (49 participants, of whom 21 received convalescent plasma) showed that convalescent plasma reduced duration of infection about 4 days (19.3 ± 6.9 days vs.23.42 ± 6.4 days, *P <* 0.05), and showed less death rate (1/21 vs. 8/28, *P <* 0.05).

##### Justification

There is insufficient evidence to for or against using convalescent plasma. Most of studies have shown no benefit, but the quality of evidence is low. China made the consensus recommendation on convalescent plasma for severe and critical cases. In different contexts, different countries can make their own consensus statements. Plasma components are complex, and there may be risks associated with infusion, such as allergy and the spread of infectious diseases. Therefore, the whole process of recovery, plasma collection, preparation, storage, inspection, and application must conform to quality assurance systems and comply with pharmaceutical production quality management specifications. But there was insufficient data to support or against using convalescent plasma. Some trials involving convalescent plasma for COVID-19 are ongoing.

#### Question 23: Should lung transplantation be used to treat patients with COVID-19 to improve clinical outcomes?

##### Recommendation

Lung transplantation maybe a therapeutic option for end-stage patients with COVID-19 (Ungraded Consensus-Based Statement).

##### Implementation consideration

Firstly, three critical points should be thoroughly evaluated and confirmed before decision-making regarding lung transplantation candidacy: 1) confirmed irreversibility of refractory respiratory failure despite maximal medical support [105]; 2) confirmed positive-turned-negative virology status by performing consecutive nucleic acid tests with samples derived from multiple sites [105, 106]; and 3) confirmed absence of other organ system dysfunction that could contraindicate lung transplantation [105]*.*

Secondly, best practices for the protection of the medical team involved are as follows: 1) head covers with positive pressure are necessary for surgeons, nurses, anesthesiologists, and cardiopulmonary physicians; 2) head covers will help surgeons keep their field of view clear without fogging of eye protectors; 3) considering the physical demands and challenges for surgeons in full protective clothing, an intra-procedure rotation plan is necessary to guarantee optimal performance during surgery [105].

In addition, multiple disciplinary teams (intensive care unit, respiratory, infectious, and radiology departments) are necessary to minimize the possibility of misjudgments whether the lung injury in COVID-19 patients is irreversible [106].

##### Evidence summary

Two case series reported that five patients received antiviral, hormonal, convalescent plasma, and immune-enhancing supportive treatments and life supporting extracorporeal membrane oxygenation (ECMO), but their condition continued to worsen. After lung transplantation, the vital signs of four patients with end-stage COVID-19 pneumonia were stable, the chest X-ray showed the transplant lungs were clear, and the ECMO was removed successfully [105, 106]. However, the right lung of another patient was transplanted uneventfully. During the left lung transplant procedure, ventricular fibrillation developed abruptly and the heart arrested. Cardiac massage was commenced and cardiopulmonary bypass was established with cannulation via the superior, inferior venae cava and ascending aorta. Emergent heart transplant was also performed. The heart was resuscitated to normal rhythm with strength. But bleeding from the chest cavity and anastomosis could not be managed with sutures and coagulation in the following 5 h. The transplanted heart arrested again, and the patient was pronounced dead [105].

In addition, one case report stated that a COVID-19 patient was treated with high-flow nasal oxygen, methylprednisolone, umifenovir, piperacillin, and tazobactam. And then although repeated nucleic acid tests for 2019-nCoV in sputum and bronchoalveolar lavage fluid were all negative, his condition continued to deteriorate due to pulmonary consolidation complicated by stenotrophomonas maltophilia infection. And then he continued to get ECMO treatment and a bilateral-lung transplantation. Postoperatively, the ECMO was withdrawn and the patient’s general condition was more stable. However, ST-segment elevation myocardial infarction after lung transplantation occurred. He received percutaneous coronary intervention. Post percutaneous coronary intervention ECG showed recovery of ST-segment, and cardiac troponin I gradually declined [107].

##### Justification

In general, the panel did not include case reports or case series as evidence to make recommendations for intervention research question. However, lung transplantation is a very complicated treatment procedure and it is impossible to expect to have a RCT to investigate whether lung transplantation is effective. Based on evidence, five of six survived from dying status, the panel believed that lung transplantation may be a treatment option for dying COVID-19 patients without other treatment options if it is possible.

#### Question 24: What are the indications for the use of invasive or noninvasive ventilation?

##### Recommendation

For patients with high-flow nasal oxygen (HFNO) or non-invasive ventilation (NIV) showing no improvement or worsening of their condition or oxygenation index ≤150 mmHg within a short period of time (1–2 h), endotracheal intubation and invasive mechanical ventilation should be performed promptly (Grade 1C).

##### Implementation considerations

1. Closely monitor patients’ general conditions, vital signs, respiratory status, especially changes in oxygenation index.
2. Choose HFNO or NIV when nasal cannula or mask oxygen therapy is ineffective or patients have hypoxic respiratory failure.
3. For invasive mechanical ventilation, ARDS lung protective ventilation strategy should be adopted: low tidal volume (4–6 ml/kg) and low plateau pressure (< 30 cmH_2_O), appropriate positive end expiratory pressure (PEEP.) For patients with moderate to severe ARDS (oxygenation index: PaO_2_/FiO_2_ < 150 mmHg), use a higher PEEP and perform prone ventilation for more than 12 h a day along with deep sedative analgesia in the first 48 h of mechanical ventilation. For patients with severe acute hypoxic respiratory failure, attention should be paid to prevention of ventilator-related lung injury following mechanical ventilation.

##### Evidence summary

Expert evidence suggested that when respiratory distress and/or hypoxemia could not be relieved after giving standard oxygen therapy, HFNO therapy or NIV could be considered. If the condition did not improve or worsen within a short time (1–2 h), tracheal intubation and invasive mechanical ventilation should be performed as soon as possible. Or, in adults with COVID-19 and acute hypoxemic respiratory failure on oxygen, it is recommended that SpO_2_ be maintained no higher than 96%. In adults with COVID-19 and acute hypoxemic respiratory failure, experts suggested using HFNO over conventional oxygen therapy or NIV, and recommended close monitoring for worsening of respiratory status, and early intubation in a controlled setting if worsening occurs**.**

##### Justification

There are no clinical studies to answer this research question. Nearly all of working group members believed that mechanical ventilation should be recommended as a rescue treatment for no improvement or worsening with HFNO and NIV in severe or critical COVID-19 after balancing the benefits and harms. However, different countries may have slightly different the indications for the use of invasive or noninvasive ventilation.

#### Question 25: What are the indications for use of Extracorporeal membrane oxygenation (ECMO)?

##### Recommendation

ECMO is recommended to treat patients with critical COVID-19, and close monitoring of patient’s vital signs is necessary during use. ECMO should be used in the following situations: 1) early stage (such as severe type with a course of less than 7 days) of critical patients with reversible condition; 2) severe hypoxemia: when using optimized PEEP, PaO_2_/FiO_2_ < 100 mmHg after using neuromuscular blocker and prone ventilation; 3) excessive compensatory respiratory acidosis (pH < 7.15) when using optimized mechanical ventilation; 4) excessive inspiratory stress (plateau pressure > 30 cmH_2_O) when using lung protective ventilation; 5) using optimized mechanical ventilation setting, the mechanical power is ≥27 J/min; 6) using the optimized mechanical ventilation setting, there is right heart dysfunction due to acute pulmonary heart disease (Grade 1C).

##### Implementation considerations

1. Using ECMO when patients are in the early stages of critical COVID-19 is crucial.
2. Multiple teams and departments should collaborate to provide refined management of COVID-19 patients.

##### Evidence summary

Expert evidence suggested that ECMO should be considered as soon as possible for patients with severe ARDS and poor ventilation in the prone position. The indications are: 1) When FiO_2_ > 90%, the oxygenation index is less than 80 mmHg for more than 3–4 h; 2) Airway plateau pressure ≥ 35 cmH_2_O.

##### Justification

Nearly all of working group members agreed with the above indications for use of ECMO after balancing the benefits and harms. However, different countries may have slightly different indications for use of ECMO based on their context.

### Discharge management

#### Question 26: What are the discharge criteria for COVID-19 patients?

##### Recommendation

Patients meeting all the following criteria can be discharged: 1) temperature returned to normal for more than 3 days; 2) respiratory symptoms significantly improved; 3) significant absorption of pulmonary chest lesions on CT imaging; 4) two consecutive negative nucleic acid tests from sputum, nasopharyngeal swabs or other respiratory tract samples (at least 24 h between samples) (Ungraded Consensus-Based Statement).

##### Implementation and considerations

Meanwhile, we need to consider patient’s age, combidity, clinical type of COVID-19, and other factors (such as hospital capacity) to decide whether we need to add stool nucleic acid testing and/or serological testing as a part of discharge criteria.

##### Justification

Although there was no direct evidence, the working group members believed that the discharge criteria from expert opinion was reasonable and had achieved good results in China. However, whether it is needed to add stool nucleic acid testing and/or serological testing as a part of discharge criteria is unclear. Different countries may make slightly different discharge criteria based on their context.

#### Question 27: What are the imaging findings in COVID-19 patients whose RT-PCR test is positive for COVID-19 after previously recovering?

##### Recommendation

Most of people have no progressive imaging findings in chest CT of COVID-19 patients whose RT-PCR test shows positive after previously recovering

##### Implementation considerations

Chest CT should be performed in recovered patients from COVID-19 whose RT-PCR test showed positive after discharge.

##### Evidence summary

There are seven studies, including 279 patients whose RT-PCR shows positive recovery from COVID-19. All the patients received chest CT imaging, with (29.4–90.2%) cases showing improvement, and (8–32%) cases showing no active progression. The chest CT of one case presented recurrent symptoms with blurred image in the upper lobe of both lungs, more prominent on the left side during the convalescent period, but the severity of image is less than that of late period of hospitalization [108–114].

##### Justification

According to the above low-quality evidence, the working group members thought although most patients have no progressive imaging changes was found, confirmation by a larger sample study is needed in the future.

Figure 6 showed chest CT images of SARS-CoV-2 reactivation patient from clinical data from Zhongnan Hospital of Wuhan University (also approved by the Committee for Ethical Affairs of this hospital).

#### Question 28: What is the management plan in patients whose RT-PCR retesting shows SARS-CoV-2 positive after discharge?

##### Recommendation

After the first discharge, if the RT-PCR test reverts from negative to positive, the patients should be isolated again and may be re-hospitalized based on their clinical characteristics. The effective treatments should be given as early as possible if needed. If the lung image does not have progressive change comparing with that at the first discharge, and patients have three negative RT-PCR tests from sputum and fecal specimens (each≥24 h apart), the patients can be managed according to the requirements of home isolation and follow-up again (Ungraded Consensus-Based Statement).

##### Implementation considerations

A combination of sputum and fecal specimen types (at least one of the three negative RT-PCR tests should coming from a fecal test) should be used to detect the nucleic acid of SARS-CoV-2 for the retested positive patients after discharge, i.e., at least one of the three negative RT-PCR tests should coming from a fecal test.

##### Evidence summary

One cross-sectional study found that viral RNA could also be detected in the feces of 81.8% (54/66) patients with COVID-19 (after discharge 6–11 days) when pharyngeal swabs were negative. Fecal specimens test should be more useful than nasopharyngeal swab [115].

A cohort study reported that 3% (23/651) patients had positive RT-PCR testing again during the follow-up period. Among the retested positive patients, 12 patients (52%) had moderate, 9 patients had (39%) severe, and 2 patients had (9%) critical conditions during their previous hospitalization. 50% of the patients carried IgG antibodies and 30% of the patients carried IgM antibodies suggested partial immune system recognition of SARS-CoV-2.The detection of IgG and IgM antibodies should be increased on the basis of RT-PCR for retested positive patients. And it also reported that the median duration from hospital discharge to positive retest was 15 days [116].

A cross-sectional study found that 15.9% (11 / 69) of patients had positive RT-PCR testing again after discharge and the median interval from discharge to positive RT-PCR results again was 14 days, 10 of the 11 patients had mild or moderate infection and only 1 patient had critical infection, which suggest that strict self-isolation protocols and extended follow-up periods might be needed for recovered COVID-19 patients [117].

Another cross-sectional study from China reported that 14.5% (25/172) of patients had positive RT-PCR testing again after discharge 5–13 days, so discharge criteria should be reevaluated or reset [114].

A case report from China found that some discharged patients’ condition aggravated again after discontinuation of antiviral drugs, which may be one of the reasons for recovered patients with COVID-19 testing positive again. It is suggested that not only consider the patient’s viral nucleic acid test results, but also the manifestations on chest computed tomography to determine whether patients can stop taking antiviral drugs [118].

##### Justification

In order to strengthen epidemic prevention and control, based on the eligible limited evidence and clinical experience, more than 70% of working group members agreed that the number of RT-PCR tests for these patients should be increased from two to three comparing with those at the first hospital discharge. Because all discharged patients followed a strict protocol for self-isolation, which believe that the RNA positivity at follow-up is unlikely to be due to reinfection.

Different countries may make different management plan in patients whose RT-PCR retesting shows SARS-CoV-2 positive after discharge. More high-quality clinical research is needed to confirm this statement. We did not find any trial to verify the management strategies for patients whose RT-PCR retesting shows SARS-CoV-2 positive after discharge, so we gave recommendation on “Ungraded Consensus-Based Statement”.

#### Question 29: Is the RT-PCR retesting needed to monitor COVID-19 patient after discharge?

##### Recommendation

Discharged patients may be quarantined for 2 weeks, with follow-up, and PCR tests can be performed at 2 and 4 weeks after discharge (Ungraded Consensus-Based Statement).

##### Implementation consideration

Home quarantine is the primary choice for patients after discharge. If there is a designated centralized isolation area, patients may receive medical observation in this area.

##### Justification

The evidence was same as question of “management plan in patients whose RT-PCR retesting shows SARS-CoV-2 positive after discharge”. The possibility of patients becoming RT-PCR positive again after discharge raises the potential risk of transmission. Thus, surveillance of discharged patients is needed. More than 70% experts reached agreement. Different countries may make different surveillance plan for discharged patients based on their context.

## Guideline implementation tools

**Fig. 7:**
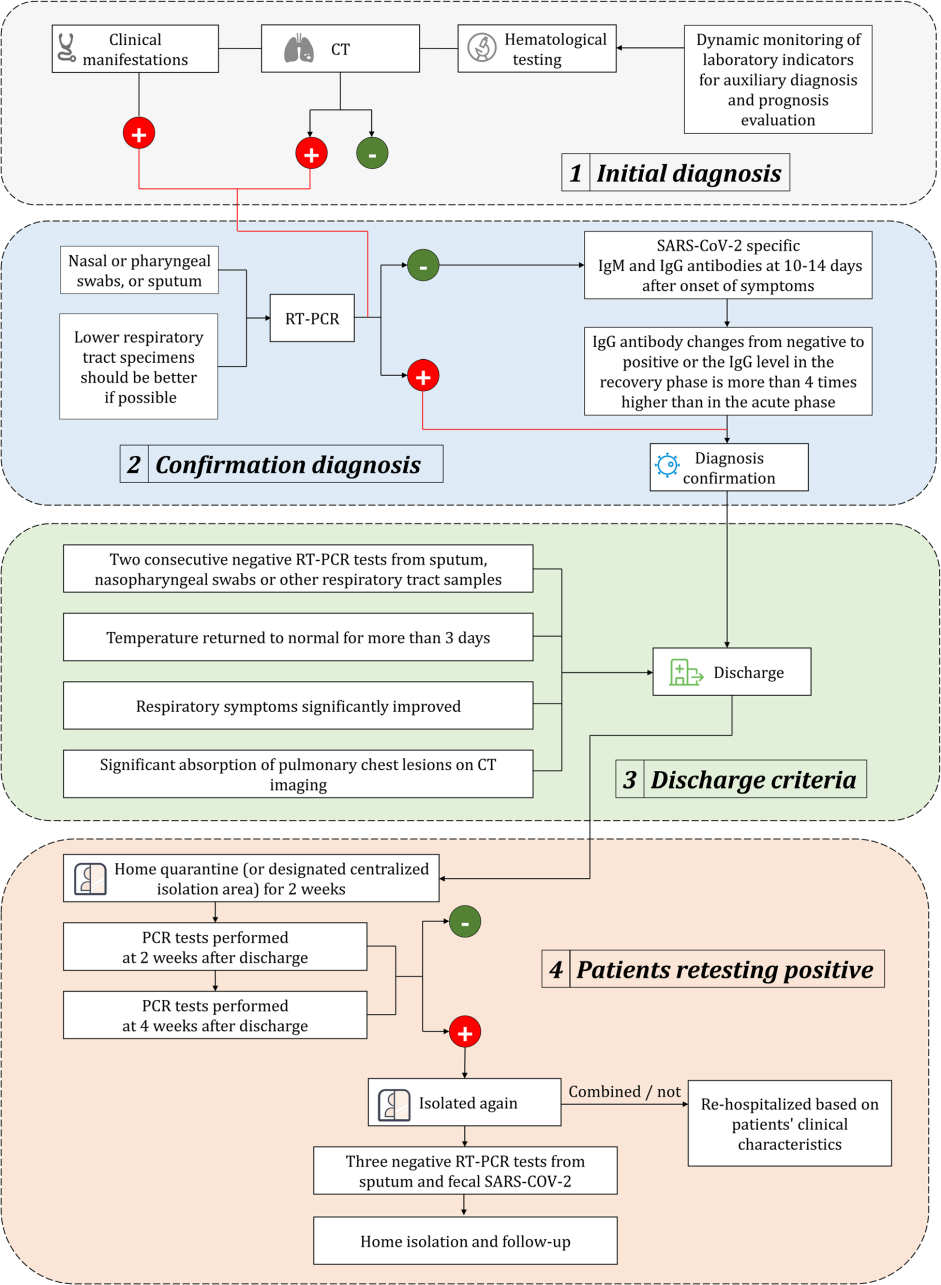
Implementation tool for diagnosis section and discharge management section

**Fig. 8:**
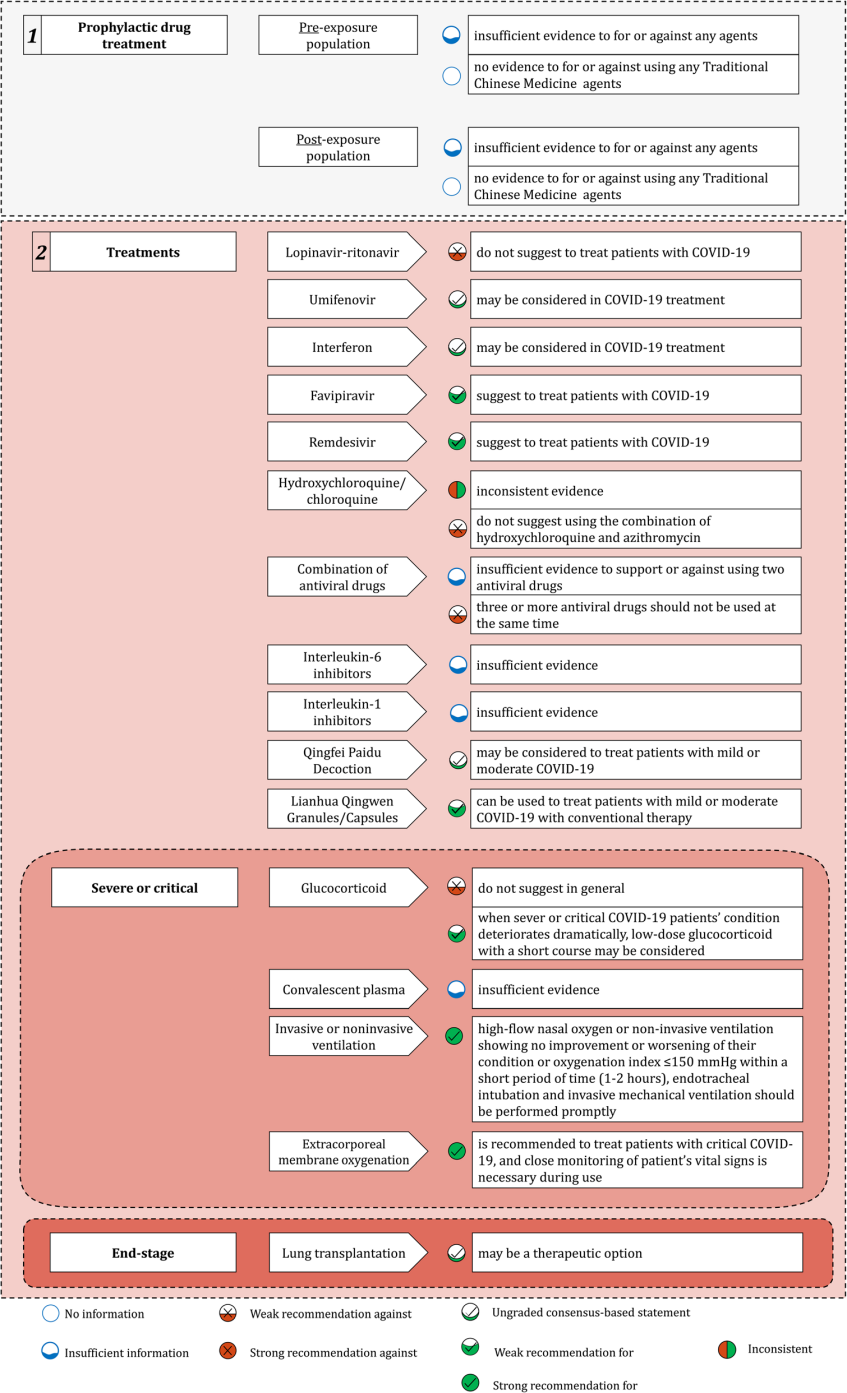
Implementation tool for chemoprophylaxis and treatments section

We created Fig. 7 for chemoprophylaxis and treatments sections, and Fig. 8 for diagnosis, and discharge management sections respectively for the implementation purpose.

## Discussion

Our recommendations, based on the best available evidence, can timely provide references to the world-wide clinicians regarding on preventive drug treatments, diagnosis, treatment and discharge management on patients with COVID-19.

We got the recommendation of “Chest CT or x-ray is important alternative tests for RT-PCR test. Suspected COVID-19 patients with typical chest CT or x-ray presentation should be isolated and treated as clinically diagnosed patients”. In the worldwide, we can see a nucleic acid test has currently accepted as the gold standard method to confirm diagnosis. In addition, imaging examination and epidemiological history were usually considered as auxiliary diagnosis methods. Although the use of radiological evidence to confirm viral pneumonia may be an important alternative to the diagnosis and monitoring of COVID-19, it also brought some problems. This procedure may include some patients with common pneumonia; hence criteria for clinically diagnosed patients also need to include the nucleic acid results at a later stage to correct the actual number of cases [119, 120].

Classes of drugs are being evaluated or developed for the management of COVID-19 for months, and more than one thousand trials were conducting in the whole world. Most antiviral drugs undergoing clinical testing in patients with COVID-19 are repurposed antiviral agents originally developed against influenza, HIV, Ebola, or SARS/MERS. Unfortunately, we have no high confidence for any one treatment. Although we gave weak recommendation for using remdesivir, the effect of remdesivir on survival remains unknown.

A human vaccine is currently not available for SARS-CoV-2, but nearly 120 candidates are under development. A randomised, double-blind, placebo controlled, phase 2 trial and a preliminary report of a phase 1/2, single-blind, randomised controlled trial have published recently, and appears to be a promising [121, 122].

We adhered to the GRADE basic approaches and rules to assess the quality of a body of evidence, and to develop and report recommendations and make some adjustments.

Rigorous search techniques were implemented, so we thought the possibility of unidentified studies leading to publication bias was rare. We formed recommendations based on many small number trials. Generally, publication bias should be suspected when published evidence is limited to a small number of small trials. However, with new research papers emerging continuously, we believed our recommendation should be interpreted with caution and did not downgrade quality of evidence due to publication bias. Downgrading of analysis was difficult for one outcome across all the studies, because of limited studies, different disease types, interventions, doses, medication courses, and the timing of outcome reports involved in the evidence. Traditional GRADE summary tables for each outcome were presented only for pooled effect of outcomes of interest. For diagnosis questions, studies measuring the impact of testing on patient-important or population-important outcomes were not available, the guideline panels only focused on other studies, such as those involving diagnostic test accuracy which were considered a surrogate outcome for patient-important benefits and harm.

This evidence-based guideline has some limitations. First, the working group did not include patient representatives. Second, since some countries’ government covers all the expense of COVID-19 patients, we did not consider cost-effectiveness for the research questions. As different human resources, funding, or medical supplies, recommendation strength on individuals and communities in low- and middle-income countries maybe different with high-income countries [123]. For some research questions owing to limited evidence at present, we are unable to make strong recommendations, and different countries may make different recommendations in their own contexts. So our recommendations maybe not appropriate for some countries or areas. In low- and middle-income countries, structural inequities and limited resources have added barriers to the utilization of guideline [124]. We did not bring out more specified strategies. Third, because of resource and time limitation, we only include 29 research questions and other meaningful research questions are missed.

Further research is needed on the sources of bias in guideline development within compressed timeframes, in order to work toward the optimal balance between rigor (and development time) and production of a valid, impactful guideline [125]. However, we believe this comprehensive evidence-based guideline will assist clinicians to care COIVD-19 patients better world-wide.

Lastly, this guideline should be implemented based on availability of resources such as supplement of medications, and patient-related factors, including individual values and preferences. When new evidence that can change our recommendations is available, we will update this guideline in time.

## Supplementary information

**Additional file 1.** Conflict of Interest Statement form.

**Additional file 2.** Search resources and Websites.

**Additional file 3.** Search strategies.

**Additional file 4.** PRISMA Diagram for diagnosis section and discharge management section.

**Additional file 5.** PRISMA Diagram for chemoprophylaxis section and treatments section.

**Additional file 6.** Evidence summary tables.

**Additional file 7.** Recommendations list.

## Data Availability

All data generated or analysed during this study are included in this published article and its supplementary information files.

## Abbreviations

ALT: Alanine aminotransferase
ARDS: Acute respiratory distress syndrome
AST: Aspartate aminotransferase
AUC: Area under the curve
AZ: Azithromycin
CI: Confidence interval
COVID-19: Corona virus disease 2019
CQ: Chloroquine
CRP: C-reactive protein
CT: Computed tomography
ECMO: Extracorporeal membrane oxygenation
GGO: Ground-glass opacities
HCQ: Hydroxychloroquine
HFNO: High-flow nasal oxygen
HR: Hazard ratio
ICU: Intensive care unit
IL-6: Interleukin-6
INF-α: Interferon-α
IQR: Interquartile range
LDH: Lactate dehydrogenase
LQG: Lianhua Qingwen Granules
LR: Likelihood ratio
MERS: Middle East Respiratory Syndrome
NIV: Non-invasive ventilation
NRSIs: Non-randomized studies of interventions
OR: Odds ratio
PCT: Procalcitonin
PEEP: Positive end expiratory pressure
PT: Prothrombin time
QPD: Qingfei Paidu Decoction
RCT: Randomized controlled trial
RNA: Ribonucleic acid
RR: Relative risk
RT-PCR: Reverse transcription-polymerase chain reaction
SARS: Severe acute respiratory syndrome
SARS-CoV-2: Severe acute respiratory syndrome coronavirus 2
SMD: Standardized mean difference
SOC: Standard-of-care
TCM: Traditional Chinese Medicine
WBC: White blood cell
WHO: World Health Organization
WMD: Weighted mean difference
