# Supplementary material for: Chemoprophylaxis, diagnosis, treatments, and discharge management of COVID-19: An evidence-based clinical practice guideline (updated version)
Source: Mil Med Res. 2020 Sep 4;7:41. doi: 10.1186/s40779-020-00270-8 (PMC7472403; doi:10.1186/s40779-020-00270-8)
Supplement: Supplementary file 1 — Additional file 1. Conflict of Interest Statement form. [file 40779_2020_270_MOESM1_ESM.doc]

**Evidence-Based Chinese Expert Recommendations on Chemoprophylaxis, Diagnosis, Treatment, and Discharge Management of COVID-19**

**Conflict of Interest Statement form**

| Name |  | | Sex |  | Unit | |  | | |
| --- | --- | --- | --- | --- | --- | --- | --- | --- | --- |
| Rank |  | | Duty |  | | | Professional field | |  |
| Phone number | |  | | | | E-mail | |  | |

- All experts on the guideline steering committee, the guideline consensus group, the guideline development working group and the guideline external review group must disclose all potential conflicts of interest (e.g. all interests that affect or may affect the objectivity and independence of the experts).
- On this conflict of interest statement, you must disclose any commercial, professional or other conflicts of interest related to the subject of this guideline, and any interests that may be affected by the results of this guideline.

**Please copy the "√" next to the "yes" or "no" option. If you choose "yes", please copy "√" after the name of the relevant enterprise or company you choose, such as Shanghai GeneoDx Biotech Co., Ltd√. If the enterprise or company related to you is not listed, please add it to the horizontal line after ‘other enterprise or companies’**.

| **Within the past 24 months: I own stock in companies that have an interest in the guideline.** | |
| --- | --- |
| Novel coronavirus nucleic acid test kit (BGI/GeneoDX/Shanghai Huirui Biotechnology Co.,Ltd/DAAN Gene Co., Ltd. Of Sun Yat-sen university/Shanghai ZJ Bio-Tech Co., Ltd/Shanghai BioGerm Medical Biotechnology Co., Ltd/ Shanghai GeneoDx Biotech Co., Ltd/Other enterprises or companies /BioGerm/ Other enterprises or companies _____________________) | YES□ NO□ |
| Novel coronavirus IgG and IgM antibody test kit (Guangzhou Wondfo Biotech Co., Ltd/Innovita Biological Technology Co., Ltd/Shenzhen YHLO Biotech Co., Ltd/BioMedomics Inc/Vazyme Biotech Co.,Ltd/Hotgen Biotech Co., Ltd/  Other enterprises or companies _____________________) | YES□ NO□ |
| Lopinavir/Ritonavir (Kaletra/Abbvie) (Germany/AbbVie Deutschland GmbH & Co.KG/  Other enterprises or companies _____________________) | YES□ NO□ |
| Arbidol Tablets (China/Hainan Simcere Pharmaceutical Co., Ltd/Jiangsu Wu zhong Pharmaceutical Group Corporation/  Other enterprises or companies _____________________) | YES□ NO□ |
| Interferon (Hapgen) (China/Beijing Tri-Prime Gene Pharmaceutical Co., Ltd/  Other enterprises or companies _____________________) | YES□ NO□ |
| Remdesivir (America/Gilead Sciences, Inc/  Other enterprises or companies _____________________) | YES□ NO□ |
| Favipiravir (Japan/Japan Fuji system; China/Zhejiang Hisun Pharmaceutical Co., LTD/  Other enterprises or companies ______________________) | YES□ NO□ |
| Tocilizumab/atlizumab (Swiss Roche Pharmaceutical Ltd.; Japan ChugaiPharmaManufacturing Co., Ltd.; Bio-Thera Solutions, Ltd/  Other enterprises or companies _____________________) | YES□ NO□ |
| Meplazumab (Pacific Meinuoke Biopharmaceutical/  Other enterprises or companies _____________________) | YES□ NO□ |
| Methylprednisolone (American Pfizer Inc.; Tianjin Kingyao Group Hubei Tianyao Pharmaceutical Co., Ltd; Tianjin Pharmaceutical Jiaozuo Co., Ltd/  Other enterprises or companies _____________________) | YES□ NO□ |
| Ciclesonide (Sanofi-Aventis and ALTANA AG/  Other enterprises or companies _____________________) | YES□ NO□ |
| Chloroquine phosphate (Jinghua Pharmaceutical Group CO., Ltd/Guangzhou Baiyunshan Guanghua Pharmaceutical CO., LTD/Sichuan Shenghe Pharmaceutical Co., Ltd/Sanofi-Aventis U.S. LLC/Impax Laboratories Inc/Purepac Pharmaceutical Co/Hikma Pharmaceuticals USA Inc/MD-Pharm Ltd/Teva Pharmaceutical Industries Ltd/Watson Laboratories Inc/Ipca Laboratories Ltd/Natco Pharma Ltd) Hydroxychloroquine Sulfate (Teva Pharmaceutical Industries Ltd/Sandoz International GmbH/Watson Laboratories Inc/Invatech Health Ltd/Mylan Inc/ Zydus Pharmaceuticals (USA) Inc/Hikma Pharmaceuticals USA Inc/Ipca Laboratories Ltd/Alkaloida Chemical Co. Zrt/Appco Pharma LLC/Lupin Pharmaceuticals, Inc/Amneal Pharmaceuticals Co/Laurus Labs Ltd/Concordia Pharmaceuticals Inc)/  Other enterprises or companies _____________________） | YES□ NO□ |
| ECMO (Medtronic, Inc/Getinge AB/Sorin Group/Terumo Corporation/The Xenios USA® /LivaNova PLC/  Other enterprises or companies _____________________) | YES□ NO□ |
| Blood purification system (Asahi Kasei Medical (HangZhou) Co.,Ltd/Jafron Biomedical Co., Ltd/Jihua Medical Apparatus & Instruments Co., Ltd Guangzhou/ SWS medical group/AOKLAND/WEGO Group/Japan Terumo Corporation/ Infomed S.A/Ube Industries, Ltd/Fresenius Medical Care AG/Daybreak Technology Development Co., Ltd/  Other enterprises or companies _____________________) | YES□ NO□ |
| Lianhua qingwen granule (Beijing Yiling Pharmaceutical Co., Ltd/Shijiazhuang Yiling Pharmaceutical Co., Ltd/  Other enterprises or companies _____________________)  Lianhua qingwen capsules (Shijiazhuang Yiling Pharmaceutical Co., Ltd/  Other enterprises or companies _____________________) | YES□ NO□ |
| Shuanghuanglian oral liquid (Harbin No. 4 Traditional Chinese Medicine Factory Co. , Ltd/Heilongjiang Gratifying Group Co. , Ltd Grat/Heilongjiang Linbao Pharmaceutical Co. , Ltd/Henan New Century Pharmaceutical Co., Ltd/Henan taloph pharmaceutical Co. , Ltd/Heilongjiang Regal Pharmaceutical Co. , Ltd/ HeiLongJiang ZBD Pharmaceutical Co., Ltd/Nanyang Sun Sang Pharmaceutical Co. , Ltd/  Other enterprises or companies _____________________)  Shuanghuanglian injection (Heilongjiang Green Pharmaceutical Co. , Ltd/ Heilongjiang Gerun Pharmaceutical Co., Ltd/Harbin ZBD Pharmaceutical Co. , Ltd/  Other enterprises or companies _____________________) | YES□ NO□ |
| XiYanPing injection (Jiangxi Qingfeng Pharmaceutical Group Co. , Ltd/  Other enterprises or companies _____________________) | YES□ NO□ |
| XueBi Jing injection (Tianjin Chasesun Pharmaceutical Co., Ltd/Other enterprises or companies _____________________) | YES□ NO□ |
| **Within the past 24 months, I have been invited to be a paid consultant for companies that have an interest in the guideline.** | |
| Novel coronavirus nucleic acid test kit (BGI/GeneoDX/Shanghai Huirui Biotechnology Co.,Ltd/DAAN Gene Co., Ltd. Of Sun Yat-sen university/Shanghai ZJ Bio-Tech Co., Ltd/Shanghai BioGerm Medical Biotechnology Co., Ltd/ Shanghai GeneoDx Biotech Co., Ltd/Other enterprises or companies /BioGerm/ Other enterprises or companies _____________________) | YES□ NO□ |
| Novel coronavirus IgG and IgM antibody test kit (Guangzhou Wondfo Biotech Co., Ltd/Innovita Biological Technology Co., Ltd/Shenzhen YHLO Biotech Co., Ltd/BioMedomics Inc/Vazyme Biotech Co.,Ltd/Hotgen Biotech Co., Ltd/Other enterprises or companies _____________________) | YES□ NO□ |
| Lopinavir/Ritonavir (Kaletra/Abbvie) (Germany/AbbVie Deutschland GmbH & Co.KG/Other enterprises or companies _____________________) | YES□ NO□ |
| Arbidol Tablets (China/Hainan Simcere Pharmaceutical Co., Ltd/Jiangsu Wu zhong Pharmaceutical Group Corporation/Other enterprises or companies _____________________) | YES□ NO□ |
| Interferon (Hapgen) (China/Beijing Tri-Prime Gene Pharmaceutical Co., Ltd/Other enterprises or companies _____________________) | YES□ NO□ |
| Remdesivir (America/Gilead Sciences, Inc/Other enterprises or companies _____________________) | YES□ NO□ |
| Favipiravir (Japan/Japan Fuji system; China/Zhejiang Hisun Pharmaceutical Co., LTD/Other enterprises or companies ______________________) | YES□ NO□ |
| Tocilizumab/atlizumab (Swiss Roche Pharmaceutical Ltd.; Japan ChugaiPharmaManufacturing Co., Ltd.; Bio-Thera Solutions, Ltd/Other enterprises or companies _____________________) | YES□ NO□ |
| Meplazumab (Pacific Meinuoke Biopharmaceutical/Other enterprises or companies _____________________) | YES□ NO□ |
| Methylprednisolone (American Pfizer Inc.; Tianjin Kingyao Group Hubei Tianyao Pharmaceutical Co., Ltd; Tianjin Pharmaceutical Jiaozuo Co., Ltd/Other enterprises or companies _____________________) | YES□ NO□ |
| Ciclesonide (Sanofi-Aventis and ALTANA AG/Other enterprises or companies _____________________) | YES□ NO□ |
| Chloroquine phosphate (Jinghua Pharmaceutical Group CO., Ltd/Guangzhou Baiyunshan Guanghua Pharmaceutical CO., LTD/Sichuan Shenghe Pharmaceutical Co., Ltd/Sanofi-Aventis U.S. LLC/Impax Laboratories Inc/Purepac Pharmaceutical Co/Hikma Pharmaceuticals USA Inc/MD-Pharm Ltd/Teva Pharmaceutical Industries Ltd/Watson Laboratories Inc/Ipca Laboratories Ltd/Natco Pharma Ltd) Hydroxychloroquine Sulfate (Teva Pharmaceutical Industries Ltd/Sandoz International GmbH/Watson Laboratories Inc/Invatech Health Ltd/Mylan Inc/ Zydus Pharmaceuticals (USA) Inc/Hikma Pharmaceuticals USA Inc/Ipca Laboratories Ltd/Alkaloida Chemical Co. Zrt/Appco Pharma LLC/Lupin Pharmaceuticals, Inc/Amneal Pharmaceuticals Co/Laurus Labs Ltd/Concordia Pharmaceuticals Inc)/Other enterprises or companies _____________________） | YES□ NO□ |
| ECMO (Medtronic, Inc/Getinge AB/Sorin Group/Terumo Corporation/The Xenios USA® /LivaNova PLC/Other enterprises or companies _____________________) | YES□ NO□ |
| Blood purification system (Asahi Kasei Medical (HangZhou) Co.,Ltd/Jafron Biomedical Co., Ltd/Jihua Medical Apparatus & Instruments Co., Ltd Guangzhou/ SWS medical group/AOKLAND/WEGO Group/Japan Terumo Corporation/ Infomed S.A/Ube Industries, Ltd/Fresenius Medical Care AG/Daybreak Technology Development Co., Ltd/Other enterprises or companies _____________________) | YES□ NO□ |
| Lianhua qingwen granule (Beijing Yiling Pharmaceutical Co., Ltd/Shijiazhuang Yiling Pharmaceutical Co., Ltd/Other enterprises or companies _____________________)  Lianhua qingwen capsules (Shijiazhuang Yiling Pharmaceutical Co., Ltd/Other enterprises or companies _____________________) | YES□ NO□ |
| Shuanghuanglian oral liquid (Harbin No. 4 Traditional Chinese Medicine Factory Co. , Ltd/Heilongjiang Gratifying Group Co. , Ltd Grat/Heilongjiang Linbao Pharmaceutical Co. , Ltd/Henan New Century Pharmaceutical Co., Ltd/Henan taloph pharmaceutical Co. , Ltd/Heilongjiang Regal Pharmaceutical Co. , Ltd/ HeiLongJiang ZBD Pharmaceutical Co., Ltd/Nanyang Sun Sang Pharmaceutical Co. , Ltd/ Other enterprises or companies _____________________)  Shuanghuanglian injection (Heilongjiang Green Pharmaceutical Co. , Ltd/ Heilongjiang Gerun Pharmaceutical Co., Ltd/Harbin ZBD Pharmaceutical Co. , Ltd/Other enterprises or companies _____________________) | YES□ NO□ |
| XiYanPing injection (Jiangxi Qingfeng Pharmaceutical Group Co. , Ltd/Other enterprises or companies _____________________) | YES□ NO□ |
| XueBi Jing injection (Tianjin Chasesun Pharmaceutical Co., Ltd/Other enterprises or companies _____________________) | YES□ NO□ |
| **Within the past 24 months, I have received research funding from companies that have an interest in the guideline.** | |
| Novel coronavirus nucleic acid test kit (BGI/GeneoDX/Shanghai Huirui Biotechnology Co.,Ltd/DAAN Gene Co., Ltd. Of Sun Yat-sen University/Shanghai ZJ Bio-Tech Co., Ltd/Shanghai BioGerm Medical Biotechnology Co., Ltd/ Shanghai GeneoDx Biotech Co., Ltd/Other enterprises or companies /BioGerm/ Other enterprises or companies _____________________) | YES□ NO□ |
| Novel coronavirus IgG and IgM antibody test kit (Guangzhou Wondfo Biotech Co., Ltd/Innovita Biological Technology Co., Ltd/Shenzhen YHLO Biotech Co., Ltd/BioMedomics Inc/Vazyme Biotech Co.,Ltd/Hotgen Biotech Co., Ltd/Other enterprises or companies _____________________) | YES□ NO□ |
| Lopinavir/Ritonavir (Kaletra/Abbvie) (Germany/AbbVie Deutschland GmbH & Co.KG/Other enterprises or companies _____________________) | YES□ NO□ |
| Arbidol Tablets (China/Hainan Simcere Pharmaceutical Co., Ltd/Jiangsu Wu zhong Pharmaceutical Group Corporation/Other enterprises or companies _____________________) | YES□ NO□ |
| Interferon (Hapgen) (China/Beijing Tri-Prime Gene Pharmaceutical Co., Ltd/Other enterprises or companies _____________________) | YES□ NO□ |
| Remdesivir (America/Gilead Sciences, Inc/Other enterprises or companies _____________________) | YES□ NO□ |
| Favipiravir (Japan/Japan Fuji system; China/Zhejiang Hisun Pharmaceutical Co., LTD/Other enterprises or companies ______________________) | YES□ NO□ |
| Tocilizumab/atlizumab (Swiss Roche Pharmaceutical Ltd.; Japan ChugaiPharmaManufacturing Co., Ltd.; Bio-Thera Solutions, Ltd/Other enterprises or companies _____________________) | YES□ NO□ |
| Meplazumab (Pacific Meinuoke Biopharmaceutical/Other enterprises or companies _____________________) | YES□ NO□ |
| Methylprednisolone (American Pfizer Inc.; Tianjin Kingyao Group Hubei Tianyao Pharmaceutical Co., Ltd; Tianjin Pharmaceutical Jiaozuo Co., Ltd/Other enterprises or companies _____________________) | YES□ NO□ |
| Ciclesonide (Sanofi-Aventis and ALTANA AG/Other enterprises or companies _____________________) | YES□ NO□ |
| Chloroquine phosphate (Jinghua Pharmaceutical Group CO., Ltd/Guangzhou Baiyunshan Guanghua Pharmaceutical CO., LTD/Sichuan Shenghe Pharmaceutical Co., Ltd/Sanofi-Aventis U.S. LLC/Impax Laboratories Inc/Purepac Pharmaceutical Co/Hikma Pharmaceuticals USA Inc/MD-Pharm Ltd/Teva Pharmaceutical Industries Ltd/Watson Laboratories Inc/Ipca Laboratories Ltd/Natco Pharma Ltd) Hydroxychloroquine Sulfate (Teva Pharmaceutical Industries Ltd/Sandoz International GmbH/Watson Laboratories Inc/Invatech Health Ltd/Mylan Inc/ Zydus Pharmaceuticals (USA) Inc/Hikma Pharmaceuticals USA Inc/Ipca Laboratories Ltd/Alkaloida Chemical Co. Zrt/Appco Pharma LLC/Lupin Pharmaceuticals, Inc/Amneal Pharmaceuticals Co/Laurus Labs Ltd/Concordia Pharmaceuticals Inc)/Other enterprises or companies _____________________） | YES□ NO□ |
| ECMO (Medtronic, Inc/Getinge AB/Sorin Group/Terumo Corporation/The Xenios USA® /LivaNova PLC/Other enterprises or companies _____________________) | YES□ NO□ |
| Blood purification system (Asahi Kasei Medical (HangZhou) Co.,Ltd/Jafron Biomedical Co., Ltd/Jihua Medical Apparatus & Instruments Co., Ltd Guangzhou/ SWS medical group/AOKLAND/WEGO Group/Japan Terumo Corporation/ Infomed S.A/Ube Industries, Ltd/Fresenius Medical Care AG/Daybreak Technology Development Co., Ltd/Other enterprises or companies _____________________) | YES□ NO□ |
| Lianhua qingwen granule (Beijing Yiling Pharmaceutical Co., Ltd/Shijiazhuang Yiling Pharmaceutical Co., Ltd/Other enterprises or companies _____________________)  Lianhua qingwen capsules (Shijiazhuang Yiling Pharmaceutical Co., Ltd/Other enterprises or companies _____________________) | YES□ NO□ |
| Shuanghuanglian oral liquid (Harbin No. 4 Traditional Chinese Medicine Factory Co. , Ltd/Heilongjiang Gratifying Group Co. , Ltd Grat/Heilongjiang Linbao Pharmaceutical Co. , Ltd/Henan New Century Pharmaceutical Co., Ltd/Henan taloph pharmaceutical Co. , Ltd/Heilongjiang Regal Pharmaceutical Co. , Ltd/ HeiLongJiang ZBD Pharmaceutical Co., Ltd/Nanyang Sun Sang Pharmaceutical Co. , Ltd/ Other enterprises or companies _____________________)  Shuanghuanglian injection (Heilongjiang Green Pharmaceutical Co. , Ltd/ Heilongjiang Gerun Pharmaceutical Co., Ltd/Harbin ZBD Pharmaceutical Co. , Ltd/Other enterprises or companies _____________________) | YES□ NO□ |
| XiYanPing injection (Jiangxi Qingfeng Pharmaceutical Group Co. , Ltd/Other enterprises or companies _____________________) | YES□ NO□ |
| XueBi Jing injection (Tianjin Chasesun Pharmaceutical Co., Ltd/Other enterprises or companies _____________________) | YES□ NO□ |
| **In the past 2-3 months, I have published academic papers related to COVID-19.** | **YES□ NO□** |
| **If your answer is "yes", please indicate what is relevant/ what it relates to** | |

If you are currently participating in or hosting a COVID-19 related study, please fill in the project name in the box below.

|  |
| --- |

In addition to the above, within the past 24 months, is there anything else that you need to declare, please fill in the box below.

|  |
| --- |

In addition to the above, within the past 24 months, if your immediate family members (spouse and children) have conflicts of interest related to the above statement, please fill in the box below.

|  |
| --- |

**Informed about the conflict of interest statement**: I agree to make the above completed content public to other guideline development members, and agree that the statement of interest form will be published in the guideline.

**Statement**: I promise that what I have stated is true and complete. If the information I have stated above changes at any time, I will promptly inform the guideline secretary group and complete a new statement of interest form.

**Signed: Date:**
