# Supplementary material for: Chemoprophylaxis, diagnosis, treatments, and discharge management of COVID-19: An evidence-based clinical practice guideline (updated version)
Source: Mil Med Res. 2020 Sep 4;7:41. doi: 10.1186/s40779-020-00270-8 (PMC7472403; doi:10.1186/s40779-020-00270-8)
Supplement: Supplementary file 2 — Additional file 2. Search resources and Websites. [file 40779_2020_270_MOESM2_ESM.docx]

**Search resources and Websites**

| **Search Resources** | **Websites** |
| --- | --- |
| PubMed | *https://pubmed.ncbi.nlm.nih.gov/* |
| Embase | *https://www.embase.com/login* |
| Cochrane library | *https://www.cochranelibrary.com/* |
| CNKI | *https://www.cnki.net/* |
| Wanfang Database | *http://www.wanfangdata.com.cn/index.html* |
| The New England Journal of Medicine | *https://www.nejm.org/coronavirus?query=main_nav_lg* |
| The Lancet | *https://www.thelancet.com/coronavirus* |
| JAMA | *https://jamanetwork.com/journals/jama/pages/coronavirus-alert* |
| BMJ | *https://www.bmj.com/coronavirus* |
| Nature | *https://www.springernatur e.com/gp/researchers/campaigns/coronavirus* |
| Lancet Infectious Diseases | *https://www.thelancet.com/journals/laninf/home* |
| Lancet Respiratory Medicine | *https://www.thelancet.com/journals/lanres/home* |
| Jama Internal Medicine | *https://jamanetwork.com/journals/jamainternalmedicine* |
|  |  |
| Annals of Internal Medicine | *https://annals.org/aim* |
| Clinical Infectious Diseases | *https://academic.oup.com/cid* |
| Journal of Infection | *https://www.sciencedirect.com/journal/journal-of-infection* |
| American Journal of Respiratory And Critical Care Medicine | *https://www.atsjournals.org/journal/ajrccm* |
| Chest | *https://www.chest.ac.uk/* |
| European Respiratory Journal | https://erj.ersjournals.com/ |
| Chinese Journal of Tuberculosis and Respiratory Diseases | *http://www.lung.org.cn/* |
| Chinese Journal of Epidemiology | *http://chinaepi.icdc.cn/zhlxbx/ch/index.aspx* |
| Unified retrieval port of Chinese series journals | *http://medjournals.cn/index.do;jsessionid=50FCCC54EF12BDDD0020F5974DAE57AC* |
| WHO | *https://www.who.int/* |
| National Health Commission  of the People’s Republic of China | *http://www.nhc.gov.cn/* |
| NICE (National  Institute for Health and Clinical Excellence) | *https://www.nice.org.uk/* |
| GIN(Guidelines International Network) | *https://g-i-n.net/* |
| CDC(Centers for Disease Control  and Prevention) | *https://www.cdc.gov/* |
| bioRxiv | *https://www.biorxiv.org/* |
| medRxiv | *https://www.medrxiv.org/* |
| SSRN | *https://www.ssrn.com/index.cfm/en/* |
| chemRxiv | *https://chemrxiv.org/* |
| ChinaXiv | *http://www.chinaxiv.org/home.htm* |
| Chinese clinical trial registry | *http://www.chictr.org.cn/historyversionpuben.aspx?regno=ChiCTR-ROC-17013221* |
| EU clinical trial register | *https://www.clinicaltrialsregister.eu/index.html* |
| International clinical trial registry | *https://clinicaltrials.gov/* |
