# Supplementary material for: Chemoprophylaxis, diagnosis, treatments, and discharge management of COVID-19: An evidence-based clinical practice guideline (updated version)
Source: Mil Med Res. 2020 Sep 4;7:41. doi: 10.1186/s40779-020-00270-8 (PMC7472403; doi:10.1186/s40779-020-00270-8)
Supplement: Supplementary file 3 — Additional file 3. Search strategies. [file 40779_2020_270_MOESM3_ESM.docx]

**Search strategies**

**1 Search for original articles**

**1.1 Diagnosis**

**(1) Clinical manifestation**

#1 "severe acute respiratory syndrome coronavirus 2"[Supplementary Concept] OR COVID-19[Supplementary Concept]

#2 "severe acute respiratory syndrome coronavirus 2"[Title/Abstract] OR SARS-COV-2[Title/Abstract] OR COVID-19[Title/Abstract] OR COVID19[Title/Abstract] OR "the 2019 coronavirus"[Title/Abstract] OR "2019 coronavirus"[Title/Abstract] OR "2019 novel coronavirus"[Title/Abstract] OR 2019-nCoV[Title/Abstract] OR "Novel coronavirus pneumonia"[Title/Abstract] OR NCP[Title/Abstract] OR "coronavirus disease"[Title/Abstract] OR "coronavirus disease-19"[Title/Abstract] OR "coronavirus disease 2019"[Title/Abstract] OR "Corona Virus Disease 2019"[Title/Abstract] OR HCoV-19[Title/Abstract] OR SARS2[Title/Abstract]

#3 #1 OR #2

#4 "Signs and Symptoms"[MeSH Terms] OR Fever[MeSH Terms] OR Cough[MeSH Terms] OR Fatigue[MeSH Terms] OR Dyspnea[MeSH Terms] OR Myalgia[MeSH Terms]

#5 "Signs and Symptoms"[Title/Abstract] OR Fever[Title/Abstract] OR "muscle pain"[Title/Abstract] OR Cough[Title/Abstract] OR Fatigue [Title/Abstract] OR Dyspnea[Title/Abstract] OR Myalgia[Title/Abstract] OR "clinical manifestation"[Title/Abstract] OR "clinical manifestations"[Title/Abstract] OR "clinical feature"[Title/Abstract] OR "clinical features"[Title/Abstract] OR "clinical characteristics"[Title/Abstract] OR "clinical symptoms"[Title/Abstract] OR "clinical characters"[Title/Abstract] OR "clinical character"[Title/Abstract] OR "clinical finding"[Title/Abstract] OR "clinical findings"[Title/Abstract] OR "clinical presentation"[Title/Abstract] OR "clinical presentations"[Title/Abstract] OR "clinical signs"[Title/Abstract] OR "difficult breathing"[Title/Abstract] OR "muscular soreness"[Title/Abstract] OR anhelation[Title/Abstract] OR "muscle aches"[Title/Abstract] OR "muscle pain"[Title/Abstract]

#6 #4 OR #5

#7 #3 AND #6

**(2) Imaging examination**

#1 "severe acute respiratory syndrome coronavirus 2"[Supplementary Concept] OR COVID-19[Supplementary Concept]

#2 "severe acute respiratory syndrome coronavirus 2"[Title/Abstract] OR SARS-COV-2[Title/Abstract] OR COVID-19[Title/Abstract] OR COVID19[Title/Abstract] OR "the 2019 coronavirus"[Title/Abstract] OR "2019 coronavirus"[Title/Abstract] OR "2019 novel coronavirus"[Title/Abstract] OR 2019-nCoV[Title/Abstract] OR "Novel coronavirus pneumonia"[Title/Abstract] OR NCP[Title/Abstract] OR "coronavirus disease"[Title/Abstract] OR "coronavirus disease-19"[Title/Abstract] OR "coronavirus disease 2019"[Title/Abstract] OR "Corona Virus Disease 2019"[Title/Abstract] OR HCoV-19[Title/Abstract] OR SARS2[Title/Abstract]

#3 #1 OR #2

#4 "diagnostic imaging"[MeSH Terms] OR "Tomography, X-Ray Computed"[MeSH Terms] OR "Computed Tomography"[MeSH Terms]

#5 "imaging examination"[Title/Abstract] OR "imaging examinations"[Title/Abstract] OR "radiological imaging"[Title/Abstract] OR "radiological imagings"[Title/Abstract] OR "imaging procedure"[Title/Abstract] OR "imaging procedures"[Title/Abstract] OR "imaging finding"[Title/Abstract] OR "imaging findings"[Title/Abstract] OR "diagnostic imaging"[Title/Abstract] OR "diagnostic imagings"[Title/Abstract] OR "CT"[Title/Abstract] OR "Computed Tomography"[Title/Abstract] OR "computer assisted tomography"[Title/Abstract] OR "radiological finding"[Title/Abstract] OR "radiological findings"[Title/Abstract] OR "X ray"[Title/Abstract] OR "chest fluoroscopy"[Title/Abstract] OR "X-Ray"[Title/Abstract]

#6 #4 OR #5

#7 #3 AND #6

**(3) Techniques for laboratory tests**

#1 "severe acute respiratory syndrome coronavirus 2"[Supplementary Concept] OR COVID-19[Supplementary Concept]

#2 "severe acute respiratory syndrome coronavirus 2"[Title/Abstract] OR SARS-COV-2[Title/Abstract] OR COVID-19[Title/Abstract] OR COVID19[Title/Abstract] OR "the 2019 coronavirus"[Title/Abstract] OR "2019 coronavirus"[Title/Abstract] OR "2019 novel coronavirus"[Title/Abstract] OR 2019-nCoV[Title/Abstract] OR "Novel coronavirus pneumonia"[Title/Abstract] OR NCP[Title/Abstract] OR "coronavirus disease"[Title/Abstract] OR "coronavirus disease-19"[Title/Abstract] OR "coronavirus disease 2019"[Title/Abstract] OR "Corona Virus Disease 2019"[Title/Abstract] OR HCoV-19[Title/Abstract] OR SARS2[Title/Abstract]

#3 #1 OR #2

#4 "Clinical Laboratory Techniques"[MeSH Terms] OR "Immunoglobulin M"[MeSH Terms] OR "Reverse Transcriptase Polymerase Chain Reaction"[MeSH Terms] OR "Immunoglobulin G"[MeSH Terms]

#5 "laboratory"[Title/Abstract] OR "lab"[Title/Abstract] OR "Immunoglobulin M"[Title/Abstract] OR "Immunoglobulin G"[Title/Abstract] OR "Reverse Transcriptase Polymerase Chain Reaction"[Title/Abstract] OR "leukocyte count"[Title/Abstract] OR "lymphocyte count"[Title/Abstract] OR "platelet count" [Title/Abstract] OR "prothrombin time"[Title/Abstract] OR sodium[Title/Abstract] OR "lactate dehydrogenases"[Title/Abstract] OR "C-reactive protein"[Title/Abstract] OR CRP[Title/Abstract] OR IgM[Title/Abstract] OR IgG[Title/Abstract] OR "white blood cell count"[Title/Abstract] OR WBC[Title/Abstract] OR ALT[Title/Abstract] OR "neutrophil count"[Title/Abstract] OR D-dimer[Title/Abstract] OR "alanine aminotransferase"[Title/Abstract] OR "aspartate aminotransferase"[Title/Abstract] OR AST[Title/Abstract] OR potassium[Title/Abstract] OR creatinine[Title/Abstract] OR Cr[Title/Abstract] OR procalcitonin[Title/Abstract] OR PCT[Title/Abstract] OR "polymerase chain reaction"[Title/Abstract] OR PCR[Title/Abstract] OR "reverse transcriptase PCR"[Title/Abstract] OR "transcriptase PCR"[Title/Abstract] OR RT-PCR[Title/Abstract]

#6 #4 OR #5

#7 #3 AND #6

**1.2 Prophylactic drug treatments and Treatment for COVID-19**

**(1) Immunotherapy**

#1 "severe acute respiratory syndrome coronavirus 2"[Supplementary Concept] OR COVID-19[Supplementary Concept]

#2 "severe acute respiratory syndrome coronavirus 2"[Title/Abstract] OR SARS-COV-2[Title/Abstract] OR COVID-19[Title/Abstract] OR COVID19[Title/Abstract] OR "the 2019 coronavirus"[Title/Abstract] OR "2019 coronavirus"[Title/Abstract] OR "2019 novel coronavirus"[Title/Abstract] OR 2019-nCoV[Title/Abstract] OR "Novel coronavirus pneumonia"[Title/Abstract] OR NCP[Title/Abstract] OR "coronavirus disease"[Title/Abstract] OR "coronavirus disease-19"[Title/Abstract] OR "coronavirus disease 2019"[Title/Abstract] OR "Corona Virus Disease 2019"[Title/Abstract] OR HCoV-19[Title/Abstract] OR SARS2[Title/Abstract]

#3 #1 OR #2

#4 immunotherapy [MeSH Terms]

#5 immunotherapy [Title/Abstract] OR Tocilizumab[Title/Abstract] OR "monoclonal antibod*"[Title/Abstract] OR atlizumab [Title/Abstract] OR Actemra [Title/Abstract] OR "monoclonal antibod*"[Title/Abstract] OR meplazumab[Title/Abstract]

#6 #4 OR #5

#7 #3 AND #6

**(2) Hormonotherapy**

#1 "severe acute respiratory syndrome coronavirus 2"[Supplementary Concept] OR COVID-19[Supplementary Concept]

#2 "severe acute respiratory syndrome coronavirus 2"[Title/Abstract] OR SARS-COV-2[Title/Abstract] OR COVID-19[Title/Abstract] OR COVID19[Title/Abstract] OR "the 2019 coronavirus"[Title/Abstract] OR "2019 coronavirus"[Title/Abstract] OR "2019 novel coronavirus"[Title/Abstract] OR 2019-nCoV[Title/Abstract] OR "Novel coronavirus pneumonia"[Title/Abstract] OR NCP[Title/Abstract] OR "coronavirus disease"[Title/Abstract] OR "coronavirus disease-19"[Title/Abstract] OR "coronavirus disease 2019"[Title/Abstract] OR "Corona Virus Disease 2019"[Title/Abstract] OR HCoV-19[Title/Abstract] OR SARS2[Title/Abstract]

#3 #1 OR #2

#4 glucocorticoids[MeSH Terms]

#5 Glucocorticoid OR "Glucocorticoid Effect" OR Methylprednisolone OR Corticosteroid OR "Glucorticoid Effects" OR "Effects, Glucorticoid" OR Ciclesonide

#6 #4 OR #5

#7 #3 AND #6

**(3) Antiviral therapy**

#1 "severe acute respiratory syndrome coronavirus 2"[Supplementary Concept] OR COVID-19[Supplementary Concept]

#2 "severe acute respiratory syndrome coronavirus 2"[Title/Abstract] OR SARS-COV-2[Title/Abstract] OR COVID-19[Title/Abstract] OR COVID19[Title/Abstract] OR "the 2019 coronavirus"[Title/Abstract] OR "2019 coronavirus"[Title/Abstract] OR "2019 novel coronavirus"[Title/Abstract] OR 2019-nCoV[Title/Abstract] OR "Novel coronavirus pneumonia"[Title/Abstract] OR NCP[Title/Abstract] OR "coronavirus disease"[Title/Abstract] OR "coronavirus disease-19"[Title/Abstract] OR "coronavirus disease 2019"[Title/Abstract] OR "Corona Virus Disease 2019"[Title/Abstract] OR HCoV-19[Title/Abstract] OR SARS2[Title/Abstract]

#3 #1 OR #2

#4"Antiviral Agents"[MeSH Terms] OR arbidol [Supplementary Concept] OR "lopinavir-ritonavir drug combination"[MeSH Terms] OR remdesivir [Supplementary Concept] OR favipiravir [Supplementary Concept] OR "Interferon beta-1b"[MeSH Terms] OR Interferons[MeSH Terms] OR "Interferon Type I"[MeSH Terms] OR "Interferon-beta"[MeSH Terms] OR "Interferon-alpha"[MeSH Terms] OR "Interferon-gamma"[MeSH Terms] OR "Interferon alpha-2"[MeSH Terms]

#5 Lopinavir [Title/Abstract] OR Ritonavir [Title/Abstract] OR "lopinavir-ritonavir drug combination"[Title/Abstract] OR arbidol [Title/Abstract] OR arbidole [Title/Abstract] OR remdesivir [Title/Abstract] OR GS-5734[Title/Abstract] OR favipiravir [Title/Abstract] OR avigan [Title/Abstract] OR "Interferon Type I"[Title/Abstract] OR Interferon[Title/Abstract] OR "Interferon-beta"[Title/Abstract] OR "Interferon-alpha"[Title/Abstract] OR "Interferon-gamma"[Title/Abstract] OR "Interferon alpha-2"[Title/Abstract] OR "Interferon beta-1b"[Title/Abstract] OR "antiviral agents"[Title/Abstract] OR "antiviral agent"[Title/Abstract] OR "antiviral drug"[Title/Abstract] OR "antiviral drugs"[Title/Abstract]

#6 #4 OR #5

#7 #3 AND #6

**(4) Chloroquine or hydroxychloroquine**

#1 "severe acute respiratory syndrome coronavirus 2"[Supplementary Concept] OR COVID-19[Supplementary Concept]

#2 "severe acute respiratory syndrome coronavirus 2"[Title/Abstract] OR SARS-COV-2[Title/Abstract] OR COVID-19[Title/Abstract] OR COVID19[Title/Abstract] OR "the 2019 coronavirus"[Title/Abstract] OR "2019 coronavirus"[Title/Abstract] OR "2019 novel coronavirus"[Title/Abstract] OR 2019-nCoV[Title/Abstract] OR "Novel coronavirus pneumonia"[Title/Abstract] OR NCP[Title/Abstract] OR "coronavirus disease"[Title/Abstract] OR "coronavirus disease-19"[Title/Abstract] OR "coronavirus disease 2019"[Title/Abstract] OR "Corona Virus Disease 2019"[Title/Abstract] OR HCoV-19[Title/Abstract] OR SARS2[Title/Abstract]

#3 #1 OR #2W

#4 Chloroquine OR Aralen OR hydroxychloroquine OR chloroquine phosphate

#5 #3 AND #4

**(5) Traditional Chinese Medicine (TCM)**

#1 "severe acute respiratory syndrome coronavirus 2"[Supplementary Concept] OR COVID-19[Supplementary Concept]

#2 "severe acute respiratory syndrome coronavirus 2"[Title/Abstract] OR SARS-COV-2[Title/Abstract] OR COVID-19[Title/Abstract] OR COVID19[Title/Abstract] OR "the 2019 coronavirus"[Title/Abstract] OR "2019 coronavirus"[Title/Abstract] OR "2019 novel coronavirus"[Title/Abstract] OR 2019-nCoV[Title/Abstract] OR "Novel coronavirus pneumonia"[Title/Abstract] OR NCP[Title/Abstract] OR "coronavirus disease"[Title/Abstract] OR "coronavirus disease-19"[Title/Abstract] OR "coronavirus disease 2019"[Title/Abstract] OR "Corona Virus Disease 2019"[Title/Abstract] OR HCoV-19[Title/Abstract] OR SARS2[Title/Abstract]

#3 #1 OR #2

#4 "Medicine, Chinese Traditional"[MeSH Terms]

#5 "Qingfei Paidu Decoction"[Title/Abstract] OR TCM[Title/Abstract] OR "Qingfei Paidu Soup"[Title/Abstract] OR "Lianhua Qingwen Capsule"[Title/Abstract] OR "Chinese medicine soup"[Title/Abstract] OR "Traditional Chinese Medicine"[Title/Abstract] OR "the combination of Chinese and Western medicine"[Title/Abstract] OR "Chinese patent medicine"[Title/Abstract] OR "Chinese medicine decoction"[Title/Abstract] OR "Medicine, Chinese Traditional"[Title/Abstract] OR "Chinese Medicine"[Title/Abstract] OR "Chinese medicine preparation"[Title/Abstract] OR "Chinese medicine injection"[Title/Abstract]

#6 #4 OR #5

#7 #3 AND #6

**(6) Treatment for severe or critical patients**

**Blood purification treatment**

#1 "severe acute respiratory syndrome coronavirus 2"[Supplementary Concept] OR COVID-19[Supplementary Concept]

#2 "severe acute respiratory syndrome coronavirus 2"[Title/Abstract] OR SARS-COV-2[Title/Abstract] OR COVID-19[Title/Abstract] OR COVID19[Title/Abstract] OR "the 2019 coronavirus"[Title/Abstract] OR "2019 coronavirus"[Title/Abstract] OR "2019 novel coronavirus"[Title/Abstract] OR 2019-nCoV[Title/Abstract] OR "Novel coronavirus pneumonia"[Title/Abstract] OR NCP[Title/Abstract] OR "coronavirus disease"[Title/Abstract] OR "coronavirus disease-19"[Title/Abstract] OR "coronavirus disease 2019"[Title/Abstract] OR "Corona Virus Disease 2019"[Title/Abstract] OR HCoV-19[Title/Abstract] OR SARS2[Title/Abstract]

#3 #1 OR #2

#4 hemoperfusion [MeSH Terms]

#5 hemofiltration [Title/Abstract] OR hemodialysis [Title/Abstract] OR "blood purification"[Title/Abstract] OR hemoperfusion [Title/Abstract]

#6 #4 OR #5

#7 #3 AND #6

**Plasma from survivors**

#1 "severe acute respiratory syndrome coronavirus 2"[Supplementary Concept] OR COVID-19[Supplementary Concept]

#2 "severe acute respiratory syndrome coronavirus 2"[Title/Abstract] OR SARS-COV-2[Title/Abstract] OR COVID-19[Title/Abstract] OR COVID19[Title/Abstract] OR "the 2019 coronavirus"[Title/Abstract] OR "2019 coronavirus"[Title/Abstract] OR "2019 novel coronavirus"[Title/Abstract] OR 2019-nCoV[Title/Abstract] OR "Novel coronavirus pneumonia"[Title/Abstract] OR NCP[Title/Abstract] OR "coronavirus disease"[Title/Abstract] OR "coronavirus disease-19"[Title/Abstract] OR "coronavirus disease 2019"[Title/Abstract] OR "Corona Virus Disease 2019"[Title/Abstract] OR HCoV-19[Title/Abstract] OR SARS2[Title/Abstract]

#3 #1 OR #2

#4 "Convalescent plasma"[Title/Abstract] OR "plasma from survivors"[Title/Abstract] OR "plasma therapy"[Title/Abstract]

#5 #3 AND #4

**Indications for the use of invasive or noninvasive ventilation**

#1 "severe acute respiratory syndrome coronavirus 2"[Supplementary Concept] OR COVID-19[Supplementary Concept]

#2 "severe acute respiratory syndrome coronavirus 2"[Title/Abstract] OR SARS-COV-2[Title/Abstract] OR COVID-19[Title/Abstract] OR COVID19[Title/Abstract] OR "the 2019 coronavirus"[Title/Abstract] OR "2019 coronavirus"[Title/Abstract] OR "2019 novel coronavirus"[Title/Abstract] OR 2019-nCoV[Title/Abstract] OR "Novel coronavirus pneumonia"[Title/Abstract] OR NCP[Title/Abstract] OR "coronavirus disease"[Title/Abstract] OR "coronavirus disease-19"[Title/Abstract] OR "coronavirus disease 2019"[Title/Abstract] OR "Corona Virus Disease 2019"[Title/Abstract] OR HCoV-19[Title/Abstract] OR SARS2[Title/Abstract]

#3 #1 OR #2

#4 "Noninvasive Ventilation"[MeSH Terms] OR "Intermittent Positive-Pressure Ventilation"[MeSH Terms]

#5 NIV [Title/Abstract] OR NPPV[Title/Abstract] OR CPAP[Title/Abstract] OR BiPAP [Title/Abstract] OR PSV[Title/Abstract] OR IPPV[Title/Abstract] OR "invasive ventilation"[Title/Abstract] OR "Noninvasive Ventilation"[Title/Abstract] OR "Inspiratory Positive Pressure Ventilation"[Title/Abstract]

#6 #4 OR #5

#7 #3 AND #6

**The using indications and withdrawal conditions for extracorporeal membrane oxygenation (ECMO)**

#1 "severe acute respiratory syndrome coronavirus 2"[Supplementary Concept] OR COVID-19[Supplementary Concept]

#2 "severe acute respiratory syndrome coronavirus 2"[Title/Abstract] OR SARS-COV-2[Title/Abstract] OR COVID-19[Title/Abstract] OR COVID19[Title/Abstract] OR "the 2019 coronavirus"[Title/Abstract] OR "2019 coronavirus"[Title/Abstract] OR "2019 novel coronavirus"[Title/Abstract] OR 2019-nCoV[Title/Abstract] OR "Novel coronavirus pneumonia"[Title/Abstract] OR NCP[Title/Abstract] OR "coronavirus disease"[Title/Abstract] OR "coronavirus disease-19"[Title/Abstract] OR "coronavirus disease 2019"[Title/Abstract] OR "Corona Virus Disease 2019"[Title/Abstract] OR HCoV-19[Title/Abstract] OR SARS2[Title/Abstract]

#3 #1 OR #2

#4 "Extracorporeal Membrane Oxygenation"[MeSH Terms]

#5 ECMO[Title/Abstract] OR ECLS[Title/Abstract] OR "extracorporeal life support"[Title/Abstract] OR "Extracorporeal Membrane Oxygenation"[Title/Abstract]

#6 #4 OR #5

#7 #3 AND #6

**1.3 Discharge standards and post-hospital management**

#1 "severe acute respiratory syndrome coronavirus 2"[Supplementary Concept] OR COVID-19[Supplementary Concept]

#2 "severe acute respiratory syndrome coronavirus 2"[Title/Abstract] OR SARS-COV-2[Title/Abstract] OR COVID-19[Title/Abstract] OR COVID19[Title/Abstract] OR "the 2019 coronavirus"[Title/Abstract] OR "2019 coronavirus"[Title/Abstract] OR "2019 novel coronavirus"[Title/Abstract] OR 2019-nCoV[Title/Abstract] OR "Novel coronavirus pneumonia"[Title/Abstract] OR NCP[Title/Abstract] OR "coronavirus disease"[Title/Abstract] OR "coronavirus disease-19"[Title/Abstract] OR "coronavirus disease 2019"[Title/Abstract] OR "Corona Virus Disease 2019"[Title/Abstract] OR HCoV-19[Title/Abstract] OR SARS2[Title/Abstract]

#3 #1 OR #2

#4 "Patient Discharge"[MeSH Terms]

#5 discharge OR discharged

#6 #4 OR #5

#7 #3 AND #6

**2 Search for Guideline or Consensus**

#1 "severe acute respiratory syndrome coronavirus 2"[Supplementary Concept] OR COVID-19[Supplementary Concept]

#2 "severe acute respiratory syndrome coronavirus 2"[Title/Abstract] OR SARS-COV-2[Title/Abstract] OR COVID-19[Title/Abstract] OR COVID19[Title/Abstract] OR "the 2019 coronavirus"[Title/Abstract] OR "2019 coronavirus"[Title/Abstract] OR "2019 novel coronavirus"[Title/Abstract] OR 2019-nCoV[Title/Abstract] OR "Novel coronavirus pneumonia"[Title/Abstract] OR NCP[Title/Abstract] OR "coronavirus disease"[Title/Abstract] OR "coronavirus disease-19"[Title/Abstract] OR "coronavirus disease 2019"[Title/Abstract] OR "Corona Virus Disease 2019"[Title/Abstract] OR HCoV-19[Title/Abstract] OR SARS2[Title/Abstract]

#3 #1 OR #2

#4 "Guidelines as Topic"[MeSH Terms] OR "Guidelines as Topics"[Title/Abstract] OR "Practice Guidelines as Topic"[MeSH Terms] OR Guideline[Publication Type] OR "Health Planning Guidelines"[MeSH Terms] OR "Practice Guideline"[Publication Type] OR Consensus[MeSH Terms]

#5 "Clinical Practice Guideline"[Title/Abstract] OR guideline*[Title/Abstract] OR guidance*[Title/Abstract] OR recommendation*[Title/Abstract] OR "Practice Guidelines"[Title/Abstract] OR CPG*[Title/Abstract] OR consensus[Title/Abstract]

#6 #4 OR #5

#7 #3 AND #6

**3 Search for Systematic Review or Meta-analysis**

#1 "severe acute respiratory syndrome coronavirus 2"[Supplementary Concept] OR COVID-19[Supplementary Concept]

#2 "severe acute respiratory syndrome coronavirus 2"[Title/Abstract] OR SARS-COV-2[Title/Abstract] OR COVID-19[Title/Abstract] OR COVID19[Title/Abstract] OR "the 2019 coronavirus"[Title/Abstract] OR "2019 coronavirus"[Title/Abstract] OR "2019 novel coronavirus"[Title/Abstract] OR 2019-nCoV[Title/Abstract] OR "Novel coronavirus pneumonia"[Title/Abstract] OR NCP[Title/Abstract] OR "coronavirus disease"[Title/Abstract] OR "coronavirus disease-19"[Title/Abstract] OR "coronavirus disease 2019"[Title/Abstract] OR "Corona Virus Disease 2019"[Title/Abstract] OR HCoV-19[Title/Abstract] OR SARS2[Title/Abstract]

#3 #1 OR #2

#4 "meta-analysis as topic"[MeSH Terms] OR "meta-analysis"[Publication Type] OR "systematic reviews as topic"[MeSH Terms] OR "systematic review"[Publication Type]

#5 "meta-analysis"[Title/Abstract] OR "meta-analyses"[Title/Abstract] OR "meta analysis and systematic review*"[Title/Abstract] OR "systematic review*"[Title/Abstract]

#6 #4 OR #5

#7 #3 AND #6
