# Supplementary figures and images for: Chemoprophylaxis, diagnosis, treatments, and discharge management of COVID-19: An evidence-based clinical practice guideline (updated version)
Source: Mil Med Res. 2020 Sep 4;7:41. doi: 10.1186/s40779-020-00270-8 (PMC7472403; doi:10.1186/s40779-020-00270-8)

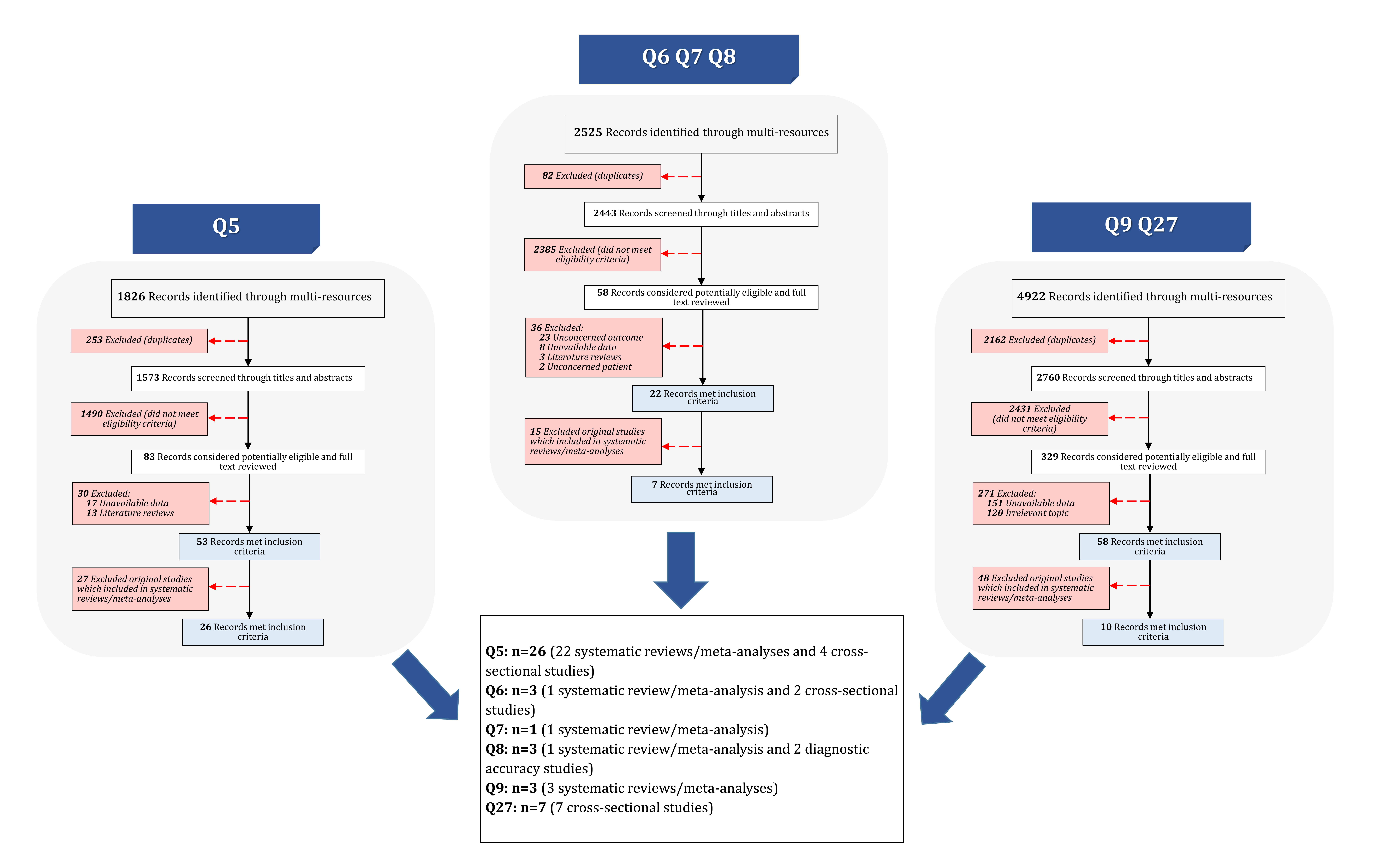

Supplement: Supplementary file 4 — Additional file 4. PRISMA Diagram for diagnosis section and discharge management section. [file 40779_2020_270_MOESM4_ESM.tif]
