# Supplementary material for: Chemoprophylaxis, diagnosis, treatments, and discharge management of COVID-19: An evidence-based clinical practice guideline (updated version)
Source: Mil Med Res. 2020 Sep 4;7:41. doi: 10.1186/s40779-020-00270-8 (PMC7472403; doi:10.1186/s40779-020-00270-8)
Supplement: Supplementary file 6 — Additional file 6. Evidence summary tables. [file 40779_2020_270_MOESM6_ESM.docx]

**Supplementary Table 1 Evidence summary table: antibody test vs. RT-PCR for COVID-19^[43].^**

| Antibody class | Outcome | Number of included studies (sample size) | Study design | Factors that may decrease certainty of evidence | | | | | Quality of evidence |
| --- | --- | --- | --- | --- | --- | --- | --- | --- | --- |
|  |  |  |  | **Risk of bias** | **Indirectness** | **Inconsistency** | **Imprecision** | **Publication bias** |  |
| Time since onset of symptoms (1-7 days) | | | | | | | | | |
| *The sensitivity of IgG, IgM and IgG/IgM was 29.7% (95% CI: 22.1-38.6%), 23.2% (95% CI: 14.9-34.2%) and 30.1% (95% CI: 21.4-40.7%), respectively.* | | | | | | | | | |
| IgG | true positive  false negative | 23 (568) | cross-sectional study | serious^$^ | not serious | not serious | serious^*^ | not found | low |
| IgM | true positive  false negative | 24 (608) | cross-sectional study | serious^$^ | not serious | not serious | serious^*^ | not found | low |
| IgG/IgM | true positive  false negative | 9 (259) | cross-sectional study | serious^$^ | not serious | not serious | serious^*^ | not found | low |
| Time since onset of symptoms (8-14 days) | | | | | | | | | |
| *The sensitivity of IgG, IgM and IgG/IgM was 66.5% (95% CI: 57.9-74.2%), 58.4% (95% CI: 45.5-70.3%) and 72.2% (95% CI: 63.5-79.5%), respectively.* | | | | | | | | | |
| IgG | true positive  false negative | 22 (1200) | cross-sectional study | serious^$^ | not serious | not serious | not serious | not found | moderate |
| IgM | true positive  false negative | 21 (1171) | cross-sectional study | serious^$^ | not serious | not serious | serious^*^ | not found | low |
| IgG/IgM | true positive  false negative | 9 (608) | cross-sectional study | serious^$^ | not serious | not serious | not serious | not found | moderate |
| Time since onset of symptoms (15-21 days) | | | | | | | | | |
| *The sensitivity of IgG, IgM and IgG/IgM was 88.2% (95% CI: 83.5-91.8%), 75.4% (95% CI: 64.3-83.8%) and 91.4% (95% CI: 87.0-94.4%), respectively.* | | | | | | | | | |
| IgG | true positive  false negative | 22 (1110) | cross-sectional study | serious^$^ | not serious | not serious | not serious | not found | moderate |
| IgM | true positive  false negative | 21 (1074) | cross-sectional study | serious^$^ | not serious | not serious | not serious | not found | moderate |
| IgG/IgM | true positive  false negative | 9 (692) | cross-sectional study | serious^$^ | not serious | not serious | serious^*^ | not found | low |
| Time since onset of symptoms (22-35 days) | | | | | | | | | |
| *The sensitivity of IgG, IgM and IgG/IgM was 80.3% (95% CI: 72.4-86.4%), 68.1% (95% CI: 55.0-78.9%) and 96.0% (95% CI: 90.6-98.3%), respectively.* | | | | | | | | | |
| IgG | true positive  false negative | 12 (502) | cross-sectional study | serious^$^ | not serious | not serious | not serious | not found | moderate |
| IgM | true positive  false negative | 11 (507) | cross-sectional study | serious^$^ | not serious | not serious | not serious | not found | moderate |
| IgG/IgM | true positive  false negative | 5 (152) | cross-sectional study | serious^$^ | not serious | not serious | not serious | not found | moderate |
| Time since onset of symptoms (>35 days) | | | | | | | | | |
| *The sensitivity of IgG, IgM and IgG/IgM was 86.7% (95% CI: 79.6-91.7%), 53.9% (95% CI: 38.4-68.6%) and 77.7% (95% CI: 66.0-86.2%), respectively.* | | | | | | | | | |
| IgG | true positive  false negative | 4 (252) | cross-sectional study | serious^$^ | not serious | not serious | serious^*^ | not found | low |
| IgM | true positive  false negative | 4 (215) | cross-sectional study | serious^$^ | not serious | not serious | serious^*^ | not found | low |
| IgG/IgM | true positive  false negative | 2 (153) | cross-sectional study | serious^$^ | not serious | not serious | serious^*^ | not found | low |
| Time since onset of symptoms (all time points) | | | | | | | | | |
| *The specificity of IgG, IgM and IgG/IgM was 99.1% (95% CI: 98.3-99.6%), 98.7% (95% CI: 97.4-99.3%) and 98.7% (95% CI: 97.2-99.4%), respectively.* | | | | | | | | | |
| IgG | true negative  false positive | 44 (6136) | cross-sectional study and cohort study^&^ | serious^$^ | not serious | not serious | not serious | not found | moderate |
| IgM | true negative  false positive | 41 (6103) | cross-sectional study and cohort study^&^ | serious^$^ | not serious | not serious | not serious | not found | moderate |
| IgG/IgM | true negative  false positive | 23 (5761) | cross-sectional study and cohort study^&^ | serious^$^ | not serious | not serious | not serious | not found | moderate |

^&^ The subject of the study did not focus on the diagnostic performance, so the relevant information about the diagnostic test was unclear; ^$^ High risk of included studies; ^*^ Insufficient sample size.

**Supplementary Table 2 Evidence summary table: CT vs. RT-PCR for COVID-19^[44].^**

| Outcome | Number of included studies (sample size) | Study design | Factors that may decrease certainty of evidence | | | | | Quality of evidence |
| --- | --- | --- | --- | --- | --- | --- | --- | --- |
|  |  |  | **Risk of bias** | **Indirectness** | **Inconsistency** | **Imprecision** | **Publication bias** |  |
| *The sensitivity of chest CT was 94% (95% CI: 91-96%).* | | | | | | | | |
| true positive  false negative | 63 (5598) | cohort study^&^, cross-sectional study, case report^&^ and case series^&^. | very serious^$^ | not serious | serious^*^ | not serious | not found | very low |
| *The specificity of chest CT was 37% (95% CI: 26-50%).* | | | | | | | | |
| true negative  false positive | 5 (620) | cross-sectional study | serious^$^ | not serious | serious^*^ | serious^#^ | not found | very low |

^&^ The subject of the study did not focus on the diagnostic performance, so relevant information about the diagnostic test was unclear; ^$^ High risk of included studies; ^*^ There was a considerable level of unexplained heterogeneity; ^#^ Insufficient sample size.

**Supplementary Table 3 Evidence summary table: Remdesivir vs. Placebo for patients with COVID-19 ^[66]^**

|  | **Evidence assessment** | | | | | | | | **Summary of results** | | | | |
| --- | --- | --- | --- | --- | --- | --- | --- | --- | --- | --- | --- | --- | --- |
| **Number of patients (Number of included studies)** | | **Risk of bias** | **Inconsistency** | **Indirectness** | **Imprecision** | **Publication bias** | **Quality of evidence** | **Incidence of events** | | |  | **Absolute effect** | |
|  |  |  |  |  |  |  |  | Remdesivir | | Placebo | *OR/MD* (95%CI) | Remdesivir | Difference |
| *Mortality (the primary outcome), conclusion: there was no difference between two groups in reduce the mortality in patients with COVID-19* | | | | | | | | | | | | | |
| 1141（2 RCTs） | | not serious | serious^#^ | not serious | serious* | not found | low | 54/609 (8.9%) | | 64/532 (12.0 %) | 0.72  (0.39~1.36) | 89/1000 | 31 cases were reduced in per 1000 cases |
| *Clinical improvement (the primary outcome), conclusion: Remdesivir increased the rate of clinical improvement compared to placebo group in patients with COVID-19* | | | | | | | | | | | | | |
| 1084（2 RCTs） | | not serious | not serious | not serious | serious* | not found | moderate | 371/593 (62.6%) | | 254/491 (51.7%) | 1.53 (1.20~1.95) | 626/1000 | 109 cases were increased in per 1000 cases |
| *All adverse event rates (the primary outcome), conclusion: Remdesivir therapy shown lower adverse events compared to placebo group in patients with COVID-19, but there was no difference between two groups in rate of all adverse events.* | | | | | | | | | | | | | |
| 1296（2 RCTs） | | not serious | serious^#^ | not serious | serious* | not found | low | 372/696 (53.4%) | | 363/600 (60.5%) | 0.79  (0.50~1.24) | 534/1000 | 71 cases were reduced in per 1000 cases |
| *Severe adverse event rates (the primary outcome), conclusion: Remdesivir therapy shown lower severe adverse events compared to placebo group in patients with COVID-19* | | | | | | | | | | | | | |
| 1296（2 RCTs） | | not serious | serious^#^ | not serious | serious* | not found | low | 142/696 (20.4%) | | 161/600 (26.8%) | 0.71  (0.55~0.92) | 204/1000 | 64 cases were reduced in per 1000 cases |
| *Time to clinical improvement (the primary outcome), conclusion: Remdesivir reduced the time to clinical improvement compared to placebo group in patients with COVID-19* | | | | | | | | | | | | | |
| 1295（2 RCTs） | | not serious | serious^#^ | not serious | serious* | not found | low | NA | | NA | -3.02  (-4.98~-1.07) | NA | NA |

NA, not applicable; MD, mean difference; OR, Odds ratio; ^#^ High and unexplained heterogeneity, *I^2^*>50%. * the inability to recruit the predetermined study population resulted in study power reduction.

**Supplementary Table 4 Evidence summary table: CQ/HCQ vs. SOC for COVID-19 ^[73]^**

|  | **Evidence assessment** | | | | | | | | **Summary of results** | | | | |
| --- | --- | --- | --- | --- | --- | --- | --- | --- | --- | --- | --- | --- | --- |
| **Number of patients (Number of included studies)** | | **Upgrade factor** | **Risk of bias** | **Inconsistency** | **Indirectness** | **Imprecision** | **Publication bias** | **Quality of evidence** | **Incidence of events** | |  | **Absolute effect** | |
|  |  |  |  |  |  |  |  |  | CQ/HCQ | SOC | RR/MD (95%CI) | CQ/HCQ | Difference |
| *Mortality, conclusion: HCQ could not reduce the mortality in COVID-19.* | | | | | | | | | | | | | |
| 8461 (8 NRSI) | | No | serious^*^ | serious ^#^ | not serious | serious^※^ | not found | very low | 1135/5349 (21.2%) | 644/3112 (20.7%) | 0.98  (0.66~1.46) | 210/1000 | 5 cases were increased in per 1000 cases |
| *Worsening of disease, conclusion: HCQ could not reduce the risk of worsening of disease in COVID-19.* | | | | | | | | | | | | | |
| 4234 (5 NRSI) | | No | serious^*^ | serious ^#^ | not serious | serious^※^ | not found | very low | 397/2193 (18.1%) | 256/2041 (12.5%) | 0.90  (0.47~1.71) | 99/1000 | 56 cases were increased in per 1000 cases |
| Virologic clearance*, conclusion: There was no difference between two groups in virologic clearance time of disease in COVID-19.* | | | | | | | | | | | | | |
| 180 (2 RCTs) | | NA | not serious | serious ^#^ | not serious | serious^※^ | not found | low | 77/90 (85.6%) | 75/90 (83.3%) | 1.02  (0.90~1.15) | 856/1000 | 23 cases were reduced in per 1000 cases |
| 443 (3 NRSI) | | No | serious^*^ | serious ^#^ | not serious | serious^※^ | not found | very low | 217/240 (90.4%) | 152/203 (74.9%) | 1.21  (0.64~2.29) | 904/1000 | 155 cases were reduced in per 1000 cases |
| *Time to fever remission, conclusion: HCQ could not reduce time to fever remission of disease in COVID-19.* | | | | | | | | | | | | | |
| 92 (2 RCTs) | | NA | not serious | serious ^#^ | not serious | serious^※^ | not found | low | NA | NA | -0.51  (-1.49~0.47) | NA | NA |
| 62 (1 NRSI) | | No | serious^*^ | not serious | not serious | serious^※^ | not found | very low | NA | NA | -0.6  (-1.37~0.17) | NA | NA |
| *ECG abnormalities, conclusion: HCQ could not reduce the risk of ECG abnormalities in COVID-19.* | | | | | | | | | | | | | |
| 3739 (2 NRSI) | | No | serious^*^ | serious ^#^ | not serious | serious^※^ | not found | very low | 295/2920 (10.1%) | 45/819 (5.5%) | RR 1.46  (1.04~2.06) | 101/1000 | 46 cases were reduced in per 1000 cases |

CQ/HCQ , Chloroquine/Hydroxychloroquine; SOC, standard of care; RR, Risks ratio; NRSI: non-randomized studies of interventions, most of which were retrospective cohort study; ^*^ CQ/HCQ group did not use random allocation, which may cause implementation bias; ^#^ High and unexplained heterogeneity, *I^2^*>50%; ^※^Imprecision degradation refers to that the number of events is less than 300 or the confidence interval is wide.

**Supplementary Table 5 Evidence summary table: TCZ vs. SOC for severe COVID-19 ^[78]^**

|  | **Evidence assessment** | | | | | | | | **Summary of results** | | | | |
| --- | --- | --- | --- | --- | --- | --- | --- | --- | --- | --- | --- | --- | --- |
| **Number of patients (Number of included studies)** | | **Upgrade factors** | **Risk of bias** | **Inconsistency** | **Indirectness** | **Imprecision** | **Publication bias** | **Quality of evidence** | **Incidence of events** | |  | **Absolute effect** | |
|  |  |  |  |  |  |  |  |  | TCZ | SOC | *OR* (95%CI) | TCZ | Difference |
| *Mortality (the primary outcome), conclusion: TCZ may reduce the mortality in severe COVID-19* | | | | | | | | | | | | | |
| 3641（16 cohort studies） | | No | not serious | serious ^#^ | not serious | not serious | not found | very low^&^ | 258/1153 (22.4%) | 652/2488 (26.2%) | 0.57 (0.36~0.92) | 224/1000 | 38 cases were reduced in per 1000 cases |

TCZ , tocilizumab; SOC, standard of care; OR, Odds ratio; **^&^ No upgrade factors were found**. ^#^ High heterogeneity, *I^2^*=80%.

**Supplementary Table 6 Evidence summary table: Convalescent plasma vs. SOC for severe COVID-19 ^[102]^**

|  | **Evidence assessment** | | | | | | | | **Summary of results** | | | | |
| --- | --- | --- | --- | --- | --- | --- | --- | --- | --- | --- | --- | --- | --- |
| **Number of patients (Number of included studies)** | | **Upgrade factor** | **Risk of bias** | **Inconsistency** | **Indirectness** | **Imprecision** | **Publication bias** | **Quality of evidence** | **Incidence of events** | |  | **Absolute effect** | |
|  |  |  |  |  |  |  |  |  | Convalescent plasma | SOC | RR/HR (95%CI) | Convalescent plasma | Difference |
| *All-cause mortality at hospital discharge (primary outcome), conclusion: Convalescent plasma may not reduce the mortality in severe COVID-19.* | | | | | | | | | | | | | |
| 21 (1 NRSI) | | no | serious* | not serious | not serious | serious^※^ | not found | very low | 5/6 (83.3%) | 14/15 (93.3%) | 0.89 (0.61~1.31) | 833/1000 | 100 cases were reduced in per 1000 cases |
| 86 (1 RCT) | | NA | not serious | not serious | not serious | serious^※^ | not found | moderate | 6/43 (14.0%) | 11/43 (25.6%) | 0.95 (0.20~4.67) | 140/1000 | 116 cases were reduced in per 1000 cases |
| *Time to death (primary outcome), conclusion: Convalescent plasma may not prolong time to death in severe COVID-19* | | | | | | | | | | | | | |
| 103 (1 RCT) | | NA | not serious | not serious | not serious | serious^※^ | not found | moderate | 8/51 (15.7%) | 12/50 (24.0%) | 0.74 (0.30~1.82) | 157/1000 | 83 cases were reduced in per 1000 cases |
| 195 (1 NRSI) | | Large magnitude of an effect | serious* | not serious | not serious | not serious | not found | low | 5/39 (12.8%) | 38/156 (24.4%) | 0.46 (0.22~0.96) | 128/1000 | 116 cases were reduced in per 1000 cases |
| *Improvement of clinical symptoms, assessed by need for respiratory support (Follow-up: 7 days) (secondary outcome), conclusion: Convalescent plasma has no effect on improvement of clinical symptoms at 7 days in severe COVID-19* | | | | | | | | | | | | | |
| 103 (1 RCT) | | NA | not serious | not serious | not serious | serious^※^ | not found | moderate | 5/52 (9.6%) | 5/51  (9.8%) | 0.98 (0.30~3.19) | 96/1000 | 2 cases were reduced in per 1000 cases |
| *Improvement of clinical symptoms, assessed by need for respiratory support (Follow-up: 15 days) (secondary outcome), conclusion: Convalescent plasma has no effect on improvement of clinical symptoms at 15 days in severe COVID-19* | | | | | | | | | | | | | |
| 103 (1 RCT) | | NA | not serious | not serious | not serious | serious^※^ | not found | moderate | 17/52 (32.7%) | 9/51 (17.6%) | 1.85 (0.91~3.77) | 327/1000 | 151 cases were increased in per 1000 cases |
| 195 (1 NRSI) | | No | serious* | not serious | not serious | serious^※^ | not found | very low | 32/39 (82.1%) | 118/156 (75.6%) | 1.08 (0.91~1.29) | 821/1000 | 65 cases were increased in per 1000 cases |
| *Improvement of clinical symptoms, assessed by need for respiratory support (Follow-up: 30 days) (secondary outcome), conclusion: Convalescent plasma has no effect on improvement of clinical symptoms at 30 days in severe COVID-19* | | | | | | | | | | | | | |
| 103 (1 RCT) | | NA | not serious | not serious | not serious | serious^※^ | not found | moderate | 27/52 (51.9%) | 22/51 (43.1%) | 1.20 (0.80~1.81) | 519/1000 | 88 cases were increased in per 1000 cases |

SOC, standard of care; NRSI: non-randomized studies of interventions, most of which were retrospective cohort study; RR, risks ratio; HR, hazard ratio; * Convalescent plasma group did not use blind method, which may cause implementation bias;^※^Imprecision degradation refers to that the number of events is less than 300 or the confidence interval is wide.
