# Supplementary material for: Chemoprophylaxis, diagnosis, treatments, and discharge management of COVID-19: An evidence-based clinical practice guideline (updated version)
Source: Mil Med Res. 2020 Sep 4;7:41. doi: 10.1186/s40779-020-00270-8 (PMC7472403; doi:10.1186/s40779-020-00270-8)
Supplement: Supplementary file 7 — Additional file 7. Recommendations list. [file 40779_2020_270_MOESM7_ESM.docx]

**I. Chemoprophylaxis**

***Question 1: Which kind of agents can prevent COVID-19 in pre-exposure population to reduce SARS-CoV-2 infection?***

There is insufficient evidence to for or against any agents to pre-exposure population (Grade2C).

***Question 2: Which kind of Traditional Chinese Medicine (TCM) agents can prevent COVID-19 in pre-exposure populations to reduce SARS-CoV-2 infection?***

There is no evidence to for or against using any TCM agents for preventing COVID-19 in pre-exposure populations (Ungraded Consensus-Based Statement).

**Question 3：Which kind of agents can prevent COVID-19 in post-exposure population (who contacted or took care of patients with COVID-19) to reduce SARS-CoV-2 infection?**

There is insufficient evidence to for or against any agents to post-exposure population (Grade2C).

***Question 4: Which kind of TCM agents can prevent COVID-19 in post-exposure populations (who contacted or took care of patients with COVID-19) to reduce SARS-CoV-2 infection?***

There is no evidence to for or against using any TCM agents for preventing COVID-19 in in post-exposure populations (Ungraded Consensus-Based Statement).

**II. Diagnosis**

***Question 5:* What are the typical clinical manifestations that can assist clinicians to differentiate SARS-CoV-2 infection from other viral infection in people with suspicious COVID-19?**

The initial symptoms of COVID-19 in ordinary adult patients are most commonly fever and cough (mainly dry cough), often accompanied by fatigue, muscle soreness, dyspnea, expectoration and chest distress. In addition, some patients may present with ocular symptoms, cutaneous symptoms and gastrointestinal symptoms such as diarrhea, nausea, vomiting, olfactory and gustatory dysfunctions. From the perspective of Traditional Chinese Medicine clinical characteristics, the most common tongue body, tongue coating and pulse patterns were red tongue, greasy coating and deep pulse, respectively. If clinicians find that the patient has above-mentioned symptoms during the initial diagnosis, further examination (e.g. CT examination, nucleic acid test etc.) is required to confirm the diagnosis (Grade1A).

Asymptomatic patients generally remain asymptomatic or develop mild symptoms after admission, and clinicians should be cautious about the aggravation of symptoms in these patients. Critical-type patients have severe clinical manifestations and are more prone to fever, dyspnea and abdominal pain, and clinicians should identify the specific manifestations of critical patients as early as possible. (Grade2C).

***Question 6: Comparing with the upper respiratory tract specimens, do lower respiratory tract specimens result in better diagnostic outcomes (such as sensitivity, specificity, positive predictive value [PPV], negative predictive value [NPV], or detection rate) in people with suspicious COVID-19 when performing nucleic acid RT-PCR test?***

If the patient's condition allows (expectorating sputum spontaneously or receiving mechanical ventilation), lower respiratory tract specimens (sputum or broncho- alveolar lavage fluid) can be preferred for testing (Grade2C).

Sampling specimens from lower respiratory tract may result in a higher positive detection rate than those from upper respiratory tract specimens (Ungraded Consensus-Based Statement).

***Question 7: Should IgM and IgG antibody tests be added on to nucleic acid RT-PCR test to have better diagnostic outcomes (i.e., sensitivity, specificity, PPV, NPV) than nucleic acid RT-PCR test alone in people with suspicious COVID-19?***

Clinically diagnosed patients should be tested for SARS-CoV-2 specific IgM and IgG antibodies at 10-14 days after onset of symptoms. IgM and IgG antibodies combined test is better than using IgM or IgG antibody alone (Grade1C).

***Question 8: Can chest computed Tomography (CT) or*** ***x-ray be useful for diagnosing COVID-19 in suspicious people when their nucleic acid RT-PCR tests are negative? If so, which one is more useful?***

Chest CT and x-ray are important alternative tests for RT-PCR test. Suspected COVID-19 patients with typical chest CT and x-ray presentation should be isolated and treated as clinically diagnosed patients (Grade1C).

***Question 9: What are the CT Imaging manifestations that can*** ***assist clinicians to differentiate SARS-CoV-2 pneumonia patients from other viral pneumonia patients?***

The lesions in patients with COVID-19 are mainly distributed either unilaterally or bilaterally in the lower lobes, mostly in peripheral areas. The common imaging findings for COVID-19 are as follows: ground-glass opacities (GGO), interlobular septal thickening, vascular enlargement, crazy paving pattern, subpleural bands, consolidation, and air bronchogram sign. Predominantly GGO pattern is more common than other viral pneumonias, while a mixed pattern of GGO and consolidation is less frequent than other viral pneumonias. COVID-19 pneumonia presented a higher prevalence of peripheral distribution, and involvement of upper and middle lobes than non-COVID pneumonia. Compared to moderate patients, some CT manifestations were more frequent in severe and critical type patients, such as traction bronchiectasis, interlobular septal thickening, consolidation, crazy-paving pattern, reticulation, pleural effusion, and lymphadenopathy (Grade1A).

**III. Treatment**

***Question 10: Should lopinavir-ritonavir be used to treat patients with COVID-19 to improve clinical outcomes?***

We do not suggest offering lopinavir-ritonavir to treat any type patients with COVID-19(Grade2 (C-B)).

***Question 11: Should umifenovir be used to treat patients with COVID-19 to improve clinical outcomes?***

Umifenovir may be considered in COVID-19 treatment (Ungraded Consensus-Based Statement).

***Question 12: Should favipiravir be used to treat patients with COVID-19 to improve clinical outcomes?***

We suggest that favipiravir can be used to treat patients with COVID-19 (Grade2B).

***Question 13: Should interferon be used to treat patients with COVID-19 to improve clinical outcomes?***

Interferon may be considered in COVID-19 treatment (Ungraded Consensus-Based Statement).

***Question 14: Should remdesivir be used to treat COVID-19 patients to improve clinical outcomes?***

We suggest that remdesivir can be used to treat patients with COVID-19 (Grade2(C-B)).

**Question 15: Could a combination of antiviral drugs be used to treat patients with COVID-19 to improve clinical outcomes?**

There is insufficient evidence to for or against using combination of antiviral drugs (Grade2C)**.**

Three or more antiviral drugs should not be used at the same time (Ungraded Consensus-Based Statement).

***Question 16: Should hydroxychloroquine (HCQ)/ chloroquine (CQ) be used to treat patients with COVID-19 to improve clinical outcomes?***

There is inconsistent evidence to for or against using HCQ/CQ in COVID-19 treatment (Grade2C).

We do not suggest using the combination of HCQ and azithromycin (AZ) (Grade2C)**.**

***Question 17: Should interleukin-6 inhibitors be used to treat COVID-19 patients to improve clinical outcomes?***

There is insufficient evidence to support or against using interleukin-6 inhibitors (Grade2C).

***Question 18: Should interleukin-1 inhibitors be used to treat COVID-19 patients to improve clinical outcomes?***

There is insufficient evidence to support or against using interleukin-1 inhibitors (Grade2C).

***Question 19: Should glucocorticoid be used to treat COVID-19 patients to improve clinical outcomes?***

We do not suggest to use glucocorticoid for patients with COVID-19 in general (Grade2B).

When sever or critical COVID-19 patients’ condition deteriorates dramatically, low-dose glucocorticoid with a short course may be considered (Grade2B).

***Question 20: Should Qingfei Paidu Decoction (QPD) be used to treat patients with COVID-19 to improve clinical outcomes?***

*Qingfei Paidu Decoction* may be considered to treat patients with mild or moderate COVID-19 (Ungraded Consensus-Based Statement).

***Question 21: Should Lianhua Qingwen Granules/Capsules (TCM) be used to treat patients with COVID-19 to improve clinical outcomes?***

We suggest that Lianhua Qingwen can be used to treat patients with mild or moderate COVID-19 with conventional therapy (defined as nutritional supportive therapy, symptomatic treatment, antiviral and antibacterial treatment if needed) (Grade2C).

***Question 22: Should convalescent plasma be used to treat COVID-19 patients to improve clinical outcomes?***

There is insufficient evidence to for or against using convalescent plasma to treat severe and critical COVID-19 patients (Grade2B).

***Question 23：Should lung transplantation be used to treat patients with COVID-19 to improve clinical outcomes?***

Lung transplantation maybe a therapeutic option for end-stage patients with COVID-19 (Ungraded Consensus-Based Statement).

***Question 24: What are the indications for the use of invasive or noninvasive ventilation?***

For patients with high-flow nasal oxygen (HFNO) or non-invasive ventilation (NIV) showing no improvement or worsening of their condition or oxygenation index ≤150 mmHg within a short period of time (1-2 hours), endotracheal intubation and invasive mechanical ventilation should be performed promptly (Grade1C).

***Question 25: What are the indications for use of ECMO?***

ECMO is recommended to treat patients with critical COVID-19, and close monitoring of patient’s vital signs is necessary during use. ECMO should be used in the following situations: (1) early stage (such as severe type with a course of less than 7 days) of critical patients with reversible condition; (2) severe hypoxemia: when using optimized PEEP, PaO_2_/FiO_2_ <100 mmHg after using neuromuscular blocker and prone ventilation; (3) excessive compensatory respiratory acidosis (pH<7.15) when using optimized mechanical ventilation; (4) excessive inspiratory stress (plateau pressure >30 cmH_2_O) when using lung protective ventilation; (5) using optimized mechanical ventilation setting, the mechanical power is ≥27 J/min; (6) using the optimized mechanical ventilation setting, there is right heart dysfunction due to acute pulmonary heart disease (Grade1C).

**IV. Discharge Management**

***Question 26: What are the*** ***discharge criteria for COVID-19 patients?***

Patients meeting all the following criteria can be discharged: (1) temperature returned to normal for more than 3 days; (2) respiratory symptoms significantly improved; (3) significant absorption of pulmonary chest lesions on CT imaging; (4) two consecutive negative nucleic acid tests from sputum, nasopharyngeal swabs or other respiratory tract samples (at least 24 hours between samples) (Ungraded Consensus-Based Statement).

***Question 27: What are the imaging findings of*** ***patients where RT-PCR shows positive recovery from COVID-19?***

Most of people have no progressive imaging findings in chest CT of patients where RT-PCR shows positive recovery from COVID-19(Grade2C).

***Question*** ***28: What is the management plan in patients whose RT-PCR retesting shows SARS-CoV-2 positive after discharge?***

After the first discharge, if the RT-PCR test reverts from negative to positive, the patients should be isolated again and may be re-hospitalized based on their clinical characteristics. The effective treatments should be given as early as possible if needed. If the lung image does not have progressive change comparing with that at the first discharge, and patients have three negative RT-PCR tests from sputum and fecal specimens (each≥24 hours apart), the patients can be managed according to the requirements of home isolation and follow-up again (Ungraded Consensus-Based Statement).

***Question 29: Is the RT-PCR retesting needed to monitor COVID-19 patient after discharge?***

Discharged patients may be quarantined for 2 weeks, with follow-up, and PCR tests can be performed at 2 and 4 weeks after discharge (Ungraded Consensus-Based Statement).
